# The interplay between inflammatory cytokines and cardiometabolic disease: bi-directional mendelian randomisation study

**DOI:** 10.1136/bmjmed-2022-000157

**Published:** 2023-02-14

**Authors:** Ville Karhunen, Dipender Gill, Jian Huang, Emmanouil Bouras, Rainer Malik, Mark J Ponsford, Ari Ahola-Olli, Areti Papadopoulou, Saranya Palaniswamy, Sylvain Sebert, Matthias Wielscher, Juha Auvinen, Juha Veijola, Karl-Heinz Herzig, Markku Timonen, Sirkka Keinänen-Kiukaanniemi, Martin Dichgans, Marko Salmi, Sirpa Jalkanen, Terho Lehtimäki, Veikko Salomaa, Olli Raitakari, Simon A Jones, G Kees Hovingh, Konstantinos K Tsilidis, Marjo-Riitta Järvelin, Abbas Dehghan

**Affiliations:** 1 Research Unit of Mathematical Sciences, University of Oulu, Oulu, Finland; 2 Research Unit of Population Health, University of Oulu, Oulu, Finland; 3 Department of Epidemiology and Biostatistics, Imperial College London, London, UK; 4 Singapore Institute for Clinical Sciences (SICS), Agency for Science Technology and Research (A*STAR), Singapore; 5 Department of Hygiene and Epidemiology, Faculty of Medicine, University of Ioannina, Ioannina, Epirus, Greece; 6 Institute for Stroke and Dementia Research (ISD), University Hospital, LMU Faculty of Medicine, Munchen, Bayern, Germany; 7 Division of Immunology, Infection, and Inflammation, Tenovus Institute, Cardiff University, Cardiff, UK; 8 The Stanley Center for Psychiatric Research, Broad Institute of MIT and Harvard, Cambridge, MA, USA; 9 Analytical and Translational Genetics Unit, Massachusetts General Hospital, Boston, MA, USA; 10 Institute for Molecular Medicine Finland, University of Helsinki, Helsinki, Finland; 11 Department of Dermatology, Medical University of Vienna, Vienna, Austria; 12 Department of Psychiatry, Research Unit of Clinical Neuroscience, University of Oulu, Oulu, Finland; 13 Research Unit of Biomedicine, Medical Research Center (MRC), University of Oulu, University Hospital, Oulu, Finland; 14 Department of Gastroenterology and Metabolism, Poznan University of Medical Sciences, Poznan, Poland; 15 Unit of Primary Care, Oulu University Hospital, Oulu, Finland; 16 Healthcare and Social Services of Selänne, Pyhäjärvi, Finland; 17 Munich Cluster for Systems Neurology (SyNergy), Munich, Germany; 18 German Centre for Neurodegenerative Diseases (DZNE), Munich, Germany; 19 MediCity and Institute of Biomedicine, University of Turku, Turku, Finland; 20 Department of Clinical Chemistry, Fimlab Laboratories, and Finnish Cardiovascular Research Center, Faculty of Medicine and Health Technology, Tampere University, Tampere, Finland; 21 Finnish Institute for Health and Welfare, Helsinki, Uusimaa, Finland; 22 Research Centre of Applied and Preventive Cardiovascular Medicine, University of Turku, Turku, Finland; 23 Department of Clinical Physiology and Nuclear Medicine, Turku University Hospital, Turku, Finland; 24 Centre for Population Health Research, University of Turku and Turku University Hospital, Turku, Finland; 25 Department of Vascular Medicine, Academic Medical Center, Amsterdam University Medical Centers, University of Amsterdam, Amsterdam, Noord-Holland, Netherlands; 26 MRC Centre for Environment and Health, School of Public Health, Imperial College London, London, UK; 27 Biocenter Oulu, University of Oulu, Oulu, Finland; 28 Department of Life Sciences, College of Health and Life Sciences, Brunel University London, London, UK; 29 UK Dementia Research Institute, Imperial College London, London, UK

**Keywords:** Epidemiology, Genetics, Cardiology

## Abstract

**Objective:**

To leverage large scale genetic association data to investigate the interplay between circulating cytokines and cardiometabolic traits, and thus identifying potential therapeutic targets.

**Design:**

Bi-directional Mendelian randomisation study.

**Setting:**

Genome-wide association studies from three Finnish cohorts (Northern Finland Birth Cohort 1966, Young Finns Study, or FINRISK study), and genetic association summary statistics pooled from observational studies for expression quantitative trait loci and cardiometabolic traits.

**Participants:**

Data for 47 circulating cytokines in 13 365 individuals from genome-wide association studies, summary statistic data for up to 21 735 individuals on circulating cytokines, summary statistic gene expression data across 49 tissues in 838 individuals, and summary statistic data for up to 1 320 016 individuals on cardiometabolic traits.

**Interventions:**

Relations between circulating cytokines and cardiovascular, anthropometric, lipid, or glycaemic traits (coronary artery disease, stroke, type 2 diabetes mellitus, body mass index, waist circumference, waist to hip ratio, systolic blood pressure, glycated haemoglobin, high density lipoprotein cholesterol, low density lipoprotein cholesterol, total cholesterol, triglycerides, C reactive protein, glucose, fasting insulin, and lifetime smoking).

**Main outcome methods:**

Genetic instrumental variables that are biologically plausible for the circulating cytokines were generated. The effects of cardiometabolic risk factors on concentrations of circulating cytokines, circulating cytokines on other circulating cytokines, and circulating cytokines on cardiometabolic outcomes were investigated.

**Results:**

Genetic evidence (mendelian randomisation P<0.0011) suggests that higher body mass index, waist circumference, smoking, higher concentrations of lipids, and systolic blood pressure increase circulating concentrations of several inflammatory cytokines and C reactive protein. Evidence for causal relations (mendelian randomisation P<0.0011) were noted between circulating cytokines, including a key role of vascular endothelial growth factor on influencing the concentrations of 10 other cytokines. Both mendelian randomisation (P<0.05) and colocalisation (posterior probability >0.5) suggested that coronary artery disease risk is increased by higher concentrations of circulating tumour necrosis factor related apoptosis-inducing ligand (TRAIL), interleukin-1 receptor antagonist (IL1RA), and macrophage colony-stimulating factor (MCSF).

**Conclusion:**

This study offers insight into inflammatory mediators of cardiometabolic risk factors, cytokine signalling cascades, and effects of circulating cytokines on different cardiometabolic outcomes.

**WHAT IS ALREADY KNOWN ON THIS TOPIC**

- Inflammation contributes to the development of cardiovascular disease but the role of circulating cytokines in cardiovascular disease cause is not fully understood

**WHAT THIS STUDY ADDS**

- Novel evidence shows the effects of body mass index, smoking, blood lipids, and systolic blood pressure on the concentrations of several circulating cytokines
- The findings provide support for complex causal relations between cytokines
- Increased concentrations of tumour necrosis factor related apoptosis-inducing ligand (TRAIL), interleukin-1 receptor antagonist (IL1RA), and macrophage colony-stimulating factor (MCSF) increase coronary artery disease risk

**HOW THIS STUDY MIGHT AFFECT RESEARCH, PRACTICE, OR POLICY**

- The findings on TRAIL, IL1RA, and MCSF suggest their prioritisation in further research into putative pharmacological targets for coronary artery disease

## Introduction

Inflammation contributes to the development of cardiovascular disease.^1^ The cytokines, chemokines, growth factors, and interferons (hereafter called cytokines) that contribute to inflammation, therefore, represent potential therapeutic targets for cardiovascular disease.^2^ However, the complex interplay between cardiometabolic risk factors, concentrations of circulating cytokines, and cardiovascular disease outcomes is not fully understood and hinders the identification of therapeutic targets. Traditional observational epidemiological studies exploring these associations suffer from confounding and reverse causation, and randomised controlled trials that can overcome these limitations are often considered unfeasible because of time and resource constraints.

Availability of large scale genetic association data relating to cardiometabolic phenotypes^3–13^ and circulating cytokines^14–17^ has been increasing rapidly. By use of randomly allocated genetic variants that proxy the effect of altering a risk factor of interest, the causal effect of that risk factor on a given outcome in the mendelian randomisation framework can be investigated.^18 19^ For molecular exposures that can be targeted by pharmacological interventions and that can be proxied by genetic variants, mendelian randomisation can be applied to study potential drug effects.^20^

In this study, we performed genome-wide association studies of circulating cytokines. Subsequently, we aimed to use mendelian randomisation to investigate the effect of cardiometabolic risk factors on the concentrations of these circulating cytokines. Then, we pooled data from these genome-wide association studies with publicly available summary statistics and further incorporated gene expression data. We sought to identify biologically plausible genetic variants to proxy the effect of varying circulating cytokine levels. We used mendelian randomisation to investigate cytokine cascades and pathways. Additionally, we aimed to use mendelian randomisation to study the effects of circulating cytokines on cardiometabolic outcomes to identify potential therapeutic targets.

## Methods

### Study design

**Figure 1:**
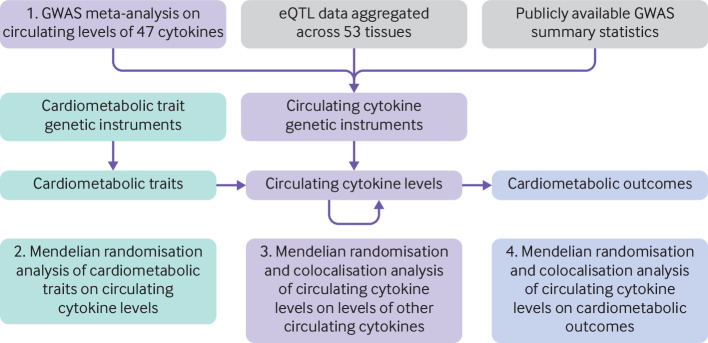
Schematic presentation of the study. The analyses conducted within this study are numbered. eQTL=expression quantitative trait loci. GWAS=genome-wide association study

Figure 1 is a schematic presentation of the study design. Firstly, we conducted genome-wide association studies on 47 cytokines, in up to 13 365 individuals, available in at least one of three Finnish cohorts: Northern Finland Birth Cohort 1966,^21–23^ Young Finns Study,^24^ or FINRISK study^25^ (online supplemental table 1). We used these estimates of genetic associations in a mendelian randomisation to investigate the effects of 15 cardiovascular, anthropometric, lipid, or glycaemic phenotypes (online supplemental table 2) on circulating cytokine concentrations. To generate instrumental variables for the cytokines, we integrated publicly available summary statistics from genome-wide association studies of 21 735 individuals and integrated data for expression quantitative trait loci (eQTL) from 15 201 samples across 49 tissues in 838 individuals. These variables were of biological relevance to the cytokines under consideration through their presence at a relevant gene locus and an association with the corresponding circulating protein, or with the gene expression level. These instruments were used in a two sample mendelian randomisation to investigate the effects of the circulating cytokines on each other. Additionally, the genetic associations were used to investigate the effects of the circulating cytokines on the cardiovascular phenotypes in mendelian randomisation and colocalisation analyses. Further details of the genome-wide association studies, mendelian randomisation, and colocalisation are given in the online supplemental methods.

10.1136/bmjmed-2022-000157.supp1 Supplementary data

**Figure 2:**
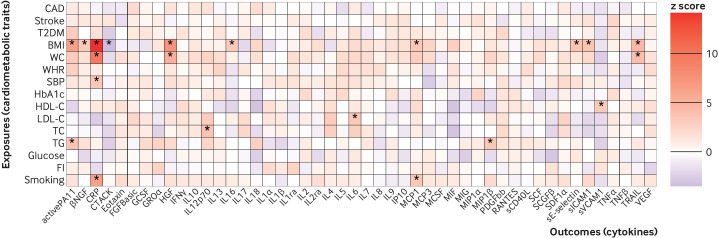
Mendelian randomisation effect size estimates (z scores) of genetically predicted cardiometabolic traits on circulating cytokine levels. The asterisks represent significance after Bonferroni correction for testing of multiple cytokines (P<0.0011 (0.05/number of cytokines)). CAD=coronary artery disease; T2DM=type two diabetes mellitus; BMI=body mass index; WC=waist circumference; WHR=waist to hip ratio; SBP=systolic blood pressure; HDL-C=high density lipoprotein cholesterol; LDL-C=low density lipoprotein cholesterol; TC=total cholesterol; TG=triglycerides; CRP=C reactive protein; FI=fasting insulin. The definitions of the abbreviations for other cytokines are given in online supplemental table 1

### Genome-wide association studies on circulating cytokines

To obtain genetic association estimates for the cytokines considered, genome-wide association studies were conducted separately in each of the three Finnish studies (Northern Finland Birth Cohort 1966, Young Finns Study, FINRISK), assuming an additive genetic model. The results were pooled via an inverse variance weighted fixed effects meta-analysis.

### Instrument selection

The genetic associations for the cardiometabolic traits were obtained from the summary statistics derived from the large scale genome-wide association studies (online supplemental table 2). Instruments for each cardiometabolic trait were selected as single nucleotide polymorphisms (SNPs) that associated with that trait at P<5×10^-8^ (P<1×10^-6^ for fasting insulin) and were uncorrelated (*r* ^2^<0.001). The inverse variance weighted method was applied as the main mendelian randomisation analysis. We also conducted analyses using weighted median, MR-Egger, and MR-PRESSO methods that are more robust to violations of mendelian randomisation assumptions due to horizontal pleiotropy.^19^

To define the instrumental variables for circulating cytokine levels modelled as exposures in a mendelian randomisation, we enhanced our genome-wide association studies data with additional publicly available summary statistics from the INTERVAL study^16^ and SCALLOP consortium,^17^ as well as cross-tissue gene expression associations from the Genotype-Tissue Expression project (version 8).^26^ We generated the genetic instruments using two different criteria (online supplemental table 3). In the first approach, we selected variants within a deviation of plus or minus 500 kilobases of their corresponding gene locus that associated with corresponding circulating cytokine levels at P<1×10^-4^, which we term *cis*-protein QTL (*cis*-pQTL) instruments. In the second approach, we chose variants within plus or minus 500 kilobases of the corresponding gene locus that associated with both gene expression aggregated across tissues at P<1×10^-4^, and circulating cytokine levels at P<0.05, which we term *cis*-expression QTL (*cis*-eQTL) instruments. The cross-tissue gene expression associations were further compared with blood expression associations.

### Mendelian randomisation and colocalisation

Mendelian randomisation analyses investigated the effects of cardiometabolic traits on circulating cytokine concentrations, the effects of circulating cytokines to other cytokine concentrations, and the effects of circulating cytokine concentrations on cardiometabolic phenotypes.

The mendelian randomisation analyses that had circulating cytokine concentrations as exposures were performed by use of the two sets of instruments (*cis*-pQTL and *cis*-eQTL). The ratio method (if one instrument available) or inverse variance weighted method (if two or more instruments available) was applied as the main mendelian randomisation analysis, complemented with weighted median, MR-Egger, and MR-PRESSO methods to investigate whether the results were driven by pleiotropic effects.^19^ In our secondary analyses, we also performed the same mendelian randomisation analysis by selecting instruments as uncorrelated (*r^2^* <0.001) variants from across the genome, associating with the cytokine concentrations at P<5×10^-8^. The Pearson correlation coefficient estimated the similarity between mendelian randomisation estimates obtained for the same exposure and outcome associations as with the two main different instrument selection criteria (*cis*-pQTL and *cis*-eQTL). To account for multiple testing, we applied a Bonferroni correction for the number of outcomes, yielding a P value threshold of 0.05/47=0.0011 when cytokines were outcomes, and 0.05/15=0.0033 when the cardiometabolic phenotypes were outcomes.

For cytokines that generated mendelian randomisation evidence of having causal effects on concentrations of other cytokines, we conducted colocalisation. Specifically, we investigated the posterior probability (PP) of a shared causal variant (PP_shared_) with the exposure and outcome cytokine concentrations at the exposure gene locus, and thus, any evidence for colocalisation would further support a causal association.^27 28^ By contrast, a high PP for distinct causal variants (PP_distinct_) would suggest genetic confounding. PP_shared_+PP_distinct_ >0.5 and PP_shared_ /(PP_shared_+PP_distinct_) >0.5 was considered as evidence for colocalising signals. Similar colocalisation analysis was done for the cytokine and cardiometabolic phenotype pairs with nominal evidence for association in mendelian randomisation (P<0.05).

## Patient and public involvement

Patients and/or the public were not involved in the design, or conduct, or reporting, or dissemination plans of this research. For the mendelian randomisation and colocalisation results, we used anonymised and aggregated summary data, so contacting research participants directly for dissemination of findings was not possible.

## Results

### Genome-wide association studies on inflammatory cytokines

The genome-wide association studies results for circulating levels of the 47 cytokines are presented in online supplemental figure 1. The obtained genetic association estimates were used for the subsequent mendelian randomisation analyses.

### Effect of cardiometabolic traits on inflammatory cytokines

By use of mendelian randomisation, we found positive associations (P<0.0011) for genetically proxied body mass index, waist circumference, systolic blood pressure, high and low density lipoprotein cholesterol concentrations, total cholesterol levels, triglycerides, and smoking liability with circulating concentrations of at least one cytokine (figure 2, online supplemental figure 2, online supplemental tables 4 and 5). The absolute values of these effect sizes (|β|, per 1 standard deviation increase in the genetically proxied exposure) varied from 0.08 (lowest absolute value of the effect sizes) to 0.48 (highest absolute value of the effect sizes). In particular, genetically predicted body mass index was associated with circulating levels of 10 biomarkers, namely, active plasminogen activator inhibitor-1 (PAI1), beta nerve growth factor (βNGF), C reactive protein (CRP), cutaneous T cell attracting chemokine (CTACK), hepatocyte growth factor (HGF), interleukin (IL) 16, monocyte chemoattractant protein-1 (MCP1), soluble E-selectin, soluble intercellular cell adhesion molecule-1 (ICAM1), and tumour necrosis factor related apoptosis-inducing ligand (TRAIL), with |β| for these associations between 0.14 and 0.43. Genetically proxied concentrations of low density lipoprotein cholesterol were associated with circulating levels of IL6 (β=0.12, 95% confidence interval 0.05 to 0.19) (figure 2, online supplemental figure 2, online supplemental table 5).

10.1136/bmjmed-2022-000157.supp2 Supplementary data

### The effect of circulating cytokines on other circulating cytokines

**Figure 3:**
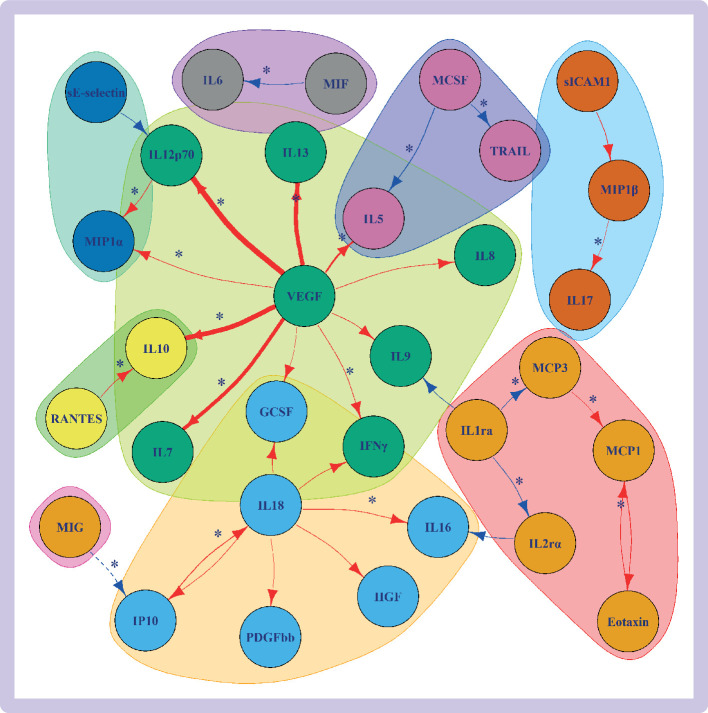
Mendelian randomisation results of genetically predicted cytokine concentrations on levels of other circulating cytokines when considering *cis*-protein quantitative trait loci (solid lines) and *cis*-expression quantitative trait loci (dashed lines) instruments. The results are plotted only for effects with P<0.0011 (0.05/number of cytokines). Red lines indicate positive associations and blue lines indicate negative associations. The thickness of the line represents the absolute value of the effect size (the thicker the line, the larger the absolute value of the effect size). The associations that were also supported by colocalisation results are denoted with an asterisk. The colours of the cytokines represent separate cytokine groups based on applying network community structure algorithm on the graph (supplementary methods). The definitions of the abbreviations are given in online supplemental table 1

The *cis*-pQTL instruments were available for 30 cytokines and *cis*-eQTL instruments for 20 cytokines, with both types of instruments available for 19 cytokines (online supplemental tables 3, 6, and 7). For the *cis*-eQTL instruments, the Pearson correlation between cross-tissue expression z scores and blood expression z scores was 0.38 (95% confidence interval 0.01 to 0.66) (online supplemental table 7). Mendelian randomisation evidence (P<0.0011) showed associations between 34 different cytokine-cytokine pairs when using the *cis*-pQTL or *cis*-eQTL instrument selection criteria, with |β| ranging from 0.07 to 1.71 (figure 3, online supplemental figure 3, online supplemental table 8). The most associations were noted for genetically predicted vascular endothelial growth factor (VEGF) with circulating levels of ten (|β| from 0.14 to 1.71) other cytokines and IL18 with six (|β| from 0.14 to 0.26) other cytokines (figure 3). When comparing the results for the two types of instrumental variables, we found a strong positive correlation between the *cis*-pQTL main mendelian randomisation estimates and the *cis*-eQTL main mendelian randomisation estimates (Pearson correlation r=0.73, online supplemental figure 4).

Colocalisation provided further evidence supporting causality (PP_shared_ /(PP_shared_+PP_distinct_) >0.5) for 20 exposure-outcome pairs of cytokines (figure 3, online supplemental table 9; online supplemental figures 5a-t), notably circulating VEGF concentrations (at the *VEGF* gene) with levels of seven other cytokines: interferon gamma (IFNγ), IL5, IL7, IL10, IL12p70, IL13, and macrophage inflammatory protein-1-alpha (MIP1α).

10.1136/bmjmed-2022-000157.supp3 Supplementary data

### The effect of circulating cytokines on cardiometabolic traits

**Figure 4:**
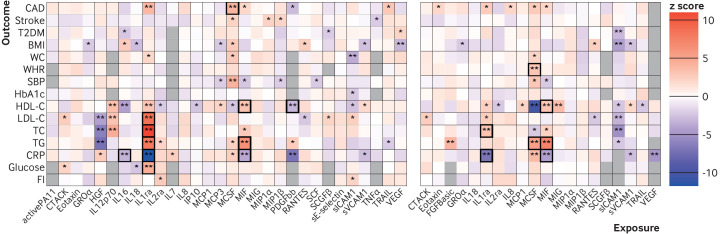
Mendelian randomisation estimates for the effects of genetically predicted cytokine levels on disease outcomes when considering *cis*-pQTL (left) and *cis*-eQTL (right) instruments. Associations with additional colocalisation evidence (PP_shared_+PP_distinct_ >0.5 and PP_shared_ /(PP_shared_+PP_distinct_) >0.5) are highlighted with a box. pQTL=protein quantitative trait loci; eQTL=expression quantitative trait loci. CAD=coronary artery disease; T2DM=type two diabetes mellitus; BMI=body-mass index; WC=waist circumference; WHR=waist to hip ratio; SBP=systolic blood pressure; HDL-C=high density lipoprotein cholesterol; LDL-C=low density lipoprotein cholesterol; TC=total cholesterol; TG=triglycerides; CRP=C reactive protein; FI=fasting insulin; PP=posterior probability. The abbreviations for the cytokines are given in online supplemental table 1. *P<0.05. **P<0.0033

By use of the *cis*-pQTL instrument selection criteria, the evidence of association for 24 cytokine and outcome pairs was strong (P<0.0033; figure 4, online supplemental figure 6, online supplemental table 10). The odds ratio for coronary artery disease risk per 1 standard deviation increase in genetically proxied MCSF was 1.13 (95% confidence interval 1.06 to 1.20). Using the *cis*-eQTL instruments, mendelian randomisation showed evidence of an association for 16 cytokine and outcome pairs (figure 4; odds ratio per 1 standard deviation increase in genetically proxied soluble ICAM1 on type two diabetes mellitus risk was 0.79 (95% confidence interval 0.67 to 0.92).

The *cis*-pQTL and *cis*-eQTL methods provided similar mendelian randomisation estimates (Pearson’s correlation coefficient *r*=0.66, P=4.8×10^-35^, online supplemental figure 7). Analyses of both *cis*-pQTL and *cis*-eQTL instruments showed evidence for association (P<0.0033) for genetically increased IL1RA concentrations with decreased CRP concentrations (*cis*-pQTL β=−0.15 (95% confidence interval −0.18 to −0.13); *cis*-eQTL β=−0.13 (−0.17 to −0.09)) and increased total cholesterol concentrations (*cis*-pQTL β=0.05 (0.04 to 0.06); *cis*-eQTL β=0.05 (0.02 to 0.08)); additionally, for genetically increased macrophage migration inhibitory factor (MIF) concentrations with decreased CRP levels (*cis*-pQTL β=−0.08 (−0.12 to −0.04); *cis*-eQTL β=−0.07 (−0.10 to −0.04)), increased high density lipoprotein cholesterol (*cis*-pQTL β=0.03 (0.01 to 0.04); *cis*-eQTL β=0.03 (0.01 to 0.05)), and increased triglycerides concentrations (*cis*-pQTL β=0.06 (0.04 to 0.08); *cis*-eQTL β=0.07 (0.05 to 0.08)). For the 15 cytokines where three or more genetic instrumental variables were available (online supplemental table 3), weighted median, MR-Egger, and MR-PRESSO sensitivity analyses produced consistent mendelian randomisation estimates to the main inverse-variance weighted analysis (online supplemental methods, online supplemental figure 8, online supplemental table 10). This suggests that pleiotropic associations of the genetic variants are unlikely to be significantly biasing the mendelian randomisation estimates. The mendelian randomisation results that used genome-wide selection of the instruments for the cytokines (online supplemental table 4) provided evidence of association (P<0.0033) for 16 cytokine-outcome pairs. Most notably, genetically proxied circulating concentrations of soluble E-selectin were associated with eight cardiometabolic traits (|β| from 0.01 to 0.07; online supplemental table 11 and online supplemental figure 9).

In colocalisation analyses, evidence suggests a shared causal variant (PP_shared_+PP_distinct_ >0.5 and PP_shared_ /(PP_shared_+PP_distinct_) >0.5) for 19 cytokine and outcome pairs. Notably, IL1RA colocalised with five cardiometabolic traits (CRP, low density lipoprotein cholesterol, total cholesterol, triglycerides, and glucose), and coronary artery disease risk colocalised with MCSF and TRAIL (figure 4, online supplemental table 12; online supplemental figures 10a-s). Thus, MCSF had both mendelian randomisation and colocalisation evidence to support effects on increasing coronary artery disease risk. For the other cytokines with nominal mendelian randomisation evidence (<0.05) for coronary artery disease risk, TRAIL also had evidence for colocalisation with coronary artery disease risk (PP_shared_ /(PP_shared_+PP_distinct_) >0.99). Although colocalisation of IL1RA and coronary artery disease risk showed stronger support for shared causal variant than distinct variants (PP_shared_ /(PP_shared_+PP_distinct_) >0.91), the statistical power was insufficient to show conclusive evidence against genetic confounding (PP_shared_+PP_distinct_=0.42; online supplemental table 12; online supplemental figure 10t).

10.1136/bmjmed-2022-000157.supp4 Supplementary data

## Discussion

### Principal findings

Using novel data and approaches, we offer comprehensive genetic insight into the determinants, cascades, and effects of circulating cytokines in relation to cardiometabolic traits. Our approach generated genetic evidence for effects of obesity measures, hypertension, lipid concentrations, and smoking, on levels of circulating inflammatory cytokines, cytokine cascades underlying systemic responses, and a further three putative therapeutic targets for coronary artery disease (TRAIL, IL1RA, and MCSF).

Obesity, hypertension, and smoking are leading preventable threats to global health.^29^ Our results suggest that higher body mass index increases multiple mediators of inflammation that affect various processes, including thrombosis (via plasminogen activation inhibitor-1), metabolism (HGF), and endothelial dysfunction (MCP1, TRAIL, ICAM1, and soluble E-selectin). These results for body mass index were corroborated by similar associations for waist circumference and waist to hip ratio. Our analyses also suggested that cigarette smoking contributes to elevating CRP and MCP1 levels, higher systolic blood pressure elevates CRP levels, and low density lipoprotein cholesterol elevates circulating IL6 levels. Taken together, these risk factors seem to increase cardiovascular disease pathogenesis at least partly through inflammatory mediators.^30^

### Comparison with other studies

To better understand cytokine regulatory networks, we also examined the associations of genetically predicted circulating cytokines with levels of other cytokines. Our approach suggests complex relations between circulating cytokines, with VEGF appearing as a master regulator. Indeed, VEGF signalling is already targeted clinically in the treatment of certain cancers and ophthalmic conditions.^31 32^ Our findings are consistent with an earlier report identifying VEGF as an upstream controller of IL12p70, IL7, IL10, and IL13.^14^ We replicated and extend these findings to show a wider range of cytokines within this cascade, including drivers of type two immune responses (IL5 and IL13), Jak-STAT cytokine signalling (IFNγ and IL12), and immune modulation (IL10). Although several of these inflammatory cytokines are directly targeted by biological drugs used in routine clinical practice or late stage clinical trials, further research is required to ascertain their potential benefit in the setting of cardiovascular disease. As such, therapeutic targeting of the pathways responsible for the expression or biological signalling of these cytokines might also be possible.

Considering the cardiometabolic outcomes under study, we identified consistent mendelian randomisation and colocalisation evidence for circulating TRAIL increasing coronary artery disease risk; IL1RA and MIF decreasing CRP levels; and IL1RA increasing circulating low density lipoprotein cholesterol, total cholesterol, and glucose concentrations. TRAIL is a ligand involved in initiating apoptosis that has previously been implicated in atherosclerosis.^33–35^ Our current work extends on this previous work by use of genetic data that supports that higher circulating TRAIL concentrations causally increase coronary artery disease risk, further implicating TRAIL as a therapeutic target. We also found evidence that higher body mass index causally increases circulating TRAIL levels, thus implicating TRAIL as a mediator in the effect of body mass index on coronary artery disease risk. Aligning with earlier work, we showed that variants within the *IL1RN* gene that increase IL1RA are positively associated with total cholesterol, low density lipoprotein cholesterol, and glucose, but negatively with CRP concentrations,^36^ which adds support for IL1RA as a therapeutic target in coronary artery disease. Our mendelian randomisation analysis similarly implicated MCSF as a drug target for reducing coronary artery disease risk, triangulating with evidence obtained in studies of mice.^37^ Although the association between IL1RA and coronary artery disease risk was not robustly supported in our colocalisation analysis, the absence of colocalising signals probably reflects insufficient power rather than genetic confounding. Of relevance, anakinra, which is a recombinant and modified human IL1RA protein, is already used in the treatment of rheumatoid arthritis.^38^

The mendelian randomisation results using genome-wide selection for instruments provided distinct results to the main results. In particular, genetically proxied circulating soluble E-selectin levels were associated with eight cardiometabolic traits, mostly driven by strong associations in the *ABO* locus.^15^ Selection of instruments across the full genome allow for detection of *trans*-QTL proxies for cytokines. However, this strategy is also likely to include variants that are not specific only to the relevant cytokine, therefore, potentially mis-specifying the exposure and introducing pleiotropic effects violating the mendelian randomisation assumptions.

### Strengths and limitations of this study

In our analyses, we aimed to maximise the validity of instruments selected to proxy levels of circulating cytokines by considering only variants that were located at the corresponding gene locus, an established strategy for selecting instruments when investigating drug effects.^20 39^ Our novel use of both pQTL and eQTL instrument selection criteria offered complementary evidence, and we further used colocalisation analyses to strengthen the evidence for causal effects. However, our work also has limitations. We could not identify suitable genetic proxies for all considered cytokines, and further we may have missed some associations due to insufficient statistical power and the correction imposed for multiple testing. Of note, we could not identify the support for targeting IL6 and MCP1 to reduce cardiovascular disease risk that has been demonstrated in previous mendelian randomisation studies.^40 41^ The discrepancy for IL6 might be attributable to our approach identifying genetic proxies at the gene for the ligand, rather than its receptor. As a further limitation, mendelian randomisation analyses should not be directly extrapolated to infer the effect of a clinical intervention, as the instruments employed represent the cumulative effect of lifelong genetic predisposition, while a clinical intervention typically represents a discrete event at a particular time point.^42^ Finally, our genetic data were predominantly obtained from individuals of European ancestry and it is unclear whether these findings will extend to individuals of other ancestries.

### Conclusions

By leveraging large scale genetic data, this work offers deeper insight into causal relations between cardiometabolic traits, circulating cytokines, and cardiovascular disease. Our findings replicate previously described therapeutic targets and further identify potential pharmacological opportunities, including perturbation of circulating TRAIL, IL1RA, and MCSF for reducing coronary artery disease risk.

## Data Availability

Data may be obtained from a third party and are not publicly available. The NFBC1966 data are available by application via http://oulu.fi/nfbc/. The genome-wide association study summary statistics generated in this work are publicly available at: https://doi.org/10.5281/zenodo.7215468. The software scripts for the analyses are available from the authors on request.
