# Supplementary material for: The interplay between inflammatory cytokines and cardiometabolic disease: bi-directional mendelian randomisation study
Source: BMJ Med. 2023 Feb 14;2(1):e000157. doi: 10.1136/bmjmed-2022-000157 (PMC9978757; doi:10.1136/bmjmed-2022-000157)
Supplement: Supplementary data [file bmjmed-2022-000157supp001.pdf]

The interplay between inflammatory cytokines and cardiometabolic disease: evidence from Mendelian randomization

Supplementary information

Karhunen V, *et al. BMJMED* 2023; 2:e000157. doi: 10.1136/bmjmed-2022-000157

Contents

The interplay between inflammatory cytokines and cardiometabolic disease: evidence from Mendelian randomization 1

Supplementary Methods 4

Genome-wide association study cohorts 4

Cytokine genome-wide association study 6

Genetic associations and instrument selection for cardiometabolic traits 7

Cytokine instrument selection 8

Mendelian randomization 10

Colocalization 12

Cytokine networks 14

Evidence for causality 14

References 15

Supplementary Figure 1. Circos plot for the cytokine genome-wide association study results in the Finnish cohorts. 24

Supplementary Figure 2. Mendelian Randomization results (point estimates and their 95% confidence intervals) for the effect of genetically predicted cardiometabolic traits on circulating cytokine levels. 26

Supplementary Figure 3. Mendelian randomization estimates for the effects of genetically predicted cytokine levels on other circulating cytokines when considering cis-pQTL (left panel) and cis-eQTL (right panel) instruments. 27

Supplementary Figure 4. Scatter plot comparing Mendelian randomization (MR) estimates for the effects of genetically predicted cytokine levels on other circulating cytokine levels when using the two different instrument selection criteria. 28

Supplementary Figures 5a-t. Colocalization plots for circulating cytokine levels. 29

Supplementary Figure 6. Mendelian Randomization (MR) results (point estimates and their 95% confidence intervals) for the effect of genetically predicted circulating cytokine levels on cardiometabolic phenotypes considered as outcomes. 31

Supplementary Figure 7. Scatter plot comparing Mendelian randomization (MR) estimates for the effects of genetically predicted cytokine levels on cardiometabolic phenotypes considered as outcomes when using the two different instrument selection criteria. 32

Supplementary Figure 8. Scatter plots comparing Mendelian randomization (MR) estimates for the effects of genetically predicted cytokine levels on circulating cytokine levels and cardiometabolic phenotypes considered as outcomes when using different MR methods. 33

Supplementary Figure 9. Mendelian randomization estimates for the effects of genetically predicted cytokine levels on cardiometabolic traits using genome-wide selection for the instruments. 34

Supplementary Figures 10a-t. Colocalization plots for circulating cytokine levels and cardiometabolic traits considered as outcomes. 35

Supplementary Table 1. Cytokines and their data sources for the genome-wide association study. 36

Supplementary Table 2. Sources for genetic association estimates with cardiometabolic traits. 38

Supplementary Table 3. Cytokine coding genes, data sources and the number of instruments with two different criteria. 39

Supplementary Table 4. Variants used as instrumental variables for cardiometabolic traits and cytokines using genome-wide selection. 42

Supplementary Table 5. Table comparing Mendelian randomization (MR) estimates for the effects of genetically predicted cardiometabolic traits on circulating cytokine levels using different MR methods. 42

Supplementary Table 6. Variants used as instrumental variables for cytokines selected by *cis*-protein quantitative trait loci instrument selection. 42

Supplementary Table 7. Variants used as instrumental variables for cytokines selected by *cis*-expression quantitative trait loci instrument selection. 43

Supplementary Table 8. Table comparing Mendelian randomization (MR) estimates for the effects of genetically predicted cytokine levels on other circulating cytokine levels using different MR methods. 43

Supplementary Table 9. Colocalization results for the genetic associations of circulating cytokine levels within ±500kb of the coding gene of the exposure cytokine. 43

Supplementary Table 10. Table comparing Mendelian randomization (MR) estimates for the effects of genetically predicted cytokine levels on cardiometabolic phenotypes considered as outcomes using different MR methods. 44

Supplementary Table 11. Table comparing Mendelian randomization (MR) estimates for the effects of genetically predicted circulating cytokine levels on cardiometabolic traits using genome-wide instrument selection. 44

Supplementary Table 12. Colocalization results for the genetic associations of circulating cytokine levels and cardiometabolic phenotypes considered as outcomes within ±500kb of the coding gene of the cytokine. 44

Karhunen V, *et al. BMJMED* 2023; 2:e000157. doi: 10.1136/bmjmed-2022-000157

Supplementary Methods

Genome-wide association study cohorts

Northern Finland Birth Cohort 1966

Northern Finland Birth Cohort 1966 (NFBC1966) recruited pregnant women with expected date of delivery between 1st January and 31st December 1966[1]. Overall, 12,055 mothers (over 96% of eligible women) were followed from pregnancy onwards, with 12,058 live-born children in the cohort. In the offspring 31-year data collection in 1997, all cohort members with known addresses in either the Northern Finland or Helsinki area were invited to a clinical examination[2]. In total, data were received for 6033 participants, and DNA was successfully extracted for 5753 participants from fasted blood samples. Cytokines were quantified from overnight fasting plasma samples using Bio-Rad's Bio-Plex 200 system (Bio-Rad Laboratories, California, USA) with Milliplex Human Chemokine/Cytokine and CVD/Cytokine kits (Cat# HCYTOMAG-60K-12 and Cat# SPR349; Millipore, St Charles, Missouri, USA) and Bio-Plex Manager Software V.4.3 as previously described[3,4]. Genotyping was conducted using Illumina HumanCNV-370DUO Analysis BeadChip (Illumina, California, USA) and imputed using Haplotype Reference Consortium imputation reference panel.

The Cardiovascular Risk in Young Finns Study

The Cardiovascular Risk in Young Finns (YFS) is an ongoing follow-up study of 3,596 children and adolescents aged 3, 6, 9, 12, 15, or 18 years. The subjects were randomly

chosen from five university cities and their rural surroundings using Finnish population register. The baseline survey was held in 1980 and subsequent follow-up visits involving all five centres have been arranged in 1983, 1986, 1989, 2001, 2007, 2011 and 2017. The latest follow-up included also children and parents of the original participants.

Genotyping have been performed using the blood samples drawn at 2001 follow-up visit. Genotyping was performed with custom-build Illumina 670K array. The custom content replaced some poor performing SNPs on the Human610 BeadChip and added more CNV content after which the customized chip shared 562,643 SNPs with Illumina Human610 chip. Genotyping was performed for 2,556 samples. Prior to imputation, samples and probes with high missingness were excluded (missingness-per-individual > 0.05 and missingness-per-variant > 0.05). To exclude poorly functioning probes, we excluded SNPs deviating from Hardy-Weinberg equilibrium (HWE p-value $< 1\times 10^{-6}$). To exclude related samples, we used $\hat{\pi}$ cut-off of 0.20. The pair with greater missingness was removed. After the QC steps, the data set included 2,443 individuals and 546,674 probes. The imputation was performed with IMPUTE2 software by using 1000 Genomes Phase 3 release as reference panel. After imputation, poorly imputed and rare variants (INFO < 0.7 and MAC < 3) were removed.

Biorad's Bio-Plex Pro Human Cytokine 27-plex Assay and 21-plex Assay were used to quantify circulating concentrations of 48 cytokines from serum samples drawn at 2007 follow-up visit as previously described[5]. Depending on the cytokine, imputed genotypes and cytokine concentrations were available for 116-2019 samples.

FINRISK

FINRISK surveys are population-based cross-sectional studies which began in 1972. A new sample is recruited every five years to monitor the health status of Finnish population. Subset of individual-level data from 1992-2012 surveys is available through THL Biobank. Cytokine quantification for FINRISK1997 and FINRISK2002 samples was performed similarly as in YFS, but quantification was done using EDTA plasma in FINRISK1997 and heparin plasma in FINRISK2002[6]. In FINRISK1997, a custom 20-plex array was used in cytokine quantification. In FINRISK2002, only participants between 51 and 74 years were selected for the analysis. Imputation was performed using 1000 Genomes Phase 3 as reference panel. Poorly imputed variants (INFO < 0.7) and variants with low minor allele count (MAC < 3) were excluded. Depending on the cytokine, imputed genotypes and cytokine concentrations were available for 3440-4613 samples from FINRISK1997 and 843-1705 samples from FINRISK2002.

Cytokine genome-wide association study

We conducted GWAS for 41 cytokines in FINRISK+YFS population and for 16 cytokines in NFBC1966 (Supplementary Table 6)[7]. The data pre-processing and transformations were done in a similar manner to previous GWAS analyses[7,8]. Inverse-normal rank transformation was first applied to the traits, before regressing the transformed measures on age, sex and the first 10 genetic principal components. In contrast to the previous analyses[7,8], we did not add body mass index (BMI) as a covariate, as this could potentially introduce collider bias into consequent MR analyses[9]. The inverse-normal rank transformation was again applied to the residuals of this regression, and these transformed residual estimates were used as response variables in the GWAS.

The GWAS was conducted in each study using an additive genetic model with SNPTEST2 software[10]. The results for variants which showed poor imputation quality (model info < 0.7) or low minor allele frequency (MAF, < 0.05) were discarded. For the ten cytokines available in both NFBC1966 and FINRISK+YFS (Supplementary Table 6), the summary statistics were pooled by inverse variance weighted fixed-effects meta-analysis using Metal software[11].

Genetic associations and instrument selection for cardiometabolic traits

The metabolic traits considered were: coronary artery disease, stroke, type 2 diabetes mellitus, BMI, waist circumference, waist-hip-ratio, systolic blood pressure, glycated haemoglobin, high-density lipoprotein cholesterol, low-density lipoprotein cholesterol, total cholesterol, triglycerides, C-reactive protein, glucose, fasting insulin and lifetime smoking. Genetic association estimates for these traits were obtained from large GWAS of mainly European ancestry participants[12–22] (Supplementary Table 2). We used GWAS summary statistics that were unadjusted for other heritable phenotypes to avoid potential collider bias in MR[9]. Single-nucleotide polymorphisms (SNPs) that associated with the corresponding trait at $P < 5\times10^{-8}$ and were independent ($r^2 < 0.001$) were chosen as instruments to proxy the exposure. For fasting insulin, we relaxed the selection threshold to $P < 1\times10^{-6}$ in order to have at least one instrument available. Clumping was performed using the TwoSampleMR package in R[23]. In clumping, a variant with the lowest p-value is detected within a specific genomic window, and any variants with r^2 above the pre-specified threshold with the lead variant are excluded. This procedure is iteratively

repeated for any other remaining variants, also excluding the lead variant from the previous iteration. The final list of instruments consists of the lead variants for each iteration. We used the window size of 10,000 kilobase pairs, which is the default value in ld_clump() function. To protect against potential reverse causation, any variants from within ±500 kb of the coding gene of the considered outcome cytokine were excluded.

Cytokine instrument selection

We further incorporated information from publicly available genetic associations to improve the instruments for cytokines in MR. These instruments are *a priori* biologically plausible, merging association summary statistics from two other pQTL sources, as well as eQTL data.

We sought GWAS summary statistics from INTERVAL[24] and SCALLOP[25] consortia for the same cytokines that were analysed in our current work. We first tested whether the genetic associations were comparable between our work, INTERVAL, and SCALLOP consortia considering the different measurement methods. Specifically, for each cytokine, we examined the correlation between the beta estimates between our work and INTERVAL study and between our work and SCALLOP consortium based on the SNPs associated with the cytokine at P-value<10^{-5} and r^2<0.1. For 11 cytokines which beta estimates were correlated (regression coefficient P-value < 0.1 of regressing the beta estimates of the current study on the beta estimates of a previous study), we calibrated the beta estimates of the INTERVAL (3 cytokines) and SCALLOP (8 cytokines) consortium to match with the beta estimates from the current studies and performed the meta-analyses. For the rest of the cytokines, we used summary statistics from the GWAS with

the largest sample size (the current studies (26 cytokines), the INTERVAL study (2 cytokines), and SCALLOP consortium (8 cytokines); Supplementary Table 3).

Gene expression GWAS summary statistics were obtained from the Genotype-Tissue Expression (GTEx) project (release version 8), and related to 15,201 samples from a multi-ethnic group of 838 individuals[26]. Results from 49 tissues were pooled using fixed-effects meta-analysis to produce cross-tissue estimates of association with gene expression. To validate the relevance of the cross-tissue *cis*-eQTL instruments, we examined the correlation of the expression Z-scores of the *cis*-eQTL variants with the Z-scores in blood from eQTLGen consortium[27].

Two distinct instrument selection criteria were used to identify variants to proxy the effect of circulating cytokine levels in MR analyses. Firstly, variants located within ±500 kb of the gene locus corresponding to a cytokine under study that also related to circulating levels of that cytokine with association P-value $< 1\times10^{-4}$ were selected. These are referred to as *cis*-pQTL. Secondly, variants located within ±500 kb of the gene locus corresponding to a cytokine under study that related to both expression of that gene at $P < 1\times10^{-4}$ and circulating levels of that cytokine at $P < 0.05$ were selected. These are referred to as *cis*-eQTL. The gene locations were extracted per human genome build 19 (released February 2009) using UCSC Genome Browser (accessed on 18th June 2019) [28]. As we only considered variants from within a biologically relevant genomic locus, we did not use the conventional threshold of $P < 5\times10^{-8}$ (which is usually applied when instruments are selected across the full genome) in our main analysis.

Karhunen V, *et al. BMJMED* 2023; 2:e000157. doi: 10.1136/bmjmed-2022-000157

Only variants for which both exposure and outcome genetic association estimates were available for any given MR analysis were considered as potential instruments for that analysis. All variants were clumped to a pairwise linkage disequilibrium threshold of r^2<0.1 using the TwoSampleMR package in R[23]. The F statistic was calculated as a measure of the strength of the instruments[29]. To protect from potential weak instrument bias, we only used variants with F>10 as instruments[30,31]. As a supplementary analysis, we also report the results considering cytokines as exposures and cardiometabolic traits as outcomes, when selecting the instruments at $P < 5\times10^{-8}$ across the full genome from the GWAS summary statistics of the three Finnish cohorts, clumping at $r^2 < 0.001$.

Mendelian randomization

The ratio method was used to obtain MR estimates, with first order weights used to generate standard errors[32]. Where more than one instrument variant was available for a given analysis, MR estimates obtained from different instruments were pooled using the multiplicative random-effects inverse-variance weighted (IVW) MR method. MR is prone to bias when the genetic variants used as instruments affect the outcome through some pathway that is independent of the exposure under consideration, a phenomenon termed pleiotropy[33]. To explore the possibility of the MR results being driven by such pleiotropic effects, MR methods that make distinct assumptions on the inclusion of pleiotropic variants were performed in sensitivity analyses where three or more instrument variants were available. Specifically, the weighted median, MR-Egger and MR-PRESSO methods were used[34,35]. The weighted median approach orders the MR estimates produced by each instrument by their magnitude weighted for their precision and produces an overall MR estimate based on the median value, with standard error

estimated by bootstrapping[34]. It is a consistent approach when more than half of the weight for the analysis is derived from valid instruments[34]. MR-Egger regresses the variant-outcome associations on the variant-exposure associations, weighted by the precision of the variant-outcome association estimates. The regression slope represents the MR estimate, and presence of directional pleiotropy can be assessed by testing whether the intercept differs from zero[36]. MR-Egger assumes that any pleiotropic effects of the genetic variants are uncorrelated with the variant-exposure associations, and the method may be biased due to outliers[37]. Therefore, we report MR-Egger results only for exposures with ≥10 variants available as instruments. The MR-PRESSO detects outliers using the squared residuals from the regression of the variant-outcome association estimates on the variant-exposure association estimates with the intercept fixed to zero, and repeats such regression-based MR analysis after excluding any identified outlier variants[35].

We accounted for multiple testing by applying a Bonferroni correction for the number of outcomes, resulting to $P<0.05/47 = 0.0011$ when cytokines were considered as outcomes, and $P<0.05/15 = 0.0033$ when considering the cardiometabolic traits as outcomes. The lenient approach to multiple testing correction was taken as the MR results were further validated in colocalization analysis, detailed below. The MR sensitivity analyses were only performed to explore the robustness of the main IVW analysis to potential pleiotropy and as such no multiple testing correction was applied for these.

Karhunen V, *et al. BMJMED* 2023; 2:e000157. doi: 10.1136/bmjmed-2022-000157

Colocalization

The colocalization analysis method 'coloc' proposed by Giambartolomei et al.[38] as applied here investigates whether the data are consistent with a shared variant influencing both exposure and outcome trait. Under the assumption of a maximum of one causal variant per each trait within a genomic locus, the alternative hypotheses are:

H_0: No causal variants in the locus.

H_1: A causal variant on the exposure only.

H_2: A causal variant on the outcome only.

H_3: Distinct causal variants on exposure and outcome.

H_4: A shared causal variant on exposure and outcome.

Given data $D = \left(\hat{\beta}_j \ \ \mathrm{SE}\left(\hat{\beta}_j\right)\right), j = 1, \dots, m$ for *m* variants from GWAS summary statistics, and prior probabilities p_1 for a variant being causal for the exposure, p_2 for a variant being causal for the outcome, and p_{12} for a variant being causal for both exposure and outcome, we can compute the posterior probabilities (PP) for all hypotheses. Specifically, the PP for a shared causal variant (PP_{shared}) is:

$$PP_{\text{shared}} = P(H_4|D) = \frac{P(H_4|D)/P(H_0|D)}{1 + \sum_{k=1}^{4}\left(P(H_k|D)/P(H_0|D)\right)}$$

And the PP for distinct causal variants ($PP_{distinct}$) is:

$$PP_{\text{distinct}} = P(H_3|D) = \frac{P(H_3|D)/P(H_0|D)}{1 + \sum_{k=1}^{4}\left(P(H_k|D)/P(H_0|D)\right)}.$$

Karhunen V, *et al. BMJMED* 2023; 2:e000157. doi: 10.1136/bmjmed-2022-000157

In the above equations,

$$\frac{P(H_1|D)}{P(H_0|D)} = p_1 \times \sum_j ABF_j^{(1)} \qquad \frac{P(H_2|D)}{P(H_0|D)} = p_2 \times \sum_j ABF_j^{(2)}$$

$$\frac{P(H_3|D)}{P(H_0|D)} = p_1 p_2 \times \sum_{j \neq k} ABF_j^{(1)} ABF_k^{(2)} \quad \frac{P(H_4|D)}{P(H_0|D)} = p_{12} \times \sum_j ABF_j^{(1)} ABF_j^{(2)},$$

and ABF is the Approximate Bayes Factor[39]:

$$ABF = \sqrt{\frac{\mathrm{SE}(\hat{\beta})^2 + W}{\mathrm{SE}(\hat{\beta})^2}} \exp\{-\frac{1}{2}\left(\frac{\hat{\beta}}{\mathrm{SE}(\hat{\beta})}\right)^2 \frac{W}{\mathrm{SE}(\hat{\beta}) + W}\},\ \hat{\beta} \sim N(0, W),$$

where W is the prior variance for causal effect sizes, set here as $0.15 \times \mathrm{sd}(X)$ for continuous traits and 0.2 for binary traits (the default values used in coloc).

In colocalization, we are particularly interested in PP_{shared}. We only considered the gene region for the exposure cytokine for exposure-outcome pairs with MR evidence, and thus a high PP_{shared} would provide supporting evidence to our MR results. In contrast, a high $PP_{distinct}$ would imply that the MR results were confounded by LD, that is, the MR evidence of association is caused purely by LD between two distinct variants, one influencing the exposure, and the other influencing the outcome.

Colocalization may suffer from reduced power if the association with the outcome is weak, which is commonly the case when colocalization is done as a sensitivity analysis following MR. Additionally, when colocalization is conducted to examine the possibility of confounding by LD, already in the presence of MR evidence, it is of relevance to examine the probability of a shared causal variant conditioned on there being a causal

variant for both traits. Therefore, we defined colocalization as being present when (i) $PP_{shared} + PP_{distinct} > 0.5$ (to ensure sufficient power), and (ii) $PP_{shared} /(PP_{shared} + PP_{distinct}) > 0.5$ (to support that the posterior probability of a shared causal variant is greater than the posterior probability of distinct causal variants)[40].

We used the effect size estimates and their variances from the cytokine GWAS summary statistics, within ±500kb around the coding gene. The prior probabilities p_1, p_2 and p_{12} were all set at 10^{-4}.

Cytokine networks

To further visualize the clustering of the different cytokines based on the MR results, we applied network community structure algorithm for the cytokine-cytokine pairs that showed MR associations at $P<0.0011$. Briefly, without any prior information on the network structure, each node (cytokine) is initialized with a unique label, and the algorithm proceeds iteratively to label each node (cytokine) by majority voting in its neighborbood[41].

Evidence for causality

In examining the causality of circulating cytokine levels on cardiometabolic phenotypes, we merge the results from both MR and colocalization analyses for an overall summary. We infer *strong evidence for causality* as (i) MR association at $P<0.0033$ using either *cis*-pQTL or *cis*-eQTL instruments for the cytokine considered, with concordant effect size estimates if both types of instruments available, and (ii) colocalization ($PP_{shared} /(PP_{shared} + PP_{distinct}) > 0.5$) within the corresponding genomic locus of circulating protein levels and outcome.

References

1. Rantakallio P. Groups at risk in low birth weight infants and perinatal mortality. *Acta Paediatr Scand* 1969;**193**:Suppl 193:1+.

2. Marjo-Riitta Järvelin, Ulla Sovio, Vanessa King, Liisa Lauren, Baizhuang Xu, Mark I. McCarthy, Anna-Liisa Hartikainen, Jaana Laitinen, Paavo Zitting, Paula Rantakallio, Paul Elliott. Early Life Factors and Blood Pressure at Age 31 Years in the 1966 Northern Finland Birth Cohort. *Hypertension* 2004;**44**:838–846.

3. Saukkonen T, Mutt SJ, Jokelainen J, Saukkonen AM, Raza GS, Karhu T, Harkonen P, Eckel J, Herzig KH, Rajala U, Keinanen-Kiukaanniemi S. Adipokines and inflammatory markers in elderly subjects with high risk of type 2 diabetes and cardiovascular disease. *Sci Rep* 2018;**8**:12816.

4. Palaniswamy S, Gill D, De Silva NM, Lowry E, Jokelainen J, Karhu T, Mutt SJ, Dehghan A, Sliz E, Chasman DI, Timonen M, Viinamäki H, Keinänen-Kiukaanniemi S, Hyppönen E, Herzig K-H, Sebert S, Järvelin M-R. Could vitamin D reduce obesity-associated inflammation? Observational and Mendelian randomization study. *Am J Clin Nutr* 2020;**111**:1036–1047.

5. Santalahti K, Maksimow M, Airola A, Pahikkala T, Hutri-Kähönen N, Jalkanen S, Raitakari OT, Salmi M. Circulating Cytokines Predict the Development of Insulin Resistance in a Prospective Finnish Population Cohort. *J Clin Endocrinol Metab* 2016;**101**:3361–3369.

6. Santalahti K, Havulinna A, Maksimow M, Zeller T, Blankenberg S, Vehtari A, Joensuu H, Jalkanen S, Salomaa V, Salmi M. Plasma levels of hepatocyte growth factor and placental growth factor predict mortality in a general population: a prospective cohort study. *J Intern Med* 2017;**282**:340–352.

7. Sliz E, Kalaoja M, Ahola-Olli A, Raitakari O, Perola M, Salomaa V, Lehtimäki T, Karhu T, Viinamäki H, Salmi M, Santalahti K, Jalkanen S, Jokelainen J, Keinänen-Kiukaanniemi S, Männikkö M, Herzig K-H, Järvelin M-R, Sebert S, Kettunen J. Genome-wide association study identifies seven novel loci associating with circulating cytokines and cell adhesion molecules in Finns. *J Med Genet* 2019;**56**:607–616.

8. Ahola-Olli AV, Wurtz P, Havulinna AS, Aalto K, Pitkanen N, Lehtimaki T, Kahonen M, Lyytikainen LP, Raitoharju E, Seppala I, Sarin AP, Ripatti S, Palotie A, Perola M, Viikari JS, Jalkanen S, Maksimow M, Salomaa V, Salmi M, Kettunen J, Raitakari OT. Genome-wide Association Study Identifies 27 Loci Influencing Concentrations of Circulating Cytokines and Growth Factors. *Am J Hum Genet* 2017;**100**:40–50.

9. Day FR, Loh PR, Scott RA, Ong KK, Perry JR. A robust example of collider bias in a genetic association study. *Am J Hum Genet* 2016;**98**:392–393.

10. Marchini J, Howie B, Myers S, McVean G, Donnelly P. A new multipoint method for genome-wide association studies by imputation of genotypes. *Nat Genet* 2007;**39**:906–913.

11. Willer CJ, Li Y, Abecasis GR. METAL: fast and efficient meta-analysis of genomewide association scans. *Bioinformatics* 2010;**26**:2190–2191.

12. Nikpay M, Goel A, Won H-H, Hall LM, Willenborg C, Kanoni S, Saleheen D, Kyriakou T, Nelson CP, Hopewell JC, Consortium the Cardi. A comprehensive 1000 Genomes–based genome-wide association meta-analysis of coronary artery disease. *Nat Genet* 2015;**47**:1121.

13. Malik R, Chauhan G, Traylor M, Sargurupremraj M, Okada Y, Mishra A, Rutten-Jacobs L, Giese A-K, Laan SW van der, Gretarsdottir S, Anderson CD, Chong M, Adams HHH, Ago T, Almgren P, Amouyel P, Ay H, Bartz TM, Benavente OR, Bevan S, Boncoraglio GB, Brown RD, Butterworth AS, Carrera C, Carty CL, Chasman DI, Chen W-M, Cole JW, Correa A, Cotlarciuc I, Cruchaga C, Danesh J, Bakker PIW de, DeStefano AL, Hoed M den, Duan Q, Engelter ST, Falcone GJ, Gottesman RF, Grewal RP, Gudnason V, Gustafsson S, Haessler J, Harris TB, Hassan A, Havulinna AS, Heckbert SR, Holliday EG, Howard G, Hsu F-C, Hyacinth HI, Ikram MA, Ingelsson E, Irvin MR, Jian X, Jiménez-Conde J, Johnson JA, Jukema JW, Kanai M, Keene KL, Kissela BM, Kleindorfer DO, Kooperberg C, Kubo M, Lange LA, Langefeld CD, Langenberg C, Launer LJ, Lee J-M, Lemmens R, Leys D, Lewis CM, Lin W-Y, Lindgren AG, Lorentzen E, Magnusson PK, Maguire J, Manichaikul A, McArdle PF, Meschia JF, Mitchell BD, Mosley TH, Nalls MA, Ninomiya T, O'Donnell MJ, Psaty BM, Pulit SL, Rannikmäe K, Reiner AP, Rexrode KM, Rice K, Rich SS, Ridker PM, Rost NS, Rothwell PM, Rotter JI, Rundek T, Sacco RL, Sakaue S, Sale MM, others. Multiancestry genome-wide association study of 520,000 subjects identifies 32 loci associated with stroke and stroke subtypes. *Nature Genetics*.

14. Mahajan A, Taliun D, Thurner M, Robertson NR, Torres JM, Rayner NW, Payne AJ, Steinthorsdottir V, Scott RA, Grarup N, Cook JP, Schmidt EM, Wuttke M, Sarnowski C, Mägi R, Nano J, Gieger C, Trompet S, Lecoeur C, Preuss MH, Prins BP, Guo X, Bielak LF, Below JE, Bowden DW, Chambers JC, Kim YJ, Ng MCY, Petty LE, Sim X, Zhang W, Bennett AJ, Bork-Jensen J, Brummett CM, Canouil M, Ec kardt K-U, Fischer K, Kardia SLR, Kronenberg F, Läll K, Liu C-T, Locke AE, Luan J, Ntalla I, Nylander V, Schönherr S, Schurmann C, Yengo L, Bottinger EP, Brandslund I, Christensen C, Dedoussis G, Florez JC, Ford I, Franco OH, Frayling TM, Giedraitis V, Hackinger S, Hattersley AT, Herder C, Ikram MA, Ingelsson M, Jørgensen ME, Jørgensen T, Kriebel J, Kuusisto J, Ligthart S, Lindgren CM, Linneberg A, Lyssenko V, Mamakou V, Meitinger T, Mohlke KL, Morris AD, Nadkarni G, Pankow JS, Peters A, Sattar N, Stančáková A, Strauch K, Taylor KD, Thorand B, Thorleifsson G, Thorsteinsdottir U, Tuomilehto J, Witte DR, Dupuis J, Peyser PA, Zeggini E, Loos RJF, Froguel P, Ingelsson E, Lind L, Groop L, Laakso M, Collins FS, Jukema JW, Palmer CNA, Grallert H, Metspalu A, Dehghan A, Köttgen A, Abecasis GR, Meigs JB, Rotter JI, Marchini J, Pedersen O, Hansen T,

Langenberg C, Wareham NJ, Stefansson K, Gloyn AL, Morris AP, Boehnke M, McCarthy MI. Fine-mapping type 2 diabetes loci to single-variant resolution using high-density imputation and islet-specific epigenome maps. *Nat Genet* 2018;**50**:1505–1513.

15. Pulit SL, Stoneman C, Morris AP, Wood AR, Glastonbury CA, Tyrrell J, Yengo L, Ferreira T, Marouli E, Ji Y, Yang J, Jones S, Beaumont R, Croteau-Chonka DC, Winkler TW, Consortium G, Hattersley AT, Loos RJF, Hirschhorn JN, Visscher PM, Frayling TM, Yaghootkar H, Lindgren CM. Meta-analysis of genome-wide association studies for body fat distribution in 694 649 individuals of European ancestry. *Hum Mol Genet* 2019;**28**:166–174.

16. Shungin D, Winkler TW, Croteau-Chonka DC, Ferreira T, Locke AE, Mägi R, Strawbridge RJ, Pers TH, Fischer K, Justice AE, Workalemahu T, Wu JMW, Buchkovich ML, Heard-Costa NL, Roman TS, Drong AW, Song C, Gustafsson S, Day FR, Esko T, Fall T, Kutalik Z, Luan J, Randall JC, Scherag A, Vedantam S, Wood AR, Chen J, Fehrmann R, Karjalainen J, Kahali B, Liu C-T, Schmidt EM, Absher D, Amin N, Anderson D, Beekman M, Bragg-Gresham JL, Buyske S, Demirkan A, Ehret GB, Feitosa MF, Goel A, Jackson AU, Johnson T, Kleber ME, Kristiansson K, Mangino M, Mateo Leach I, Medina-Gomez C, Palmer CD, Pasko D, Pechlivanis S, Peters MJ, Prokopenko I, Stančáková A, Ju Sung Y, Tanaka T, Teumer A, Van Vliet-Ostaptchouk JV, Yengo L, Zhang W, Albrecht E, Ärnlöv J, Arscott GM, Bandinelli S, Barrett A, Bellis C, Bennett AJ, Berne C, Blüher M, Böhringer S, Bonnet F, Böttcher Y, Bruinenberg M, Carba DB, Caspersen IH, Clarke R, Warwick Daw E, Deelen J, Deelman E, Delgado G, Doney ASF, Eklund N, Erdos MR, Estrada K, Eury E, Friedrich N, Garcia ME, Giedraitis V, Gigante B, Go AS, Golay A, Grallert H, Grammer TB, Gräßler J, Grewal J, Groves CJ, Haller T, Hallmans G, Mohlke KL. New genetic loci link adipose and insulin biology to body fat distribution. *Nature* 2015;**518**:187.

17. Carter AR, Gill D, Davies NM, Taylor AE, Tillmann T, Vaucher J, Wootton RE, Munafo MR, Hemani G, Malik R, Seshadri S, Woo D, Burgess S, Davey Smith G, Holmes MV, Tzoulaki I, Howe LD, Dehghan A. Understanding the consequences of education inequality on cardiovascular disease: mendelian randomisation study. *BMJ* 2019;**365**:l1855.

18. The Neale Lab. Updated GWAS Analysis of the UK Biobankhttp://www.nealelab.is/uk-biobank (3 February 2021)

19. Dupuis J, Langenberg C, Prokopenko I, Saxena R, Soranzo N, Jackson AU, Wheeler E, Glazer NL, Bouatia-Naji N, Gloyn AL, Lindgren CM, Mägi R, Morris AP, Randall J, Johnson T, Elliott P, Rybin D, Thorleifsson G, Steinthorsdottir V, Henneman P, Grallert H, Dehghan A, Hottenga JJ, Franklin CS, Navarro P, Song K, Goel A, Perry JRB, Egan JM, Lajunen T, Grarup N, Sparsø T, Doney A, Voight BF, Stringham HM, Li M, Kanoni S, Shrader P, Cavalcanti-Proença C, Kumari M, Qi L, Timpson NJ, Gieger C, Zabena C, Rocheleau G, Ingelsson E, An P, O'Connell J, Luan J, Elliott A, McCarroll SA, Payne F, Roccasecca RM, Pattou F, Sethupathy P,

Ardlie K, Ariyurek Y, Balkau B, Barter P, Beilby JP, Ben-Shlomo Y, Benediktsson R, Bennett AJ, Bergmann S, Bochud M, Boerwinkle E, Bonnefond A, Bonnycastle LL, Borch-Johnsen K, Böttcher Y, Brunner E, Bumpstead SJ, Charpentier G, Chen Y-DI, Chines P, Clarke R, Coin LJM, Cooper MN, Cornelis M, Crawford G, Crisponi L, Day INM, Geus EJC de, Delplanque J, Dina C, Erdos MR, Fedson AC, Fischer-Rosinsky A, Forouhi NG, Fox CS, Frants R, Franzosi MG, Galan P, Goodarzi MO, Graessler J, Groves CJ, Grundy S, Gwilliam R, Gyllensten U, Hadjadj S, Hallmans G, Hammond N, Han X, Hartikainen A-L, Hassanali N, Hayward C, Heath SC, Hercberg S, Herder C, Hicks AA, Hillman DR, Hingorani AD, Hofman A, Hui J, Hung J, Isomaa B, Johnson PRV, Jørgensen T, Jula A, Kaakinen M, Kaprio J, Kesaniemi YA, Kivimaki M, Knight B, Koskinen S, Kovacs P, Kyvik KO, Lathrop GM, Lawlor DA, Le Bacquer O, Lecoeur C, Li Y, Lyssenko V, Mahley R, Mangino M, Manning AK, Martínez-Larrad MT, McAteer JB, McCulloch LJ, McPherson R, Meisinger C, Melzer D, Meyre D, Mitchell BD, Morken MA, Mukherjee S, Naitza S, Narisu N, Neville MJ, Oostra BA, Orrù M, Pakyz R, Palmer CNA, Paolisso G, Pattaro C, Pearson D, Peden JF, Pedersen NL, Perola M, Pfeiffer AFH, Pichler I, Polasek O, Posthuma D, Potter SC, Pouta A, Province MA, Psaty BM, Rathmann W, Rayner NW, Rice K, Ripatti S, Rivadeneira F, Roden M, Rolandsson O, Sandbaek A, Sandhu M, Sanna S, Sayer AA, Scheet P, Scott LJ, Seedorf U, Sharp SJ, Shields B, Sigurðsson G, Sijbrands EJG, Silveira A, Simpson L, Singleton A, Smith NL, Sovio U, Swift A, Syddall H, Syvänen A-C, Tanaka T, Thorand B, Tichet J, Tönjes A, Tuomi T, Uitterlinden AG, Dijk KW van, Hoek M van, Varma D, Visvikis-Siest S, Vitart V, Vogelzangs N, Waeber G, Wagner PJ, Walley A, Walters GB, Ward KL, Watkins H, Weedon MN, Wild SH, Willemsen G, Witteman JCM, Yarnell JWG, Zeggini E, Zelenika D, Zethelius B, Zhai G, Zhao JH, Zillikens MC, Borecki IB, Loos RJF, Meneton P, Magnusson PKE, Nathan DM, Williams GH, Hattersley AT, Silander K, Salomaa V, Smith GD, Bornstein SR, Schwarz P, Spranger J, Karpe F, Shuldiner AR, Cooper C, Dedoussis GV, Serrano-Ríos M, Morris AD, Lind L, Palmer LJ, Hu FB, Franks PW, Ebrahim S, Marmot M, Kao WHL, Pankow JS, Sampson MJ, Kuusisto J, Laakso M, Hansen T, Pedersen O, Pramstaller PP, Wichmann HE, Illig T, Rudan I, Wright AF, Stumvoll M, Campbell H, Wilson JF, Bergman RN, Buchanan TA, Collins FS, Mohlke KL, Tuomilehto J, Valle TT, Altshuler D, Rotter JI, Siscovick DS, Penninx BWJH, Boomsma DI, Deloukas P, Spector TD, Frayling TM, Ferrucci L, Kong A, Thorsteinsdottir U, Stefansson K, Duijn CM van, Aulchenko YS, Cao A, Scuteri A, Schlessinger D, Uda M, Ruokonen A, Jarvelin M-R, Waterworth DM, Vollenweider P, Peltonen L, Mooser V, Abecasis GR, Wareham NJ, Sladek R, DIAGRAM Consortium, GIANT Consortium, Global BPgen Consortium, Anders Hamsten on behalf of Procardis Consortium, the MAGIC investigators. New genetic loci implicated in fasting glucose homeostasis and their impact on type 2 diabetes risk. *Nat Genet* 2010;**42**:105–116.

20. Graham SE, Clarke SL, Wu K-HH, Kanoni S, Zajac GJM, Ramdas S, Surakka I, Ntalla I, Vedantam S, Winkler TW, Locke AE, Marouli E, Hwang MY, Han S, Narita A, Choudhury A, Bentley AR, Ekoru K, Verma A, Trivedi B, Martin HC, Hunt KA, Hui Q, Klarin D, Zhu X, Thorleifsson G, Helgadottir A, Gudbjartsson DF, Holm H, Olafsson I, Akiyama M, Sakaue S, Terao C, Kanai M, Zhou W,

Brumpton BM, Rasheed H, Ruotsalainen SE, Havulinna AS, Veturi Y, Feng Q, Rosenthal EA, Lingren T, Pacheco JA, Pendergrass SA, Haessler J, Giulianini F, Bradford Y, Miller JE, Campbell A, Lin K, Millwood IY, Hindy G, Rasheed A, Faul JD, Zhao W, Weir DR, Turman C, Huang H, Graff M, Mahajan A, Brown MR, Zhang W, Yu K, Schmidt EM, Pandit A, Gustafsson S, Yin X, Luan J, Zhao J-H, Matsuda F, Jang H-M, Yoon K, Medina-Gomez C, Pitsillides A, Hottenga JJ, Willemsen G, Wood AR, Ji Y, Gao Z, Haworth S, Mitchell RE, Chai JF, Aadahl M, Yao J, Manichaikul A, Warren HR, Ramirez J, Bork-Jensen J, Kårhus LL, Goel A, Sabater-Lleal M, Noordam R, Sidore C, Fiorillo E, McDaid AF, Marques-Vidal P, Wielscher M, Trompet S, Sattar N, Møllehave LT, Thuesen BH, Munz M, Zeng L, Huang J, Yang B, Poveda A, Kurbasic A, Lamina C, Forer L, Scholz M, Galesloot TE, Bradfield JP, Daw EW, Zmuda JM, Mitchell JS, Fuchsberger C, Christensen H, Brody JA, Feitosa MF, Wojczynski MK, Preuss M, Mangino M, Christofidou P, Verweij N, Benjamins JW, Engmann J, Kember RL, Slieker RC, Lo KS, Zilhao NR, Le P, Kleber ME, Delgado GE, Huo S, Ikeda DD, Iha H, Yang J, Liu J, Leonard HL, Marten J, Schmidt B, Arendt M, Smyth LJ, Cañadas-Garre M, Wang C, Nakatochi M, Wong A, Hutri-Kähönen N, Sim X, Xia R, Huerta-Chagoya A, Fernandez-Lopez JC, Lyssenko V, Ahmed M, Jackson AU, Irvin MR, Oldmeadow C, Kim H-N, Ryu S, Timmers PRHJ, Arbeeva L, Dorajoo R, Lange LA, Chai X, Prasad G, Lorés-Motta L, Pauper M, Long J, Li X, Theusch E, Takeuchi F, Spracklen CN, Loukola A, Bollepalli S, Warner SC, Wang YX, Wei WB, Nutile T, Ruggiero D, Sung YJ, Hung Y-J, Chen S, Liu F, Yang J, Kentistou KA, Gorski M, Brumat M, Meidtner K, Bielak LF, Smith JA, Hebbar P, Farmaki A-E, Hofer E, Lin M, Xue C, Zhang J, Concas MP, Vaccargiu S, Most PJ van der, Pitkänen N, Cade BE, Lee J, Laan SW van der, Chitrala KN, Weiss S, Zimmermann ME, Lee JY, Choi HS, Nethander M, Freitag-Wolf S, Southam L, Rayner NW, Wang CA, Lin S-Y, Wang J-S, Couture C, Lyytikäinen L-P, Nikus K, Cuellar-Partida G, Vestergaard H, Hildalgo B, Giannakopoulou O, Cai Q, Obura MO, Setten J van, Li X, Schwander K, Terzikhan N, Shin JH, Jackson RD, Reiner AP, Martin LW, Chen Z, Li L, Highland HM, Young KL, Kawaguchi T, Thiery J, Bis JC, Nadkarni GN, Launer LJ, Li H, Nalls MA, Raitakari OT, Ichihara S, Wild SH, Nelson CP, Campbell H, Jäger S, Nabika T, Al-Mulla F, Niinikoski H, Braund PS, Kolcic I, Kovacs P, Giardoglou T, Katsuya T, Bhatti KF, Kleijn D de, Borst GJ de, Kim EK, Adams HHH, Ikram MA, Zhu X, Asselbergs FW, Kraaijeveld AO, Beulens JWJ, Shu X-O, Rallidis LS, Pedersen O, Hansen T, Mitchell P, Hewitt AW, Kähönen M, Pérusse L, Bouchard C, Tönjes A, Chen Y-DI, Pennell CE, Mori TA, Lieb W, Franke A, Ohlsson C, Mellström D, Cho YS, Lee H, Yuan J-M, Koh W-P, Rhee SY, Woo J-T, Heid IM, Stark KJ, Völzke H, Homuth G, Evans MK, Zonderman AB, Polasek O, Pasterkamp G, Hoefer IE. The power of genetic diversity in genome-wide association studies of lipids. *Nature* 2021;**600**:675–679.

21. Wootton RE, Richmond RC, Stuijfzand BG, Lawn RB, Sallis HM, Taylor GMJ, Hemani G, Jones HJ, Zammit S, Davey Smith G, Munafo MR. Evidence for causal effects of lifetime smoking on risk for depression and schizophrenia: a Mendelian randomisation study. *Psychol Med* 2019:1–9.

22. Wheeler E, Leong A, Liu C-T, Hivert M-F, Strawbridge RJ, Podmore C, Li M, Yao J, Sim X, Hong J, Chu AY, Zhang W, Wang X, Chen P, Maruthur NM, Porneala BC, Sharp SJ, Jia Y, Kabagambe EK, Chang L-C, Chen W-M, Elks CE, Evans DS, Fan Q, Giulianini F, Go MJ, Hottenga J-J, Hu Y, Jackson AU, Kanoni S, Kim YJ, Kleber ME, Ladenvall C, Lecoeur C, Lim S-H, Lu Y, Mahajan A, Marzi C, Nalls MA, Navarro P, Nolte IM, Rose LM, Rybin DV, Sanna S, Shi Y, Stram DO, Takeuchi F, Tan SP, Most PJ van der, Van Vliet-Ostaptchouk JV, Wong A, Yengo L, Zhao W, Goel A, Martinez Larrad MT, Radke D, Salo P, Tanaka T, Iperen EPA van, Abecasis G, Afaq S, Alizadeh BZ, Bertoni AG, Bonnefond A, Böttcher Y, Bottinger EP, Campbell H, Carlson OD, Chen C-H, Cho YS, Garvey WT, Gieger C, Goodarzi MO, Grallert H, Hamsten A, Hartman CA, Herder C, Hsiung CA, Huang J, Igase M, Isono M, Katsuya T, Khor C-C, Kiess W, Kohara K, Kovacs P, Lee J, Lee W-J, Lehne B, Li H, Liu J, Lobbens S, Luan J, Lyssenko V, Meitinger T, Miki T, Miljkovic I, Moon S, Mulas A, Müller G, Müller-Nurasyid M, Nagaraja R, Nauck M, Pankow JS, Polasek O, Prokopenko I, Ramos PS, Rasmussen-Torvik L, Rathmann W, Rich SS, Robertson NR, Roden M, Roussel R, Rudan I, Scott RA, Scott WR, Sennblad B, Siscovick DS, Strauch K, Sun L, Swertz M, Tajuddin SM, Taylor KD, Teo Y-Y, Tham YC, Tönjes A, Wareham NJ, Willemsen G, Wilsgaard T, Hingorani AD, EPIC-CVD Consortium, EPIC-InterAct Consortium, Lifelines Cohort Study, Egan J, Ferrucci L, Hovingh GK, Jula A, Kivimaki M, Kumari M, Njølstad I, Palmer CNA, Serrano Ríos M, Stumvoll M, Watkins H, Aung T, Blüher M, Boehnke M, Boomsma DI, Bornstein SR, Chambers JC, Chasman DI, Chen Y-DI, Chen Y-T, Cheng C-Y, Cucca F, Geus EJC de, Deloukas P, Evans MK, Fornage M, Friedlander Y, Froguel P, Groop L, Gross MD, Harris TB, Hayward C, Heng C-K, Ingelsson E, Kato N, Kim B-J, Koh W-P, Kooner JS, Körner A, Kuh D, Kuusisto J, Laakso M, Lin X, Liu Y, Loos RJF, Magnusson PKE, März W, McCarthy MI, Oldehinkel AJ, Ong KK, Pedersen NL, Pereira MA, Peters A, Ridker PM, Sabanayagam C, Sale M, Saleheen D, Saltevo J, Schwarz PEH, Sheu WHH, Snieder H, Spector TD, Tabara Y, Tuomilehto J, Dam RM van, Wilson JG, Wilson JF, Wolffenbuttel BHR, Wong TY, Wu J-Y, Yuan J-M, Zonderman AB, Soranzo N, Guo X, Roberts DJ, Florez JC, Sladek R, Dupuis J, Morris AP, Tai E-S, Selvin E, Rotter JI, Langenberg C, Barroso I, Meigs JB. Impact of common genetic determinants of Hemoglobin A1c on type 2 diabetes risk and diagnosis in ancestrally diverse populations: A transethnic genome-wide meta-analysis. Gregg E, ed. *PLOS Med* 2017;**14**:e1002383.

23. Hemani G, Zheng J, Elsworth B, Wade KH, Haberland V, Baird D, Laurin C, Burgess S, Bowden J, Langdon R, Tan VY, Yarmolinsky J, Shihab HA, Timpson NJ, Evans DM, Relton C, Martin RM, Davey Smith G, Gaunt TR, Haycock PC. The MR-Base platform supports systematic causal inference across the human phenome. *eLife* 2018;**7**.

24. Sun BB, Maranville JC, Peters JE, Stacey D, Staley JR, Blackshaw J, Burgess S, Jiang T, Paige E, Surendran P, Oliver-Williams C, Kamat MA, Prins BP, Wilcox SK, Zimmerman ES, Chi A, Bansal N, Spain SL, Wood AM, Morrell NW, Bradley JR, Janjic N, Roberts DJ, Ouwehand WH, Todd JA, Soranzo N, Suhre K, Paul DS,

Fox CS, Plenge RM, Danesh J, Runz H, Butterworth AS. Genomic atlas of the human plasma proteome. *Nature* 2018;**558**:73–79.

25. Folkersen L, Gustafsson S, Wang Q, Hansen DH, Hedman ÅK, Schork A, Page K, Zhernakova DV, Wu Y, Peters J, Eriksson N, Bergen SE, Boutin TS, Bretherick AD, Enroth S, Kalnapenkis A, Gådin JR, Suur BE, Chen Y, Matic L, Gale JD, Lee J, Zhang W, Quazi A, Ala-Korpela M, Choi SH, Claringbould A, Danesh J, Davey Smith G, Masi F de, Elmståhl S, Engström G, Fauman E, Fernandez C, Franke L, Franks PW, Giedraitis V, Haley C, Hamsten A, Ingason A, Johansson Å, Joshi PK, Lind L, Lindgren CM, Lubitz S, Palmer T, Macdonald-Dunlop E, Magnusson M, Melander O, Michaelsson K, Morris AP, Mägi R, Nagle MW, Nilsson PM, Nilsson J, Orho-Melander M, Polasek O, Prins B, Pålsson E, Qi T, Sjögren M, Sundström J, Surendran P, Võsa U, Werge T, Wernersson R, Westra H-J, Yang J, Zhernakova A, Ärnlöv J, Fu J, Smith JG, Esko T, Hayward C, Gyllensten U, Landen M, Siegbahn A, Wilson JF, Wallentin L, Butterworth AS, Holmes MV, Ingelsson E, Mälarstig A. Genomic and drug target evaluation of 90 cardiovascular proteins in 30,931 individuals. *Nat Metab* 2020;**2**:1135–1148.

26. The GTEx Consortium. The GTEx Consortium atlas of genetic regulatory effects across human tissues. *Science* 2020;**369**:1318.

27. Võsa U, Claringbould A, Westra H-J, Bonder MJ, Deelen P, Zeng B, Kirsten H, Saha A, Kreuzhuber R, Yazar S, Brugge H, Oelen R, Vries DH de, Wijst MGP van der, Kasela S, Pervjakova N, Alves I, Favé M-J, Agbessi M, Christiansen MW, Jansen R, Seppälä I, Tong L, Teumer A, Schramm K, Hemani G, Verlouw J, Yaghootkar H, Sönmez Flitman R, Brown A, Kukushkina V, Kalnapenkis A, Rüeger S, Porcu E, Kronberg J, Kettunen J, Lee B, Zhang F, Qi T, Hernandez JA, Arindrarto W, Beutner F, BIOS Consortium, Hoen PAC ’t, Meurs J van, Dongen J van, Iterson M van, Swertz MA, i2QTL Consortium, Jan Bonder M, Dmitrieva J, Elansary M, Fairfax BP, Georges M, Heijmans BT, Hewitt AW, Kähönen M, Kim Y, Knight JC, Kovacs P, Krohn K, Li S, Loeffler M, Marigorta UM, Mei H, Momozawa Y, Müller-Nurasyid M, Nauck M, Nivard MG, Penninx BWJH, Pritchard JK, Raitakari OT, Rotzschke O, Slagboom EP, Stehouwer CDA, Stumvoll M, Sullivan P, Hoen PAC ’t, Thiery J, Tönjes A, Dongen J van, Iterson M van, Veldink JH, Völker U, Warmerdam R, Wijmenga C, Swertz M, Andiappan A, Montgomery GW, Ripatti S, Perola M, Kutalik Z, Dermitzakis E, Bergmann S, Frayling T, Meurs J van, Prokisch H, Ahsan H, Pierce BL, Lehtimäki T, Boomsma DI, Psaty BM, Gharib SA, Awadalla P, Milani L, Ouwehand WH, Downes K, Stegle O, Battle A, Visscher PM, Yang J, Scholz M, Powell J, Gibson G, Esko T, Franke L. Large-scale cis- and trans-eQTL analyses identify thousands of genetic loci and polygenic scores that regulate blood gene expression. *Nat Genet* 2021;**53**:1300–1310.

28. Kent WJ, Sugnet CW, Furey TS, Roskin KM, Pringle TH, Zahler AM, Haussler D. The human genome browser at UCSC. *Genome Res* 2002;**12**:996–1006.

29. Palmer TM, Lawlor DA, Harbord RM, Sheehan NA, Tobias JH, Timpson NJ, Davey Smith G, Sterne JA. Using multiple genetic variants as instrumental variables for modifiable risk factors. *Stat Methods Med Res* 2012;**21**:223–242.

30. Burgess S, Thompson SG. Avoiding bias from weak instruments in Mendelian randomization studies. *Int J Epidemiol* 2011;**40**:755–764.

31. Burgess S, Davies NM, Thompson SG. Bias due to participant overlap in two-sample Mendelian randomization. *Genet Epidemiol* 2016;**40**:597–608.

32. Burgess S, Butterworth A, Thompson SG. Mendelian Randomization Analysis With Multiple Genetic Variants Using Summarized Data. *Genet Epidemiol* 2013;**37**:658–665.

33. Burgess S, Bowden J, Fall T, Ingelsson E, Thompson SG. Sensitivity Analyses for Robust Causal Inference from Mendelian Randomization Analyses with Multiple Genetic Variants. *Epidemiology* 2017;**28**:30–42.

34. Bowden J, Davey Smith G, Haycock PC, Burgess S. Consistent Estimation in Mendelian Randomization with Some Invalid Instruments Using a Weighted Median Estimator. *Genet Epidemiol* 2016;**40**:304–314.

35. Verbanck M, Chen CY, Neale B, Do R. Detection of widespread horizontal pleiotropy in causal relationships inferred from Mendelian randomization between complex traits and diseases. *Nat Genet* 2018;**50**:693–698.

36. Bowden J, Davey Smith G, Burgess S. Mendelian randomization with invalid instruments: effect estimation and bias detection through Egger regression. *Int J Epidemiol* 2015;**44**:512–525.

37. Burgess S, Thompson SG. Interpreting findings from Mendelian randomization using the MR-Egger method. *Eur J Epidemiol* 2017;**32**:377–389.

38. Giambartolomei C, Vukcevic D, Schadt EE, Franke L, Hingorani AD, Wallace C, Plagnol V. Bayesian Test for Colocalisation between Pairs of Genetic Association Studies Using Summary Statistics. *PLOS Genet* 2014;**10**:1–15.

39. Wakefield J. Bayes factors for genome-wide association studies: comparison with *P*-values. *Genet Epidemiol* 2009;**33**:79–86.

40. Zuber V, Grinberg NF, Gill D, Manipur I, Slob EAW, Patel A, Wallace C, Burgess S. Combining evidence from Mendelian randomization and colocalization: Review and comparison of approaches. *Am J Hum Genet* 2022;**109**:767–782.

41. Raghavan UN, Albert R, Kumara S. Near linear time algorithm to detect community structures in large-scale networks. *Phys Rev E* 2007;**76**:036106.

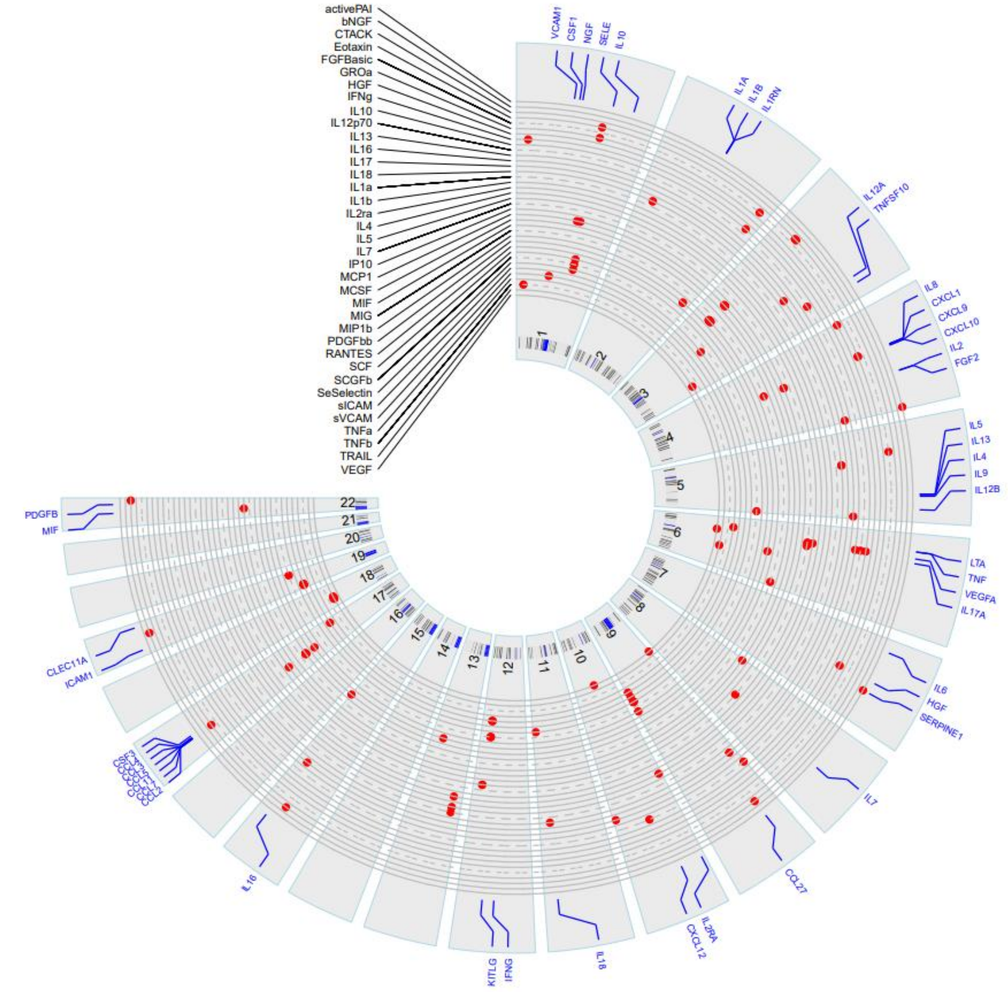

Supplementary Figure 1. Circos plot for the cytokine genome-wide association study results in the Finnish cohorts.

The red points indicate a variant-cytokine association of either $p<5\times10^{-8}$ anywhere in the genome, or $p<10^{-4}$ at the coding gene, pruned at ±500kb window. Cytokines with no such variants are omitted. The coding genes of the considered cytokines are given in the outer circle (see also Supplementary Table 3).

Karhunen V, *et al. BMJMED* 2023; 2:e000157. doi: 10.1136/bmjmed-2022-000157

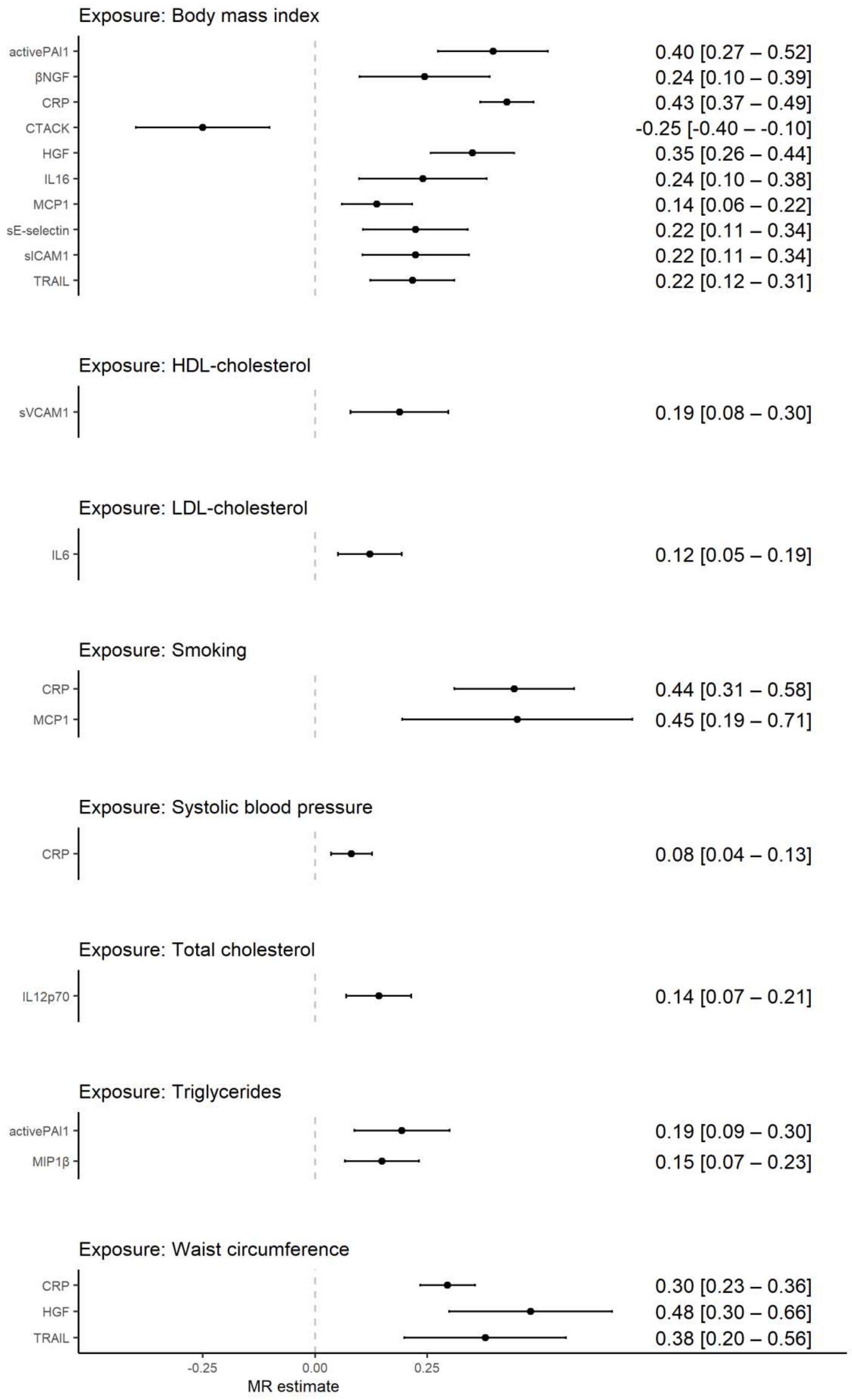

Karhunen V, *et al. BMJMED* 2023; 2:e000157. doi: 10.1136/bmjmed-2022-000157

Supplementary Figure 2. Mendelian Randomization results (point estimates and their 95% confidence intervals) for the effect of genetically predicted cardiometabolic traits on circulating cytokine levels.

The results (given as effect size estimate [95% confidence interval]) are plotted only for effects with $P < 0.0011$ (0.05/47 cytokines) and are per 1-standard-deviation increase in genetically proxied exposure. HDL: high-density lipoprotein; LDL: low-density lipoprotein; CRP: C-reactive protein. The abbreviations for the cytokines are given in Supplementary Table 1.

Karhunen V, *et al. BMJMED* 2023; 2:e000157. doi: 10.1136/bmjmed-2022-000157

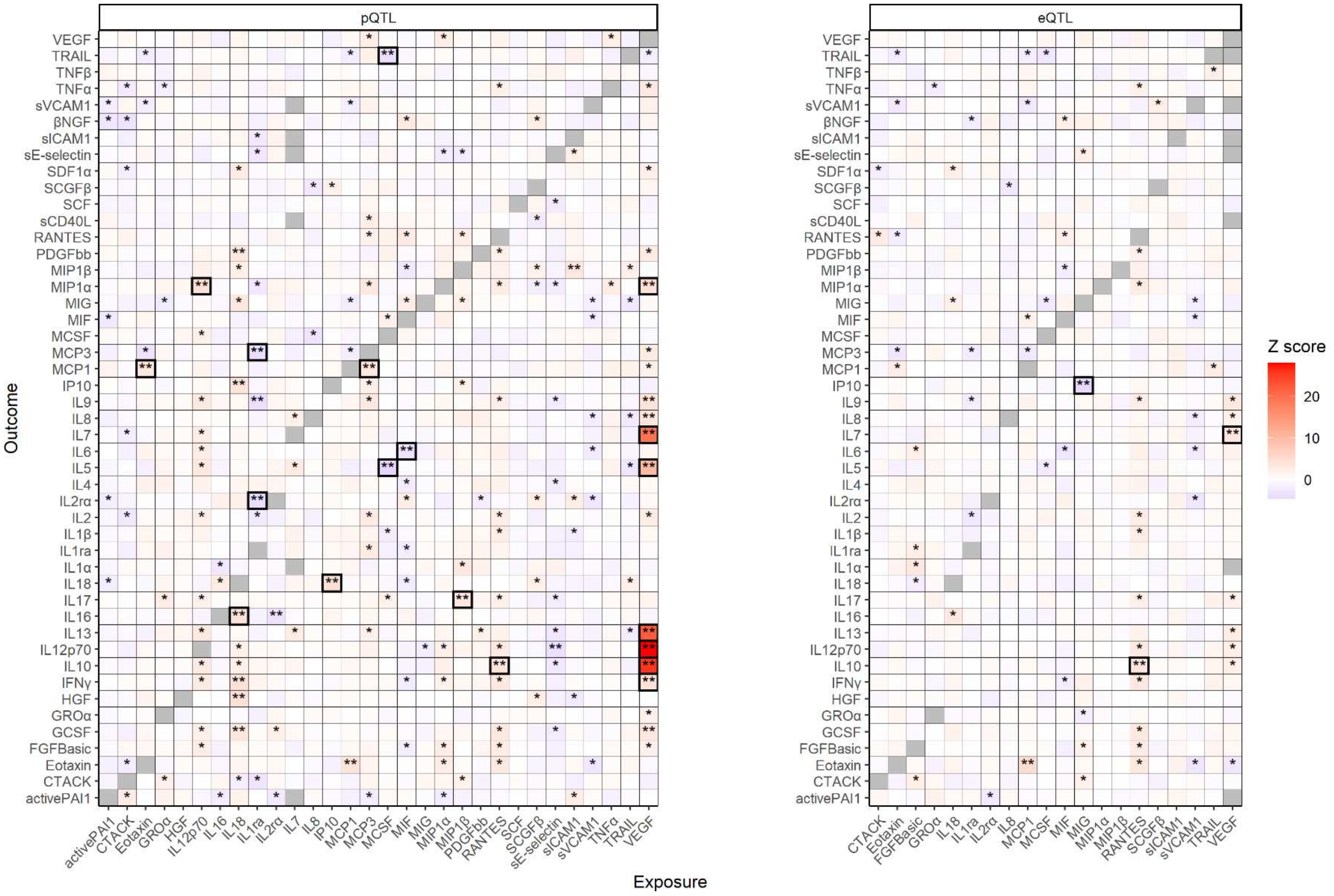

Supplementary Figure 3. Mendelian randomization estimates for the effects of genetically predicted cytokine levels on other circulating cytokines when considering cis-pQTL (left panel) and cis-eQTL (right panel) instruments.

Associations with P < 0.05 are denoted with a single asterisk, and associations with P<0.0011 (0.05/number of cytokines) are denoted with a double asterisk. Those associations with additional colocalization evidence ($PP_{shared} + PP_{distinct} > 0.5$ and $PP_{shared} / (PP_{shared} + PP_{distinct}) > 0.5$) are highlighted with a box. PP = posterior probability.

Karhunen V, *et al. BMJMED* 2023; 2:e000157. doi: 10.1136/bmjmed-2022-000157

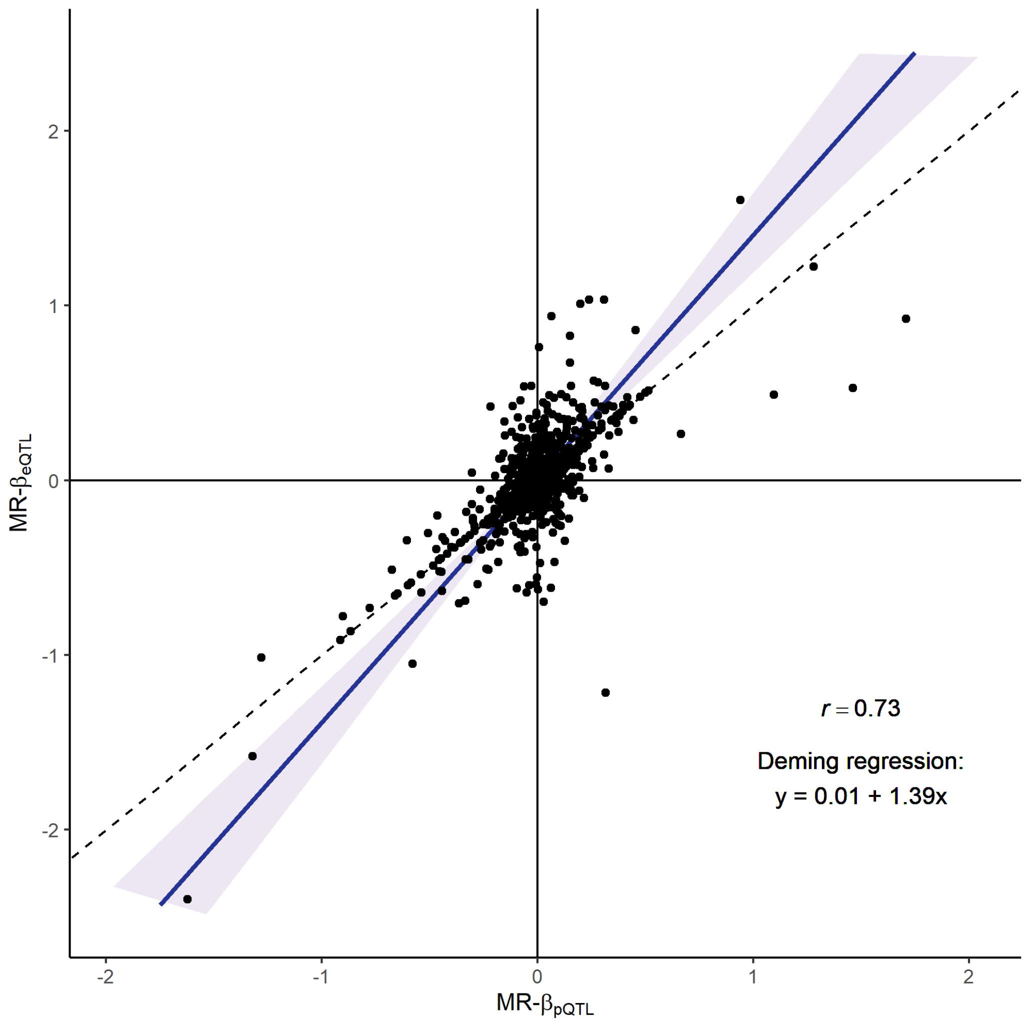

Supplementary Figure 4. Scatter plot comparing Mendelian randomization (MR) estimates for the effects of genetically predicted cytokine levels on other circulating cytokine levels when using the two different instrument selection criteria.
The correlation between the MR estimates was quantified using Pearson's coefficient. x-axis: MR effect estimates using *cis*-protein quantitative trait loci instrument selection; y-axis: MR effect estimates using *cis*-expression quantitative trait loci instrument selection. The dashed line is the line of equality, y=x. The blue line is the Deming regression line and the shaded area its 95% confidence band. r: correlation coefficient.

Supplementary Figures 5a-t. Colocalization plots for circulating cytokine levels.

Cytokine-cytokine pairs with evidence for colocalization (PP_{shared} + $PP_{distinct}$ > 0.5 and PP_{shared} /(PP_{shared} + $PP_{distinct}$) > 0.5) within ±500kb of the coding gene of the exposure cytokine. pQTL=protein quantitative trait loci. R^2 = linkage disequilibrium correlation based on the 1000Genomes European reference panel. PP = posterior probability. Available at: https://doi.org/10.5281/zenodo.7215468.

Karhunen V, *et al. BMJMED* 2023; 2:e000157. doi: 10.1136/bmjmed-2022-000157

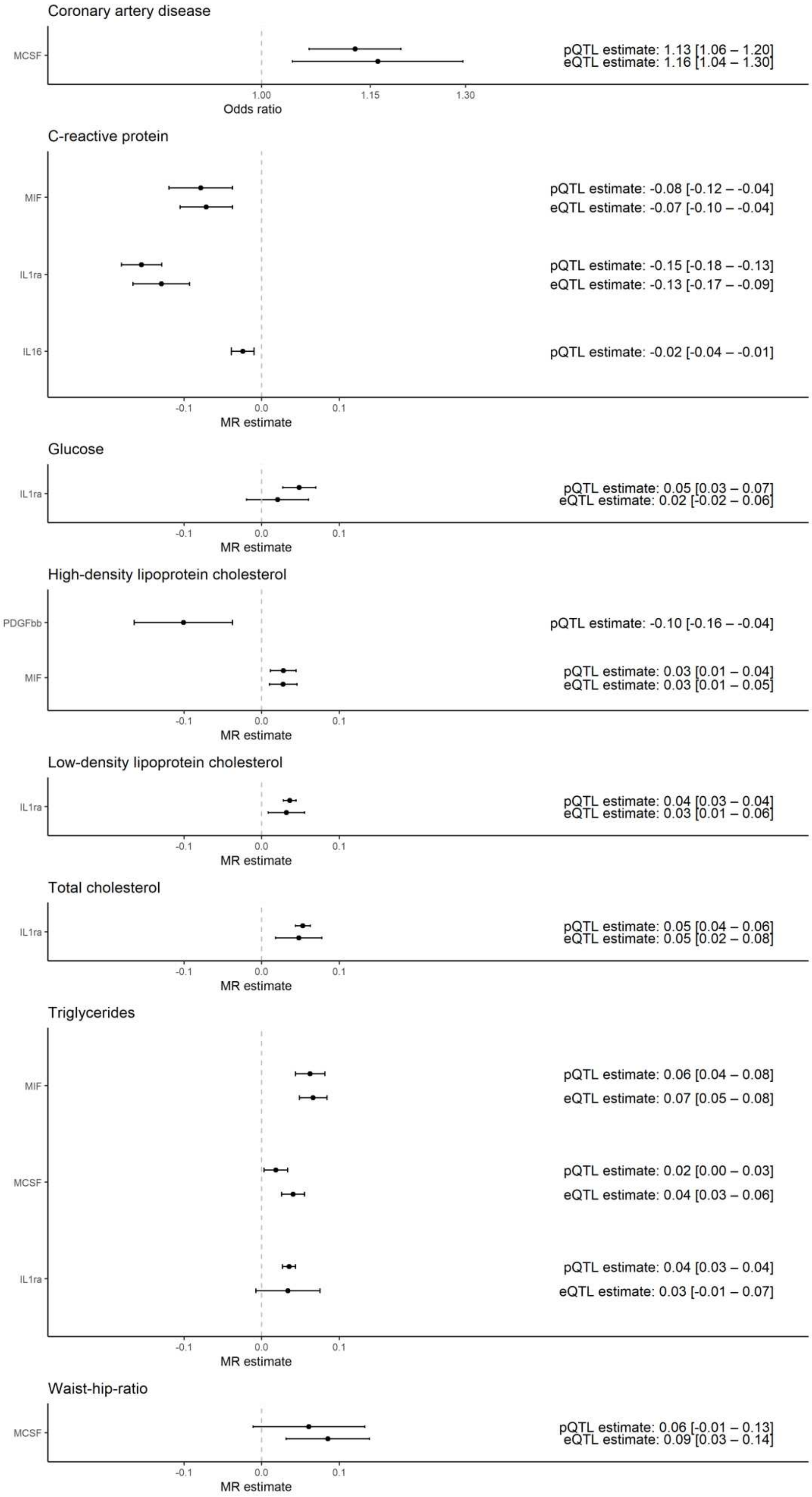
Coronary artery disease
MCSF
pQTL estimate: 1.13 [1.06 – 1.20]
eQTL estimate: 1.16 [1.04 – 1.30]
1.00
1.15
1.30
Odds ratio
C-reactive protein
MIF
pQTL estimate: -0.08 [-0.12 – -0.04]
eQTL estimate: -0.07 [-0.10 – -0.04]
IL1ra
pQTL estimate: -0.15 [-0.18 – -0.13]
eQTL estimate: -0.13 [-0.17 – -0.09]
IL16
pQTL estimate: -0.02 [-0.04 – -0.01]
-0.1
0.0
0.1
MR estimate
Glucose
IL1ra
pQTL estimate: 0.05 [0.03 – 0.07]
eQTL estimate: 0.02 [-0.02 – 0.06]
MR estimate
High-density lipoprotein cholesterol
PDGFbb
pQTL estimate: -0.10 [-0.16 – -0.04]
MIF
pQTL estimate: 0.03 [0.01 – 0.04]
eQTL estimate: 0.03 [0.01 – 0.05]
MR estimate
Low-density lipoprotein cholesterol
IL1ra
pQTL estimate: 0.04 [0.03 – 0.04]
eQTL estimate: 0.03 [0.01 – 0.06]
MR estimate
Total cholesterol
IL1ra
pQTL estimate: 0.05 [0.04 – 0.06]
eQTL estimate: 0.05 [0.02 – 0.08]
MR estimate
Triglycerides
MIF
pQTL estimate: 0.06 [0.04 – 0.08]
eQTL estimate: 0.07 [0.05 – 0.08]
MCSF
pQTL estimate: 0.02 [0.00 – 0.03]
eQTL estimate: 0.04 [0.03 – 0.06]
IL1ra
pQTL estimate: 0.04 [0.03 – 0.04]
eQTL estimate: 0.03 [-0.01 – 0.07]
MR estimate
Waist-hip-ratio
MCSF
pQTL estimate: 0.06 [-0.01 – 0.13]
eQTL estimate: 0.09 [0.03 – 0.14]
MR estimate

30

Supplementary Figure 6. Mendelian Randomization (MR) results (point estimates and their 95% confidence intervals) for the effect of genetically predicted circulating cytokine levels on cardiometabolic phenotypes considered as outcomes.
The x-axis is in standard deviation scale (for quantitative outcomes) or on a log-odds-ratio scale (for binary outcomes). The effect size estimates are per 1-standard-deviation increase in the exposure. The results are plotted only for effects with strong evidence for causality (see Supplementary Methods). pQTL: protein quantitative trait loci; eQTL: expression quantitative trait loci. The abbreviations for cytokines are given in Supplementary Table 1.

Karhunen V, *et al. BMJMED* 2023; 2:e000157. doi: 10.1136/bmjmed-2022-000157

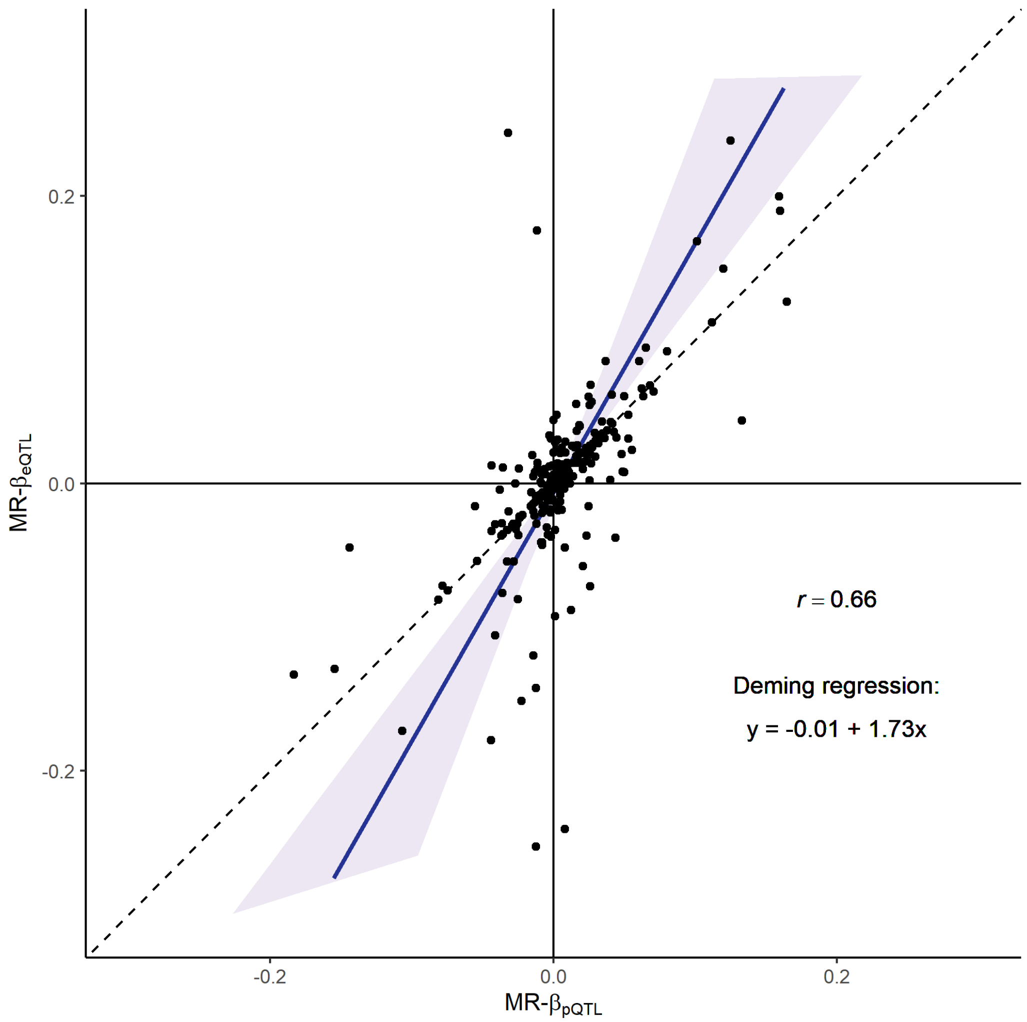

Supplementary Figure 7. Scatter plot comparing Mendelian randomization (MR) estimates for the effects of genetically predicted cytokine levels on cardiometabolic phenotypes considered as outcomes when using the two different instrument selection criteria.

The correlation between the MR estimates was quantified using Pearson's coefficient. x-axis: MR effect estimates using *cis*-protein quantitative trait loci instrument selection; y-axis: MR effect estimates using *cis*-expression quantitative trait loci instrument selection. The dashed line is the line of equality, y=x. The blue line is the Deming regression line and the shaded area its 95% confidence band. r: correlation coefficient.

Karhunen V, *et al. BMJMED* 2023; 2:e000157. doi: 10.1136/bmjmed-2022-000157

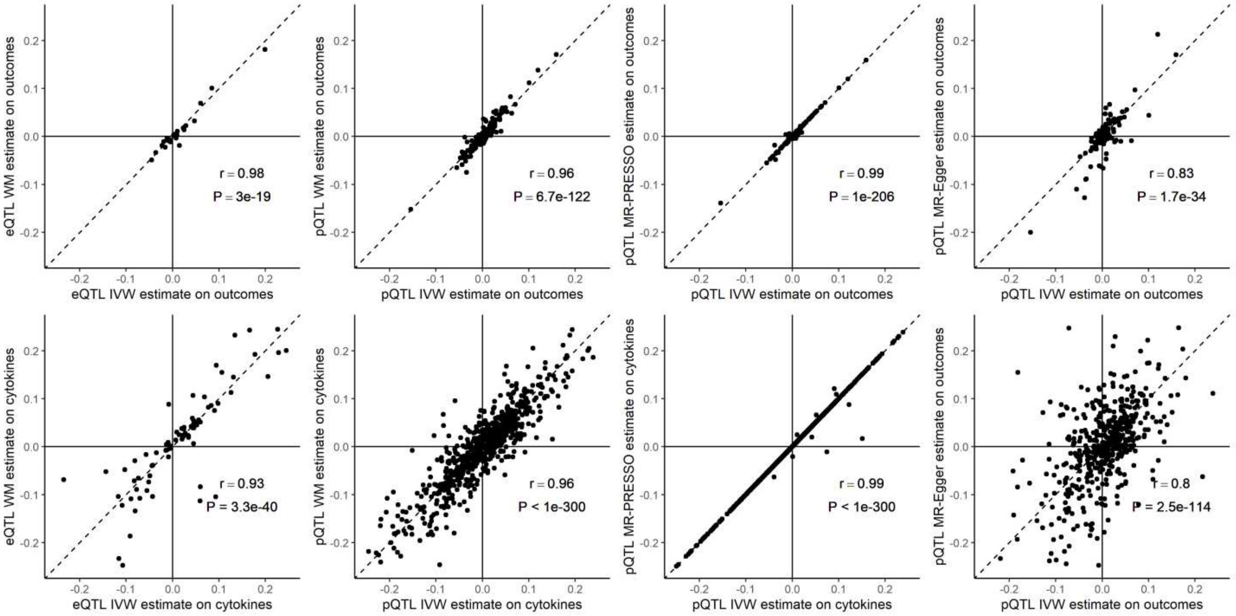

Supplementary Figure 8. Scatter plots comparing Mendelian randomization (MR) estimates for the effects of genetically predicted cytokine levels on circulating cytokine levels and cardiometabolic phenotypes considered as outcomes when using different MR methods.

The correlation between the MR estimates was quantified using Pearson's correlation coefficient. IVW = inverse-variance weighted method; WM = weighted median method; eQTL = expression quantitative trait loci; pQTL = protein quantitative trait loci. r: correlation coefficient.

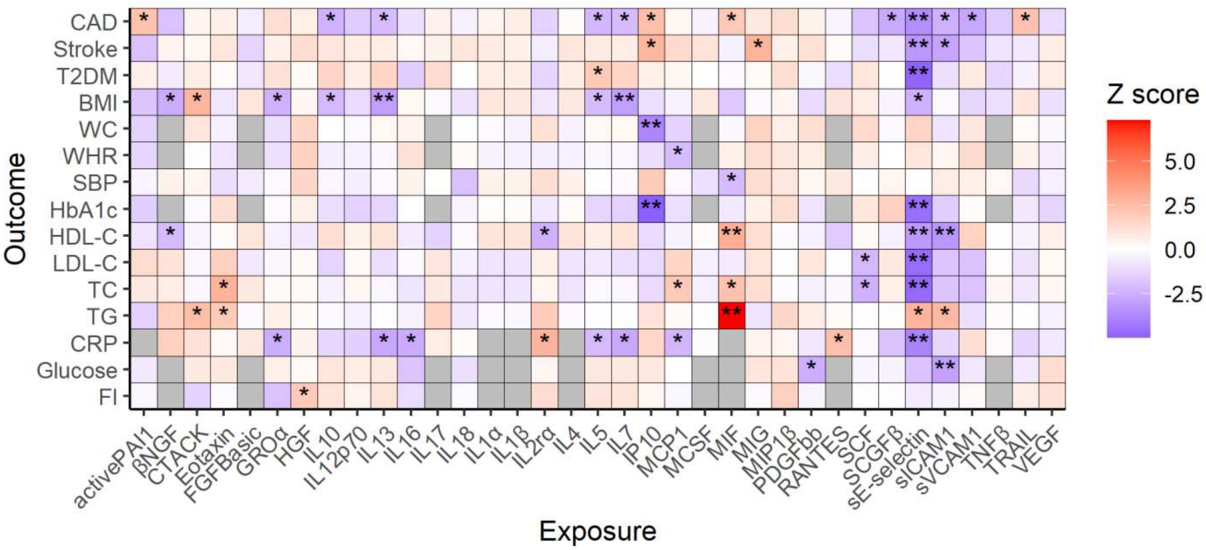

Supplementary Figure 9. Mendelian randomization estimates for the effects of genetically predicted cytokine levels on cardiometabolic traits using genome-wide selection for the instruments.

Associations with P < 0.05 are denoted with a single asterisk, and associations with P<0.0011 (0.05/number of cytokines) are denoted with a double asterisk. CAD: coronary artery disease; T2DM: type 2 diabetes mellitus; BMI: body-mass index; WC: waist circumference; WHR: waist-hip-ratio; SBP: systolic blood pressure; HDL-C: high-density lipoprotein cholesterol; LDL-C: low-density lipoprotein cholesterol; TC: total cholesterol; TG: triglycerides; CRP: C-reactive protein; FI: fasting insulin. The abbreviations for the cytokines are given in Supplementary Table 1.

Supplementary Figures 10a-t. Colocalization plots for circulating cytokine levels and cardiometabolic traits considered as outcomes.

Cytokine-outcome pairs with both Mendelian randomization evidence (P<0.0033) and colocalization evidence ($PP_{shared} + PP_{distinct} > 0.5$ and $PP_{shared} /(PP_{shared} + PP_{distinct}) > 0.5$; panels a-s) for causality, or only Mendelian randomization evidence for coronary artery disease risk (panel t) are plotted within ±500kb of the coding gene of the exposure cytokine. pQTL=protein quantitative trait loci. R^2 = linkage disequilibrium correlation based on the 1000Genomes European reference panel. PP = posterior probability. Available at: https://doi.org/10.5281/zenodo.7215468.

Supplementary Table 1. Cytokines and their data sources for the genome-wide association study.

NFBC1966 = Northern Finland Birth Cohort 1966; YFS = Young Finns Study; FINRISK = FINRISK Study. *The total sample size with full genomic and cytokine data after quality control.

		Sample size			Total sample size*
Cytokine	Abbreviation	NFBC1966	FINRISK	YFS	
Active plasminogen activator inhibitor-1	activePAI1	5199			5199
Beta nerve growth factor	βNGF		1620	1950	3531
Cutaneous T-cell attracting chemokine (CCL27)	CTACK		1651	2019	3631
Eotaxin (CCL11)	Eotaxin		6186	2011	8153
Basic fibroblast growth factor	FGFBasic		5592	2017	7565
Granulocyte colony-stimulating factor	GCSF		1544	2018	7904
Growth regulated oncogene-alpha (CXCL1)	GROα		1541	2003	3505
Hepatocyte growth factor	HGF		6317	2019	8292
Interferon-gamma	IFNγ		5726	2019	7701
Interleukin-10	IL10		5708	2016	7681
Interleukin-12p70	IL12p70		6295	2019	8270
Interleukin-13	IL13		1577	2019	3557
Interleukin-16	IL16		1663	1858	3483
Interleukin-17	IL17	5071	5785	2019	12831
Interleukin-18	IL18		1656	2019	3636
Interleukin-1-alpha	IL1α	5014			5014
Interleukin-1-beta	IL1β	5067	1330	2018	8376
Interleukin-1 receptor antagonist	IL1ra	4957	1658	2019	8595
Interleukin-2	IL2		1498	2016	3475
Interleukin-2 receptor, alpha subunit	IL2rα		1704	2012	3677
Interleukin-4	IL4	5059	6149	2019	13183
Interleukin-5	IL5		1386	2017	3364
Interleukin-6	IL6	5063	6215	2018	13252
Interleukin-7	IL7		1429	2019	3409
Interleukin-8 (CXCL8)	IL8	5071	1546	2019	8597

Karhunen V, *et al. BMJMED* 2023; 2:e000157. doi: 10.1136/bmjmed-2022-000157

Interleukin-9	IL9		1656	2017	3634
Interferon gamma-induced protein 10 (CXCL10)	IP10	5072	1705	2019	8757
Monocyte chemotactic protein-1 (CCL2)	MCP1	5072	6318	2019	13365
Monocyte specific chemokine 3 (CCL7)	MCP3		843	256	843
Macrophage colony-stimulating factor	MCSF		1632	866	840
Macrophage migration inhibitory factor (glycosylation-inhibiting factor)	MIF		1516	2017	3494
Monokine induced by interferon-gamma (CXCL9)	MIG		1705	2019	3685
Macrophage inflammatory protein-1a (CCL3)	MIP1α		1542	2019	3522
Macrophage inflammatory protein-1b (CCL4)	MIP1β		6268	2019	8243
Platelet derived growth factor BB	PDGFbb		6318	2019	8293
Regulated on Activation, Normal T Cell Expressed and Secreted (CCL5)	RANTES		1585	1869	3421
Soluble CD40 ligand	sCD40L	5067			5067
Stem cell factor	SCF		6316	2018	8290
Stem cell growth factor beta	SCGFβ		1704	2017	3682
Stromal cell-derived factor-1 alpha (CXCL12)	SDF1α		6003	1826	5998
soluble E-selectin	sE-selectin	5199			5199
soluble intercellular adhesion molecule-1	sICAM1	5199			5199
soluble vascular cell adhesion molecule 1	sVCAM1	5199			5199
Tumor necrosis factor-alpha	TNFα	5068	1474	2019	8522
Tumor necrosis factor-beta	TNFβ		1450	116	1559
TNF-related apoptosis inducing ligand	TRAIL		6218	2012	8186
Vascular endothelial growth factor	VEGF	5037	5143	2019	12155

Supplementary Table 2. Sources for genetic association estimates with cardiometabolic traits.

Trait	Sample size	Population ancestry	Reference
Coronary artery disease	60,801 cases<br>123,504 controls	Multi-ancestry	Nikpay et al. 2015
Stroke	40,585 cases<br>406,111 controls	European	Malik et al. 2015
Type 2 diabetes	74,124 cases<br>824,006 controls	European	Mahajan et al. 2018
Body mass index	806,834	European	Pulit et al. 2019
Waist circumference	210,088	European	Shungin et al. 2015
Waist-hip-ratio	210,088	European	Shungin et al. 2015
Systolic blood pressure	318,419	European	Carter et al. 2019
High-density lipoprotein cholesterol	1,320,016	European	Graham et al. 2021
Low-density lipoprotein cholesterol	1,320,016	European	Graham et al. 2021
Total cholesterol	1,320,016	European	Graham et al. 2021
Triglycerides	1,320,016	European	Graham et al. 2021
C-reactive protein[1]	343,524	European	Neale lab 2020
Glucose	46,186	European	Dupuis et al. 2010
Fasting insulin	38,238	European	Dupuis et al. 2010
Glycated hemoglobin	123,665	European	Wheeler et al. 2017
Lifetime smoking[2]	462,690	European	Wootton et al. 2019

[1]CRP is a biomarker for systemic inflammation and for this reason was considered only as an outcome trait rather than an exposure. [2]Smoking is robustly associated with cardiometabolic outcomes, but it is largely a behavioural trait and therefore was considered only as an exposure trait.

Karhunen V, *et al. BMJMED* 2023; 2:e000157. doi: 10.1136/bmjmed-2022-000157

Supplementary Table 3. Cytokine coding genes, data sources and the number of instruments with two different criteria.

Chr: chromosome; GWAS: genome-wide association study; pQTL: protein quantitative trait loci; eQTL: expression quantitative trait loci. INTERVAL study: ref. [24]; SCALLOP study: ref. [25]. *Interleukin-12p70 has two coding genes.

Cytokine	Abbreviation	Gene	Chr	Start position (hg19)	End position (hg19)	Source	GWAS sample size	Number of *cis*-pQTL instruments	Number of *cis*-eQTL instruments
Active plasminogen activator inhibitor-1	activePAI1	SERPINE1	7	100770370	100782547	Current GWAS	5199	1	0
Beta nerve growth factor	βNGF	NGF	1	115828537	115880857	Current GWAS	3531	0	0
Cutaneous T-cell attracting chemokine (CCL27)	CTACK	CCL27	9	34661893	34662689	Current GWAS	3631	6	2
Eotaxin (CCL11)	Eotaxin	CCL11	17	32612687	32615199	Current GWAS+INTERVAL	11454	2	1
Basic fibroblast growth factor	FGFBasic	FGF2	4	123747863	123819390	Current GWAS	7565	0	1
Granulocyte colony-stimulating factor	GCSF	CSF3	17	38171614	38174066	Current GWAS	7904	0	0
Growth regulated oncogene-alpha (CXCL1)	GROα	CXCL1	4	74735109	74737019	Current GWAS+INTERVAL	6806	26	1
Hepatocyte growth factor	HGF	HGF	7	81331444	81399452	Current GWAS+SCALLOP	25205	1	0
Interferon-gamma	IFNγ	IFNG	12	68548550	68553521	Current GWAS	7701	0	0
Interleukin-10	IL10	IL10	1	206940948	206945839	Current GWAS	7681	0	0
Interleukin-12p70	IL12p70	IL12A*	3	159706623	159713806	Current GWAS	8270	1	0
		IL12B*	5	158741791	158757481			0	0
Interleukin-13	IL13	IL13	5	131993865	131996801	Current GWAS	3557	0	0
Interleukin-16	IL16	IL16	15	81517640	81605104	Current GWAS+SCALLOP	22678	1	0
Interleukin-17	IL17	IL17A	6	52051185	52055436	Current GWAS	12831	0	0
Interleukin-18	IL18	IL18	11	112013974	112034840	Current GWAS+SCALLOP	22831	7	2
Interleukin-1-alpha	IL1α	IL1A	2	113531492	113542971	Current GWAS	5014	0	1
Interleukin-1-beta	IL1β	IL1B	2	113587337	113594356	Current GWAS	8376	0	0
Interleukin-1 receptor antagonist	IL1ra	IL1RN	2	113885138	113891593	SCALLOP	19059	24	2
Interleukin-2	IL2	IL2	4	123372626	123377650	Current GWAS	3475	0	0

Interleukin-2 receptor, alpha subunit	IL2rα	IL2RA	10	6052657	6104333	Current GWAS	3677	12	4
Interleukin-4	IL4	IL4	5	132009678	132018370	Current GWAS	13183	0	0
Interleukin-5	IL5	IL5	5	131877136	131879214	Current GWAS	3364	0	0
Interleukin-6	IL6	IL6	7	22766766	22771621	SCALLOP	14244	0	0
Interleukin-7	IL7	IL7	8	79645007	79717758	Current GWAS	3409	1	0
Interleukin-8 (CXCL8)	IL8	IL8	4	74606223	74609433	SCALLOP	16924	1	2
Interleukin-9	IL9	IL9	5	135227935	135231516	Current GWAS	3634	0	0
Interferon gamma-induced protein 10 (CXCL10)	IP10	CXCL10	4	76942269	76944689	Current GWAS	8757	4	1
Monocyte chemotactic protein-1 (CCL2)	MCP1	CCL2	17	32582296	32584220	Current GWAS+SCALLOP	35100	5	2
Monocyte specific chemokine 3 (CCL7)	MCP3	CCL7	17	32597235	32599261	INTERVAL	3301	14	0
Macrophage colony-stimulating factor	MCSF	CSF1	1	110453233	110473616	SCALLOP	16935	12	2
Macrophage migration inhibitory factor (glycosylation-inhibiting factor)	MIF	MIF	22	24236565	24237409	Current GWAS	3494	2	1
Monokine induced by interferon-gamma (CXCL9)	MIG	CXCL9	4	76922623	76928641	Current GWAS	3685	2	2
Macrophage inflammatory protein-1a (CCL3)	MIP1α	CCL3	17	34415603	34417506	SCALLOP	19060	35	1
Macrophage inflammatory protein-1b (CCL4)	MIP1β	CCL4	17	34431220	34433014	Current GWAS+SCALLOP	25175	39	2
Platelet derived growth factor BB	PDGFbb	PDGFB	22	39619685	39640957	SCALLOP	19205	1	0
Regulated on Activation, Normal T Cell Expressed and Secreted (CCL5)	RANTES	CCL5	17	34198496	34207377	Current GWAS	3421	1	1
Soluble CD40 ligand	sCD40L	CD40LG	X	135730336	135742549	SCALLOP	19190	0	0
Stem cell factor	SCF	KITLG	12	88886570	88974250	SCALLOP	19206	2	0
Stem cell growth factor beta	SCGFβ	CLEC11A	19	51226605	51228981	Current GWAS+INTERVAL	6983	4	1

Stromal cell-derived factor-1 alpha (CXCL12)	SDF1α	CXCL12	10	44872510	44880545	Current GWAS	5998	0	0
soluble E-selectin	sE-selectin	SELE	1	169691781	169703220	Current GWAS+SCALLOP	26504	5	0
soluble intercellular adhesion molecule-1	sICAM1	ICAM1	19	10381517	10397291	Current GWAS	5199	26	1
soluble vascular cell adhesion molecule-1	sVCAM1	VCAM1	1	101185196	101204601	Current GWAS	5199	1	1
Tumor necrosis factor-alpha	TNFα	TNF	6	31543344	31546112	Current GWAS	8522	2	0
Tumor necrosis factor-beta	TNFβ	LTA	6	31539876	31542100	INTERVAL	1559	0	0
TNF-related apoptosis inducing ligand	TRAIL	TNFSF10	3	172223298	172241297	Current GWAS+SCALLOP	24135	14	4
Vascular endothelial growth factor	VEGF	VEGFA	6	43737946	43754223	Current GWAS+SCALLOP	29080	34	1

Supplementary Table 4. Variants used as instrumental variables for cardiometabolic traits and cytokines using genome-wide selection.

Chr = chromosome; Pos = position; EA = effect allele; NEA = non-effect allele; EAF = effect allele frequency; BETA = effect size estimate; SE = standard error; P = p-value; R2 = variance explained; F = F-statistic. Available at: https://doi.org/10.5281/zenodo.7215468.

Supplementary Table 5. Table comparing Mendelian randomization (MR) estimates for the effects of genetically predicted cardiometabolic traits on circulating cytokine levels using different MR methods.

N_SNPs = number of instrumental variables; CIL = 95% confidence interval, lower limit; CIU = 95% confidence interval, upper limit; IVW = inverse-variance weighted method; WM = weighted median method. Available at: https://doi.org/10.5281/zenodo.7215468.

Supplementary Table 6. Variants used as instrumental variables for cytokines selected by *cis*-protein quantitative trait loci instrument selection.

Chr = chromosome; Pos = position; EA = effect allele; NEA = non-effect allele; EAF = effect allele frequency; SE = standard error; P = p-value; F = F-statistic. Available at: https://doi.org/10.5281/zenodo.7215468.

Karhunen V, *et al. BMJMED* 2023; 2:e000157. doi: 10.1136/bmjmed-2022-000157

Supplementary Table 7. Variants used as instrumental variables for cytokines selected by *cis*-expression quantitative trait loci instrument selection.

Chr = chromosome; Pos = position; EA = effect allele; NEA = non-effect allele; EAF = effect allele frequency; SE = standard error; P = p-value; F = F-statistic. Available at: https://doi.org/10.5281/zenodo.7215468.

Supplementary Table 8. Table comparing Mendelian randomization (MR) estimates for the effects of genetically predicted cytokine levels on other circulating cytokine levels using different MR methods.

N_SNPs = number of instrumental variables; CIL = 95% confidence interval, lower limit; CIU = 95% confidence interval, upper limit; IVW = inverse-variance weighted method; WM = weighted median method; eQTL = expression quantitative trait loci; pQTL = protein quantitative trait loci. Available at: https://doi.org/10.5281/zenodo.7215468.

Supplementary Table 9. Colocalization results for the genetic associations of circulating cytokine levels within ±500kb of the coding gene of the exposure cytokine.

Only cytokine-cytokine pairs with MR-evidence for causality (P<0.0011) are reported. PP = posterior probability. Available at: https://doi.org/10.5281/zenodo.7215468.

Supplementary Table 10. Table comparing Mendelian randomization (MR) estimates for the effects of genetically predicted cytokine levels on cardiometabolic phenotypes considered as outcomes using different MR methods.

N_SNPs = number of instrumental variables; CIL = 95% confidence interval, lower limit; CIU = 95% confidence interval, upper limit; IVW = inverse-variance weighted method; WM = weighted median method; eQTL = expression quantitative trait loci; pQTL = protein quantitative trait loci. Available at: https://doi.org/10.5281/zenodo.7215468.

Supplementary Table 11. Table comparing Mendelian randomization (MR) estimates for the effects of genetically predicted circulating cytokine levels on cardiometabolic traits using genome-wide instrument selection.

N_SNPs = number of instrumental variables; CIL = 95% confidence interval, lower limit; CIU = 95% confidence interval, upper limit; IVW = inverse-variance weighted method; WM = weighted median method. Available at: https://doi.org/10.5281/zenodo.7215468.

Supplementary Table 12. Colocalization results for the genetic associations of circulating cytokine levels and cardiometabolic phenotypes considered as outcomes within ±500kb of the coding gene of the cytokine.

Cytokine-outcome pairs with nominal MR-evidence for causality (*P*<0.05) are reported. PP = posterior probability. Available at: https://doi.org/10.5281/zenodo.7215468.

Karhunen V, *et al. BMJMED* 2023; 2:e000157. doi: 10.1136/bmjmed-2022-000157
