# Supplementary material for: The interplay between inflammatory cytokines and cardiometabolic disease: bi-directional mendelian randomisation study
Source: BMJ Med. 2023 Feb 14;2(1):e000157. doi: 10.1136/bmjmed-2022-000157 (PMC9978757; doi:10.1136/bmjmed-2022-000157)
Supplement: Supplementary data [file bmjmed-2022-000157supp003.pdf]

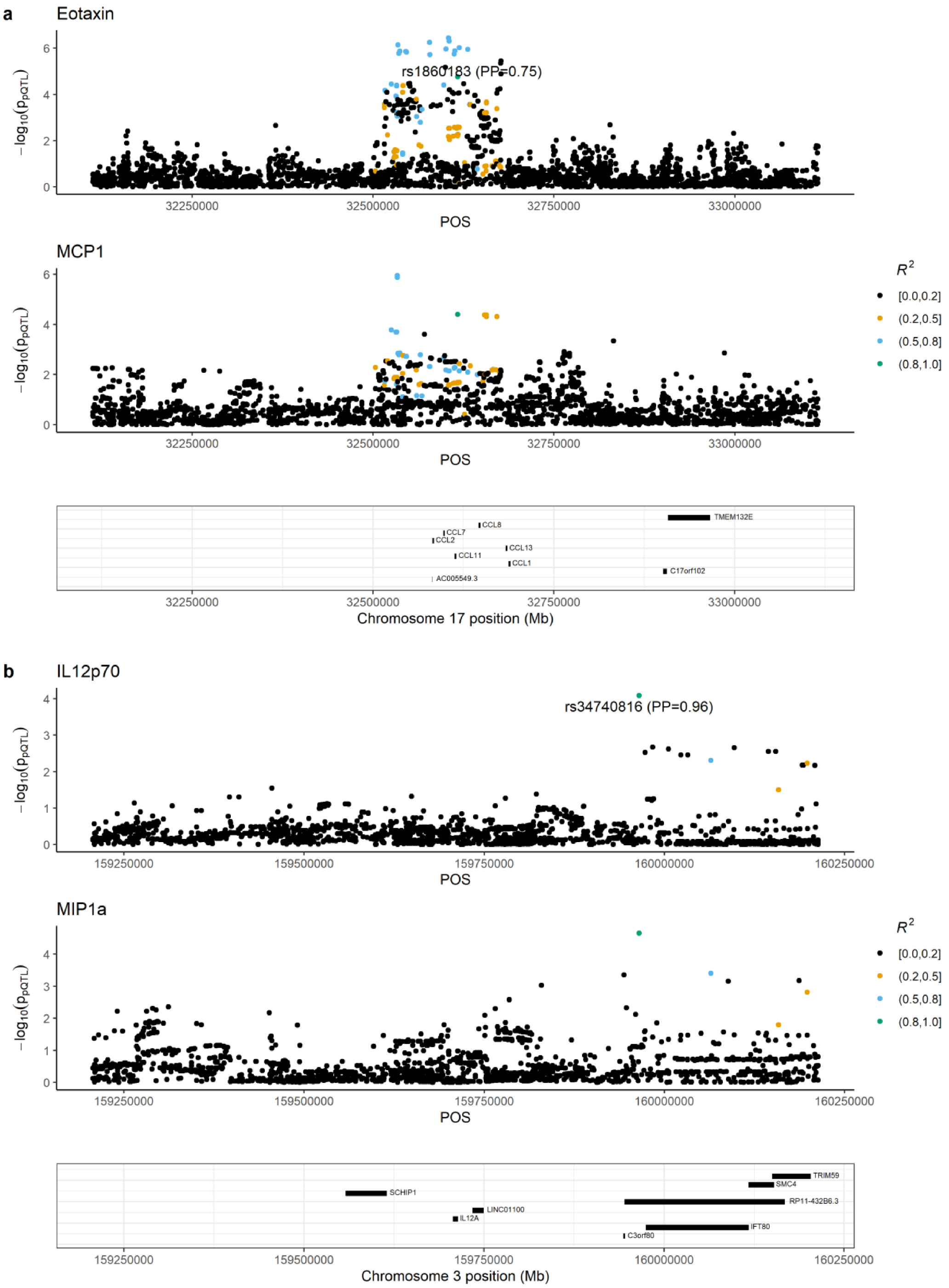
a
Eotaxin
rs1860183 (PP=0.75)
$-\log_{10}(p_{pQTL})$
POS
MCP1
R^2
[0.0,0.2]
(0.2,0.5]
(0.5,0.8]
(0.8,1.0]
TMEM132E
CCL8
CCL7
CCL2
CCL13
CCL11
CCL1
C17orf102
AC005549.3
32250000
32500000
32750000
33000000
Chromosome 17 position (Mb)
b
IL12p70
rs34740816 (PP=0.96)
MIP1a
TRIM59
SMC4
SCHIP1
RP11-432B6.3
LINC01100
IL12A
IFT80
C3orf80
159250000
159500000
159750000
160000000
160250000
Chromosome 3 position (Mb)

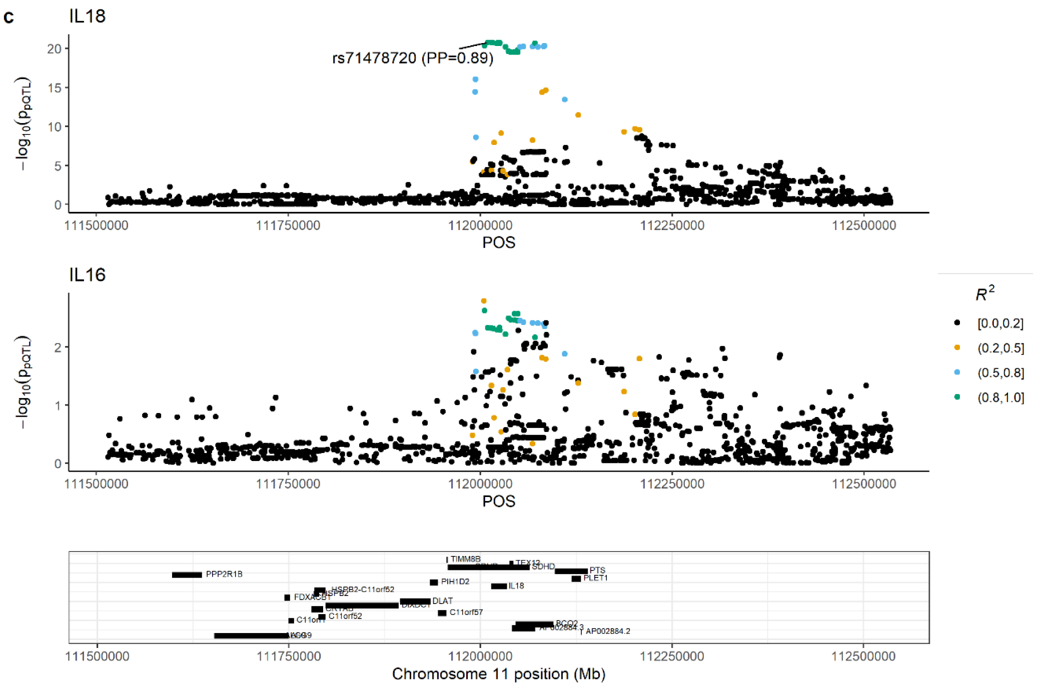
c
IL18
rs71478720 (PP=0.89)
$-\log_{10}(p_{pQTL})$
POS
IL16
R^2
[0.0,0.2]
(0.2,0.5]
(0.5,0.8]
(0.8,1.0]
PPP2R1B
TIMM8B
SDHD
PTS
PLET1
PIH1D2
IL18
DLAT
C11orf57
C11orf52
BCO2
AP002884.2
Chromosome 11 position (Mb)

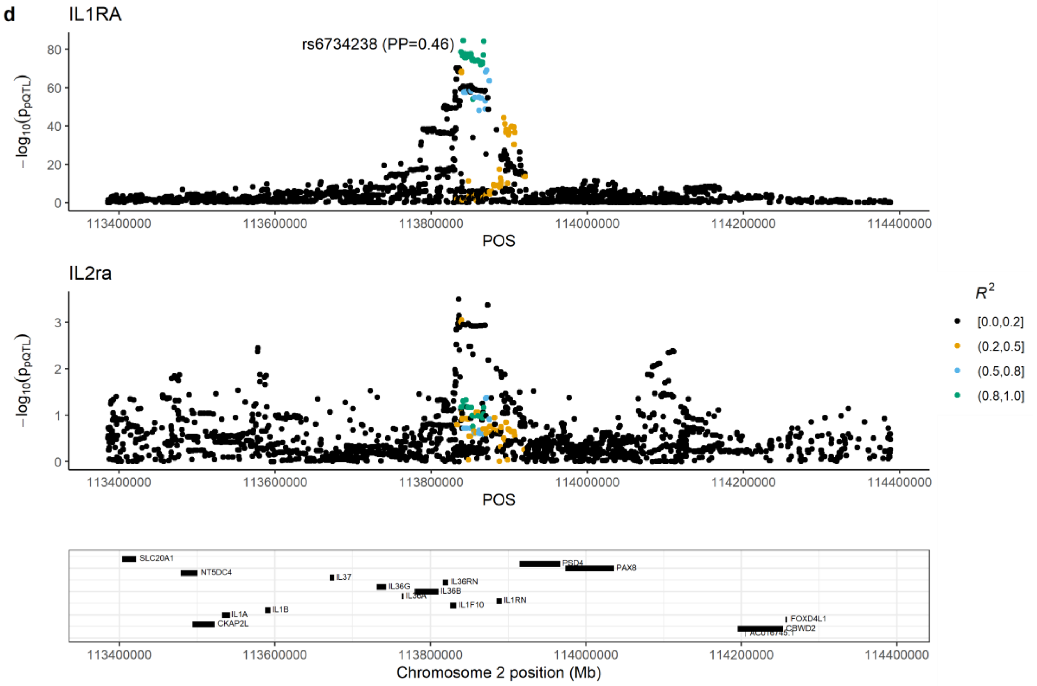
d
IL1RA
rs6734238 (PP=0.46)
$-\log_{10}(p_{pQTL})$
POS
IL2ra
R^2
[0.0,0.2]
(0.2,0.5]
(0.5,0.8]
(0.8,1.0]
SLC20A1
NT5DC4
IL37
IL36G
IL36RN
IL36B
IL1F10
IL1RN
PSD4
PAX8
IL1A
IL1B
CKAP2L
FOXD4L1
CBWD2
Chromosome 2 position (Mb)

e

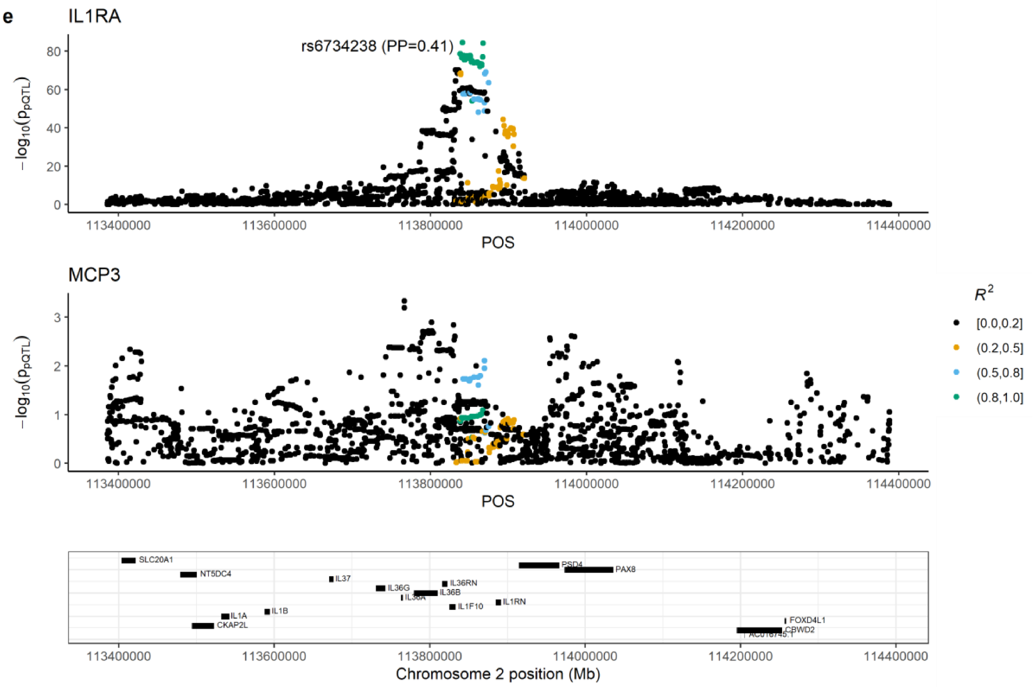
IL1RA
rs6734238 (PP=0.41)
−log10(pPQTL)
POS
MCP3
R^2
[0.0,0.2]
(0.2,0.5]
(0.5,0.8]
(0.8,1.0]
SLC20A1
NT5DC4
IL37
IL36G
IL36RN
IL36B
IL1F10
IL1RN
PSD4
PAX8
IL1A
IL1B
CKAP2L
FOXD4L1
CBWD2
113400000
113600000
113800000
114000000
114200000
114400000
Chromosome 2 position (Mb)

f

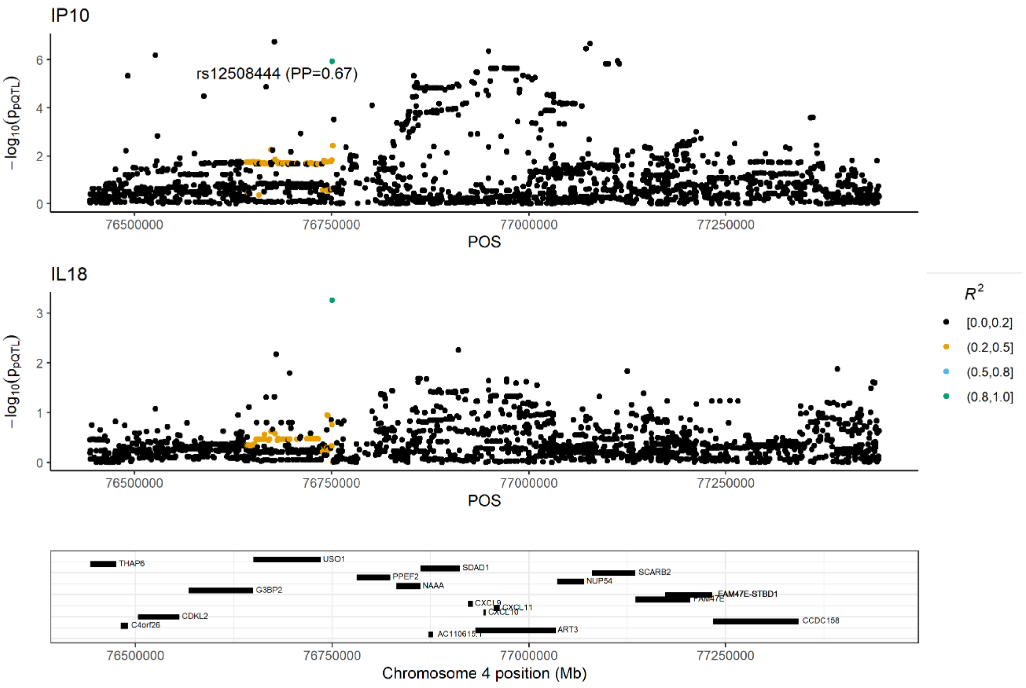
IP10
rs12508444 (PP=0.67)
−log10(pPQTL)
POS
IL18
R^2
[0.0,0.2]
(0.2,0.5]
(0.5,0.8]
(0.8,1.0]
THAP6
USO1
SDAD1
PPEF2
NAAA
G3BP2
SCARB2
NUP54
FAM47E-STBD1
FAM47E
CXCL9
CXCL11
CXCL10
CDKL2
C4orf26
ART3
CCDC158
76500000
76750000
77000000
77250000
Chromosome 4 position (Mb)

g

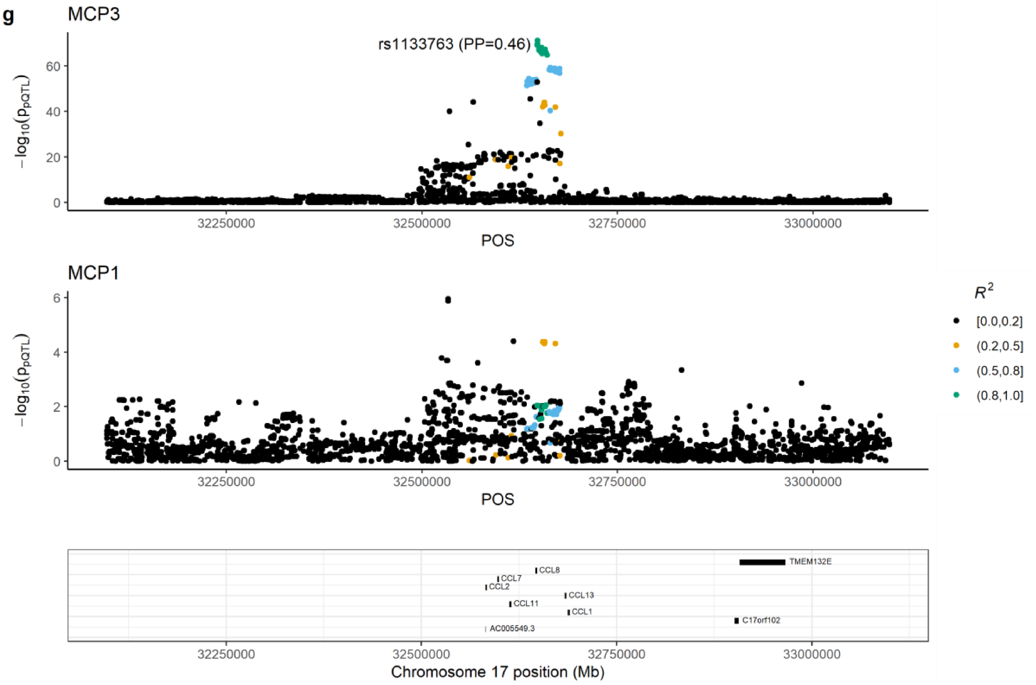
MCP3
rs1133763 (PP=0.46)
−log10(pPQTL)
POS
MCP1
R2
[0.0,0.2]
(0.2,0.5]
(0.5,0.8]
(0.8,1.0]
TMEM132E
CCL8
CCL7
CCL2
CCL11
CCL13
CCL1
C17orf102
AC005549.3
32250000
32500000
32750000
33000000
Chromosome 17 position (Mb)

h

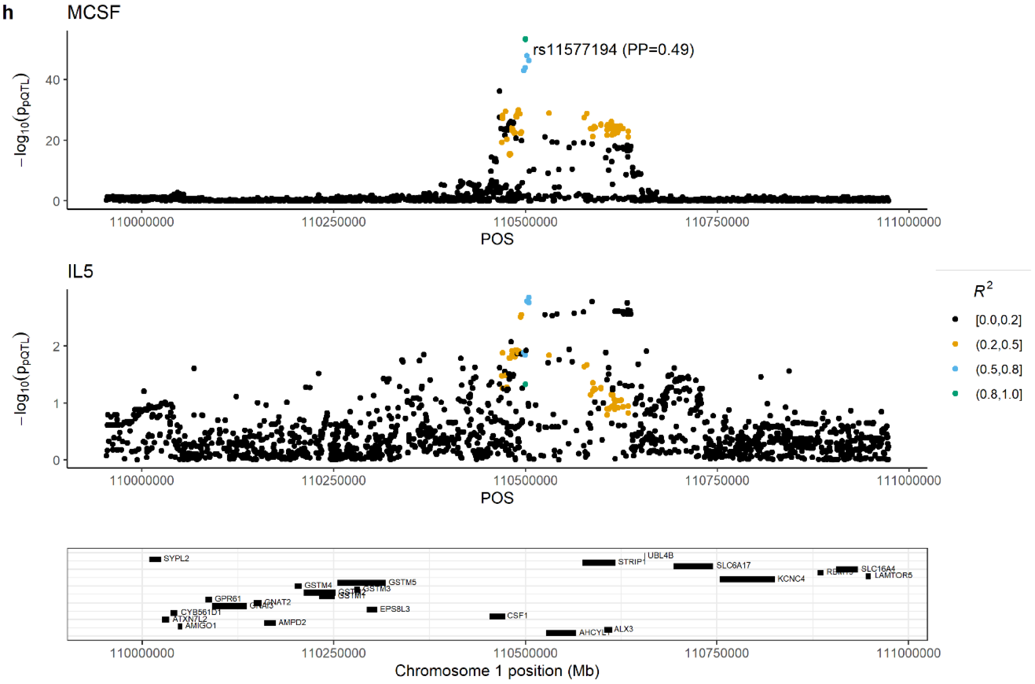
MCSF
rs11577194 (PP=0.49)
−log10(pPQTL)
POS
IL5
R2
[0.0,0.2]
(0.2,0.5]
(0.5,0.8]
(0.8,1.0]
SYPL2
UBL4B
STRIP1
SLC6A17
SLC16A4
LAMTOR5
KCNC4
GSTM4
GSTM5
GSTM3
GSTM1
GPR61
GNAT2
EPS8L3
CSF1
CYB561D1
ATXN7L2
AMIGO1
AMPD2
ALX3
110000000
110250000
110500000
110750000
111000000
Chromosome 1 position (Mb)

i

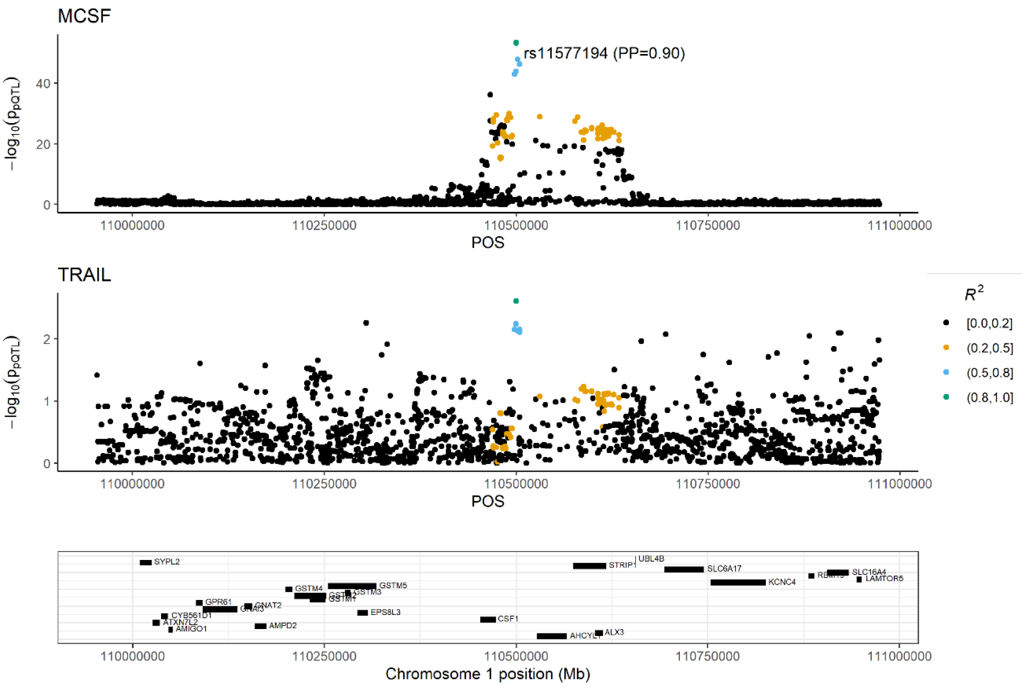
MCSF
rs11577194 (PP=0.90)
$-\log_{10}(p_{pQTL})$
POS
TRAIL
R^2
[0.0,0.2]
(0.2,0.5]
(0.5,0.8]
(0.8,1.0]
110000000
110250000
110500000
110750000
111000000
SYPL2
GSTM4
GSTM5
GPR61
GNAT2
AMPD2
EPS8L3
CSF1
AHCYL1
ALX3
STRIP1
UBL4B
SLC6A17
KCNC4
SLC16A4
LAMTOR5
Chromosome 1 position (Mb)

j

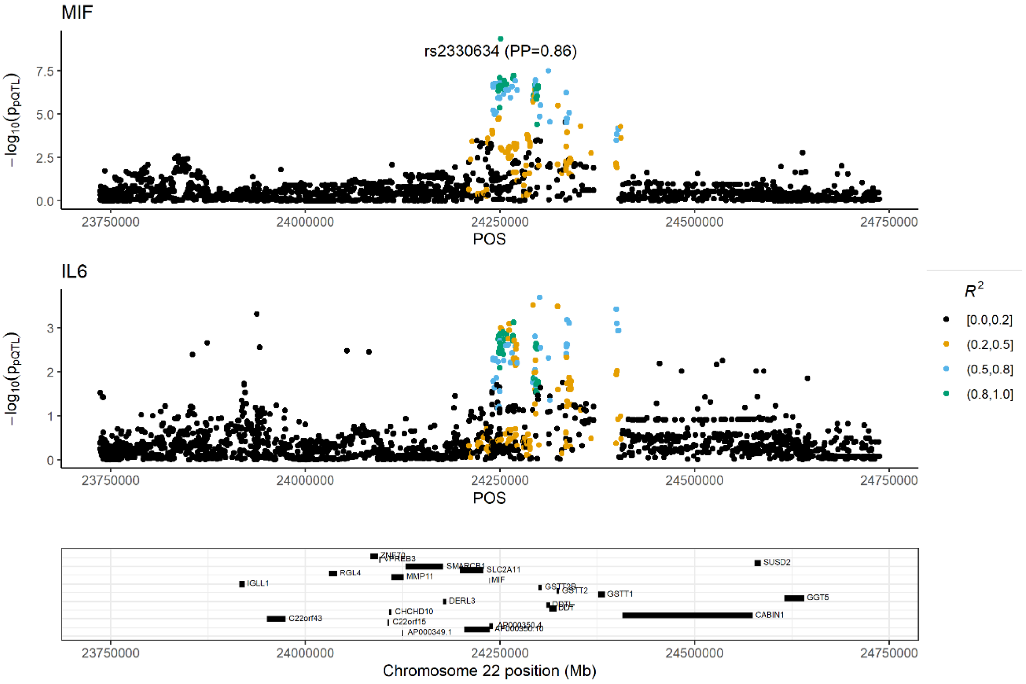
MIF
rs2330634 (PP=0.86)
$-\log_{10}(p_{pQTL})$
POS
IL6
R^2
[0.0,0.2]
(0.2,0.5]
(0.5,0.8]
(0.8,1.0]
23750000
24000000
24250000
24500000
24750000
RGL4
IGLL1
MMP11
SLC2A11
MIF
DERL3
CHCHD10
C22orf43
C22orf15
GSTT1
SUSD2
GGT5
CABIN1
Chromosome 22 position (Mb)

k

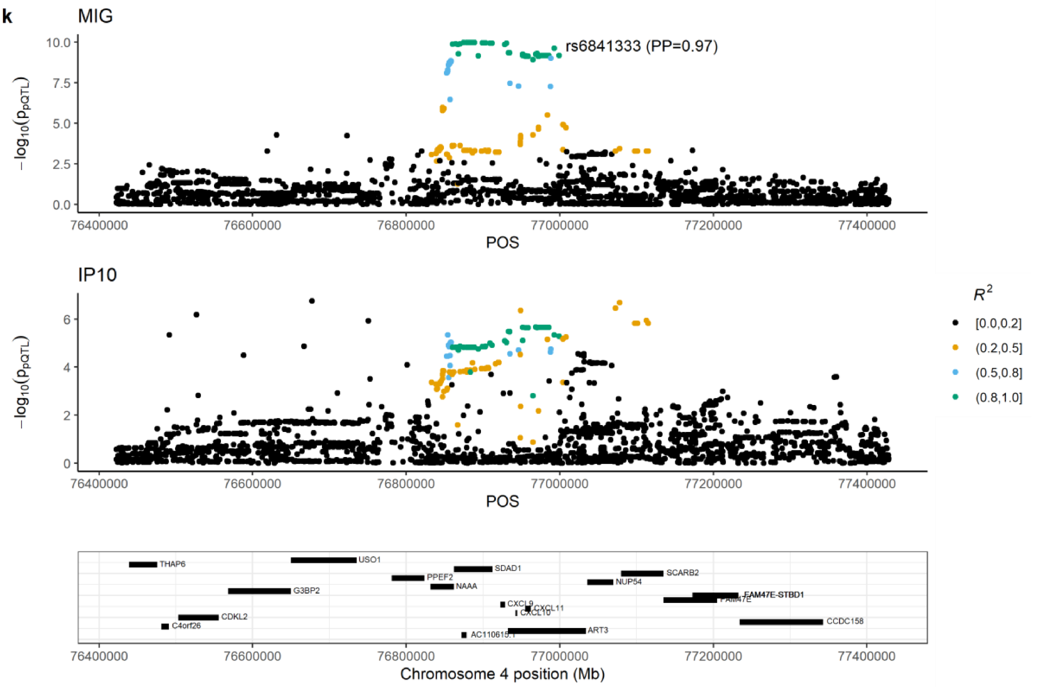
MIG
rs6841333 (PP=0.97)
$-\log_{10}(p_{pQTL})$
POS
IP10
R^2
[0.0,0.2]
(0.2,0.5]
(0.5,0.8]
(0.8,1.0]
Chromosome 4 position (Mb)

l

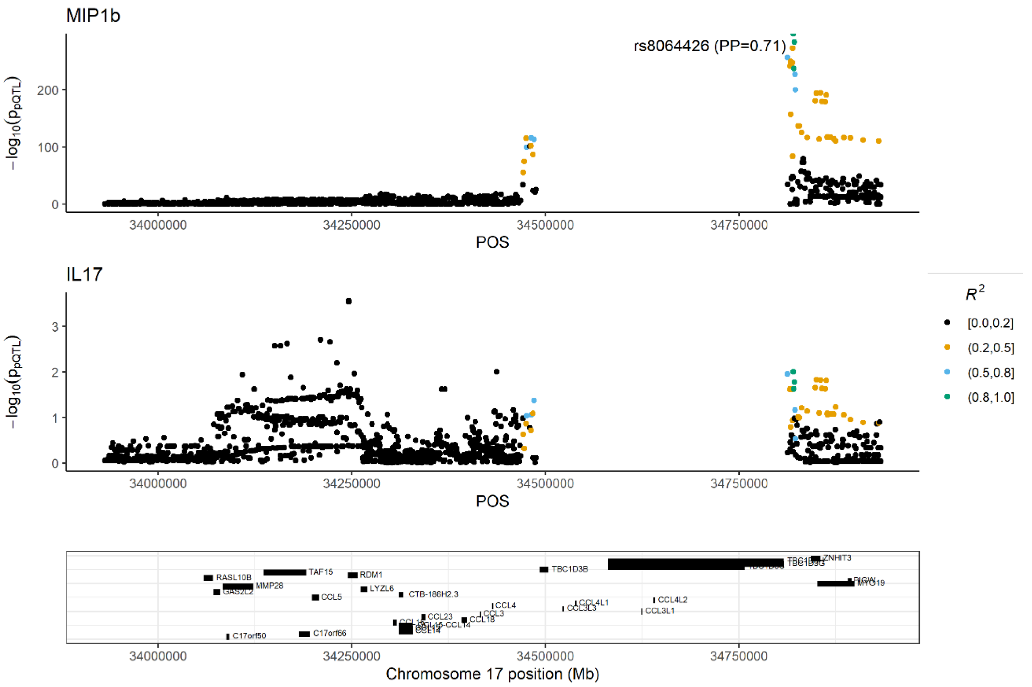
MIP1b
rs8064426 (PP=0.71)
$-\log_{10}(p_{pQTL})$
POS
IL17
R^2
[0.0,0.2]
(0.2,0.5]
(0.5,0.8]
(0.8,1.0]
Chromosome 17 position (Mb)

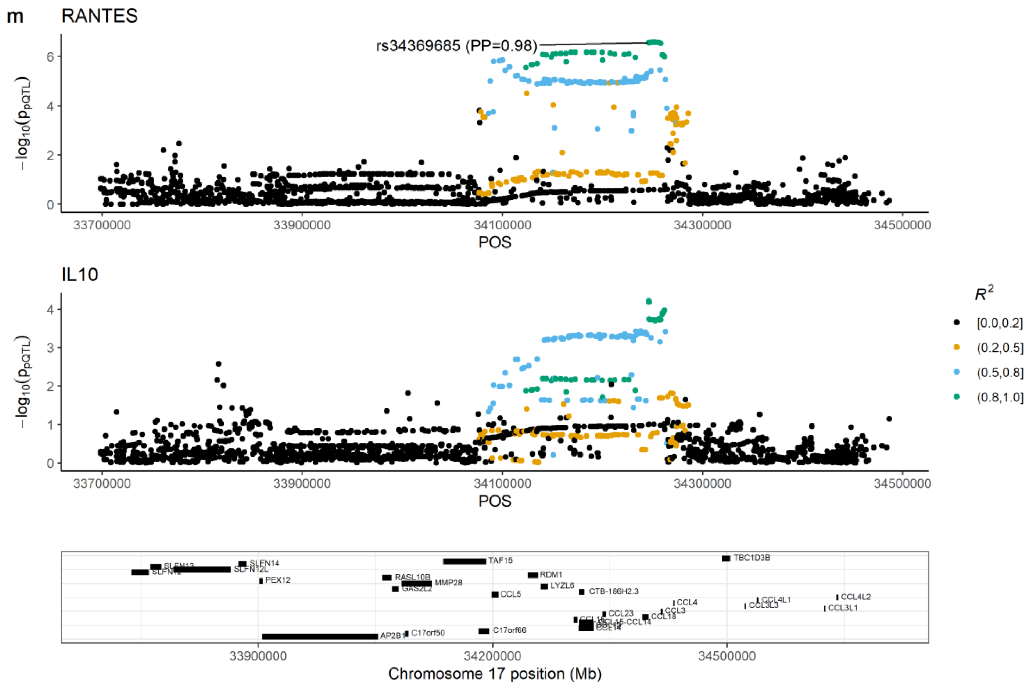
m
RANTES
rs34369685 (PP=0.98)
POS
IL10
POS
R^2
[0.0,0.2]
(0.2,0.5]
(0.5,0.8]
(0.8,1.0]
Chromosome 17 position (Mb)

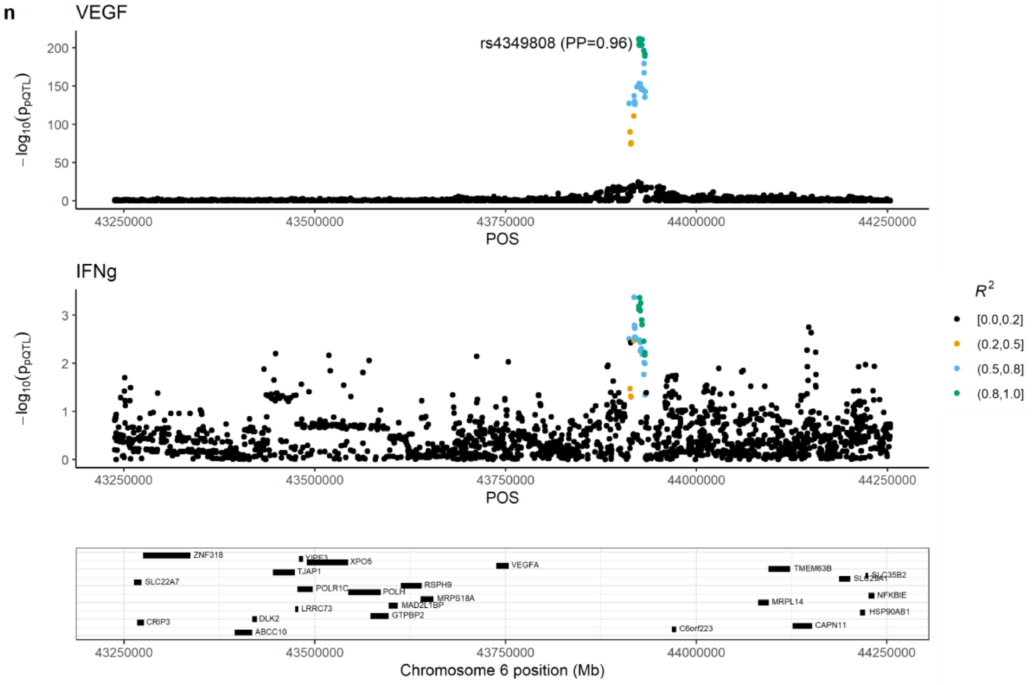
n
VEGF
rs4349808 (PP=0.96)
POS
IFNg
POS
R^2
[0.0,0.2]
(0.2,0.5]
(0.5,0.8]
(0.8,1.0]
Chromosome 6 position (Mb)

o

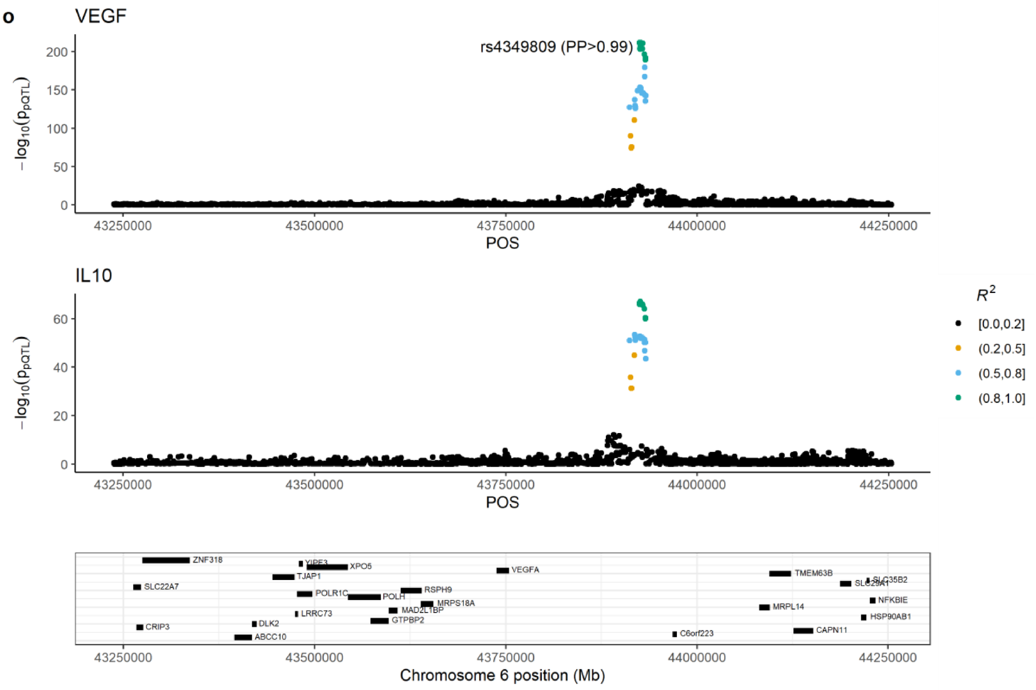
VEGF
rs4349809 (PP>0.99)
$-\log_{10}(p_{pQTL})$
POS
IL10
R^2
[0.0,0.2]
(0.2,0.5]
(0.5,0.8]
(0.8,1.0]
ZNF318
XPO5
TJAP1
VEGFA
TMEM63B
SLC22A7
POLR1C
POLH
RSPH9
MRPS18A
MAD2L1BP
LRRC73
GTPBP2
DLK2
CRIP3
ABCC10
C6orf223
MRPL14
NFKBIE
HSP90AB1
CAPN11
Chromosome 6 position (Mb)

p

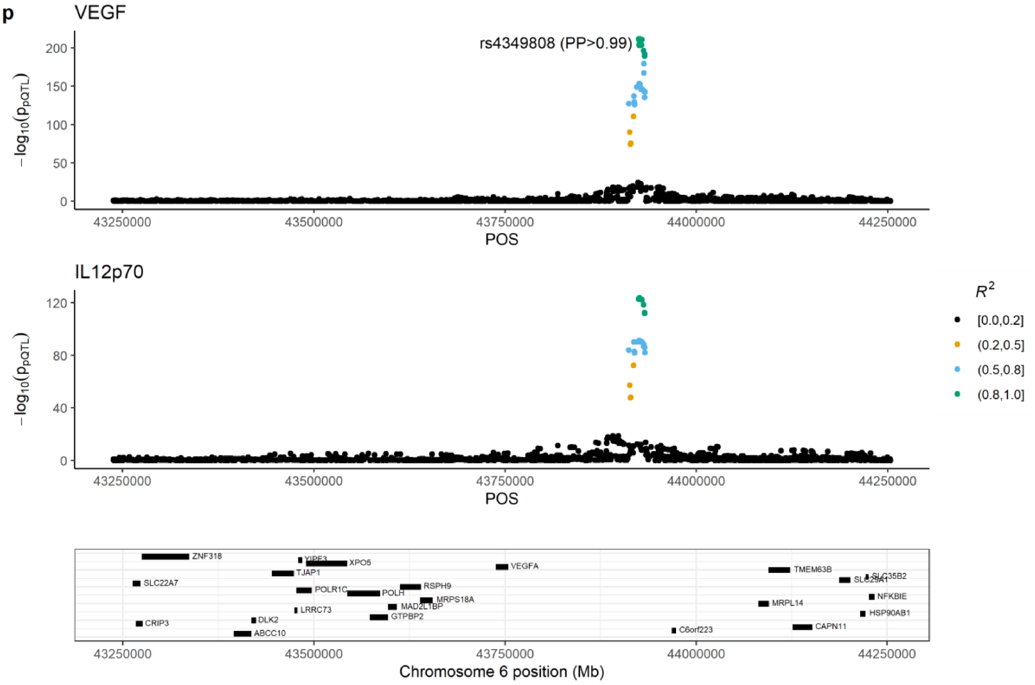
VEGF
rs4349808 (PP>0.99)
$-\log_{10}(p_{pQTL})$
POS
IL12p70
R^2
[0.0,0.2]
(0.2,0.5]
(0.5,0.8]
(0.8,1.0]
ZNF318
XPO5
TJAP1
VEGFA
TMEM63B
SLC22A7
POLR1C
POLH
RSPH9
MRPS18A
MAD2L1BP
LRRC73
GTPBP2
DLK2
CRIP3
ABCC10
C6orf223
MRPL14
NFKBIE
HSP90AB1
CAPN11
Chromosome 6 position (Mb)

q

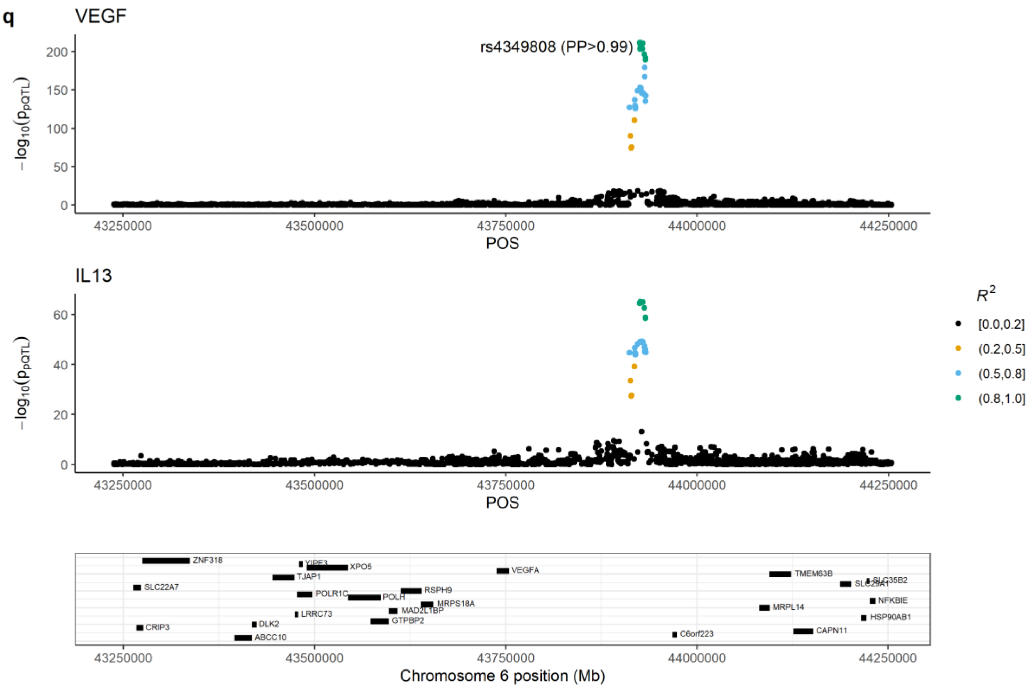
VEGF
rs4349808 (PP>0.99)
−log10(ppQTL)
POS
IL13
R2
[0.0,0.2]
(0.2,0.5]
(0.5,0.8]
(0.8,1.0]
Chromosome 6 position (Mb)
43250000
43500000
43750000
44000000
44250000
ZNF318
XPO5
TJAP1
VEGFA
TMEM63B
SLC22A7
POLR1C
POLH
RSPH9
MRPS18A
MAD2L1BP
GTPBP2
LRRC73
DLK2
ABCC10
CRIP3
C6orf223
MRPL14
CAPN11
NFKBIE
HSP90AB1

r

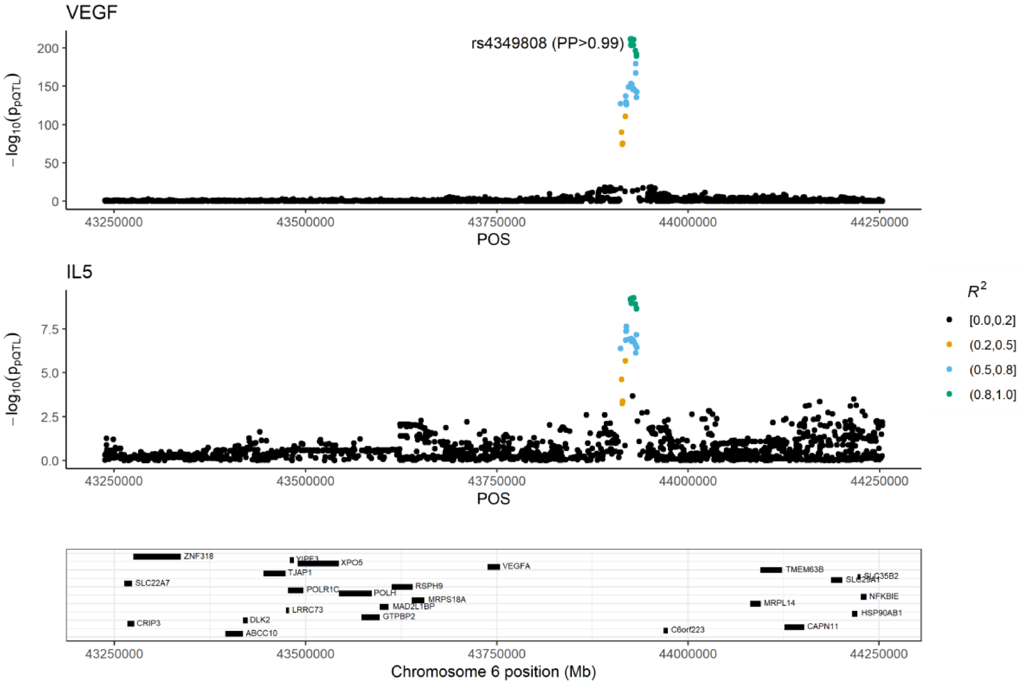
VEGF
rs4349808 (PP>0.99)
−log10(ppQTL)
POS
IL5
R2
[0.0,0.2]
(0.2,0.5]
(0.5,0.8]
(0.8,1.0]
Chromosome 6 position (Mb)
43250000
43500000
43750000
44000000
44250000
ZNF318
XPO5
TJAP1
VEGFA
TMEM63B
SLC22A7
POLR1C
POLH
RSPH9
MRPS18A
MAD2L1BP
GTPBP2
LRRC73
DLK2
ABCC10
CRIP3
C6orf223
MRPL14
CAPN11
NFKBIE
HSP90AB1

s

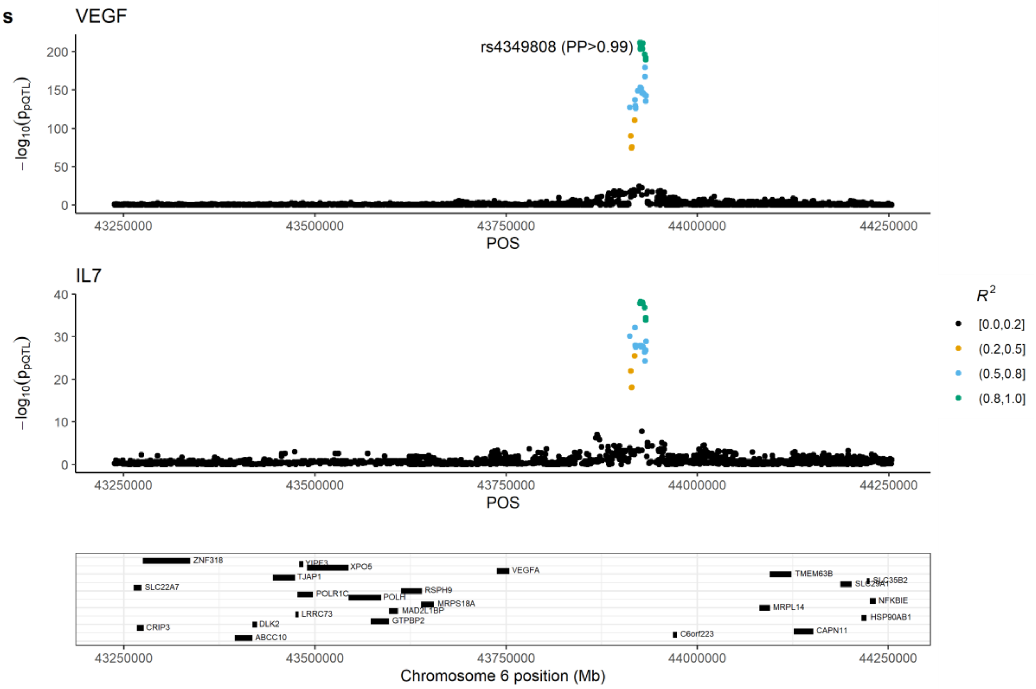
VEGF
rs4349808 (PP>0.99)
$-\log_{10}(p_{pQTL})$
POS
IL7
$-\log_{10}(p_{pQTL})$
POS
R^2
[0.0,0.2]
(0.2,0.5]
(0.5,0.8]
(0.8,1.0]
ZNF318
XPO5
VEGFA
TMEM63B
SLC29A1
SLC35B2
TJAP1
SLC22A7
POLR1C
POLH
RSPH9
MRPS18A
MAD2L1BP
NFKBIE
MRPL14
LRRC73
GTPBP2
HSP90AB1
DLK2
CRIP3
CAPN11
ABCC10
C6orf223
Chromosome 6 position (Mb)

t

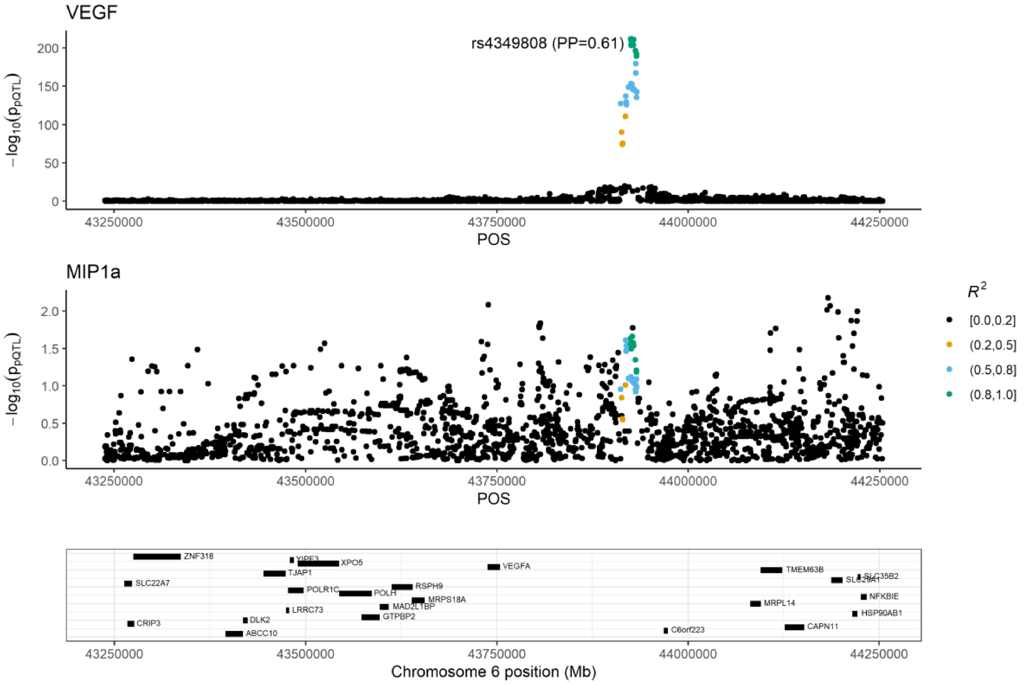
VEGF
rs4349808 (PP=0.61)
$-\log_{10}(p_{pQTL})$
POS
MIP1a
$-\log_{10}(p_{pQTL})$
POS
R^2
[0.0,0.2]
(0.2,0.5]
(0.5,0.8]
(0.8,1.0]
ZNF318
XPO5
VEGFA
TMEM63B
SLC29A1
SLC35B2
TJAP1
SLC22A7
POLR1C
POLH
RSPH9
MRPS18A
MAD2L1BP
NFKBIE
MRPL14
LRRC73
GTPBP2
HSP90AB1
DLK2
CRIP3
CAPN11
ABCC10
C6orf223
Chromosome 6 position (Mb)

Supplementary Figures 5a-t. Colocalization plots for circulating cytokine levels.

Cytokine-cytokine pairs with evidence for colocalization (PP_{shared} + $PP_{distinct}$ > 0.5 and PP_{shared} /(PP_{shared} + $PP_{distinct}$) > 0.5) within ±500kb of the coding gene of the exposure cytokine. pQTL=protein quantitative trait loci. R^2 = linkage disequilibrium correlation based on the 1000Genomes European reference panel. PP = posterior probability.
