# Supplementary material for: The interplay between inflammatory cytokines and cardiometabolic disease: bi-directional mendelian randomisation study
Source: BMJ Med. 2023 Feb 14;2(1):e000157. doi: 10.1136/bmjmed-2022-000157 (PMC9978757; doi:10.1136/bmjmed-2022-000157)
Supplement: Supplementary data [file bmjmed-2022-000157supp004.pdf]

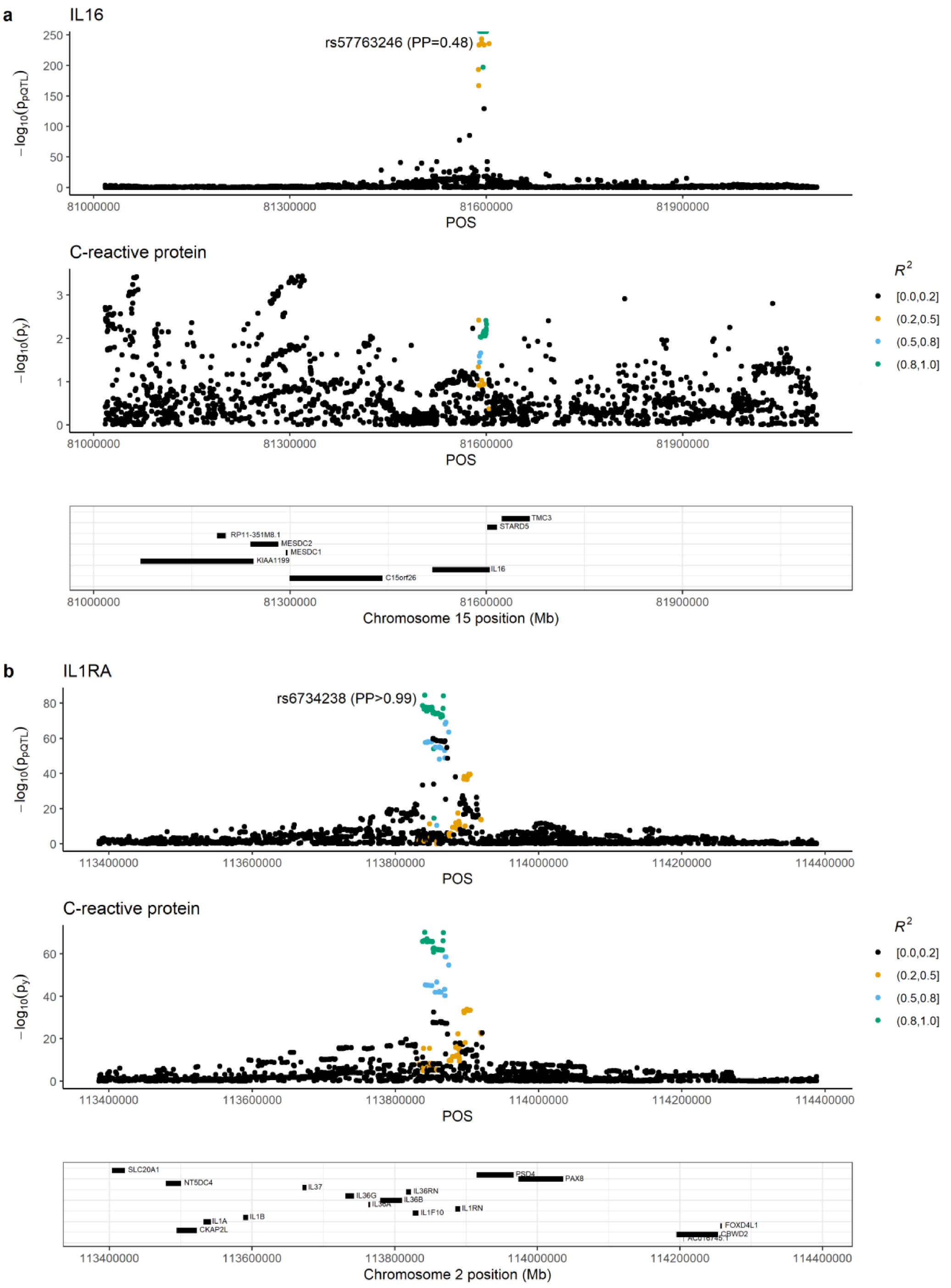
a
IL16
rs57763246 (PP=0.48)
−log10(pPQTL)
POS
C-reactive protein
−log10(py)
R2
[0.0,0.2]
(0.2,0.5]
(0.5,0.8]
(0.8,1.0]
Chromosome 15 position (Mb)
b
IL1RA
rs6734238 (PP>0.99)
C-reactive protein
Chromosome 2 position (Mb)

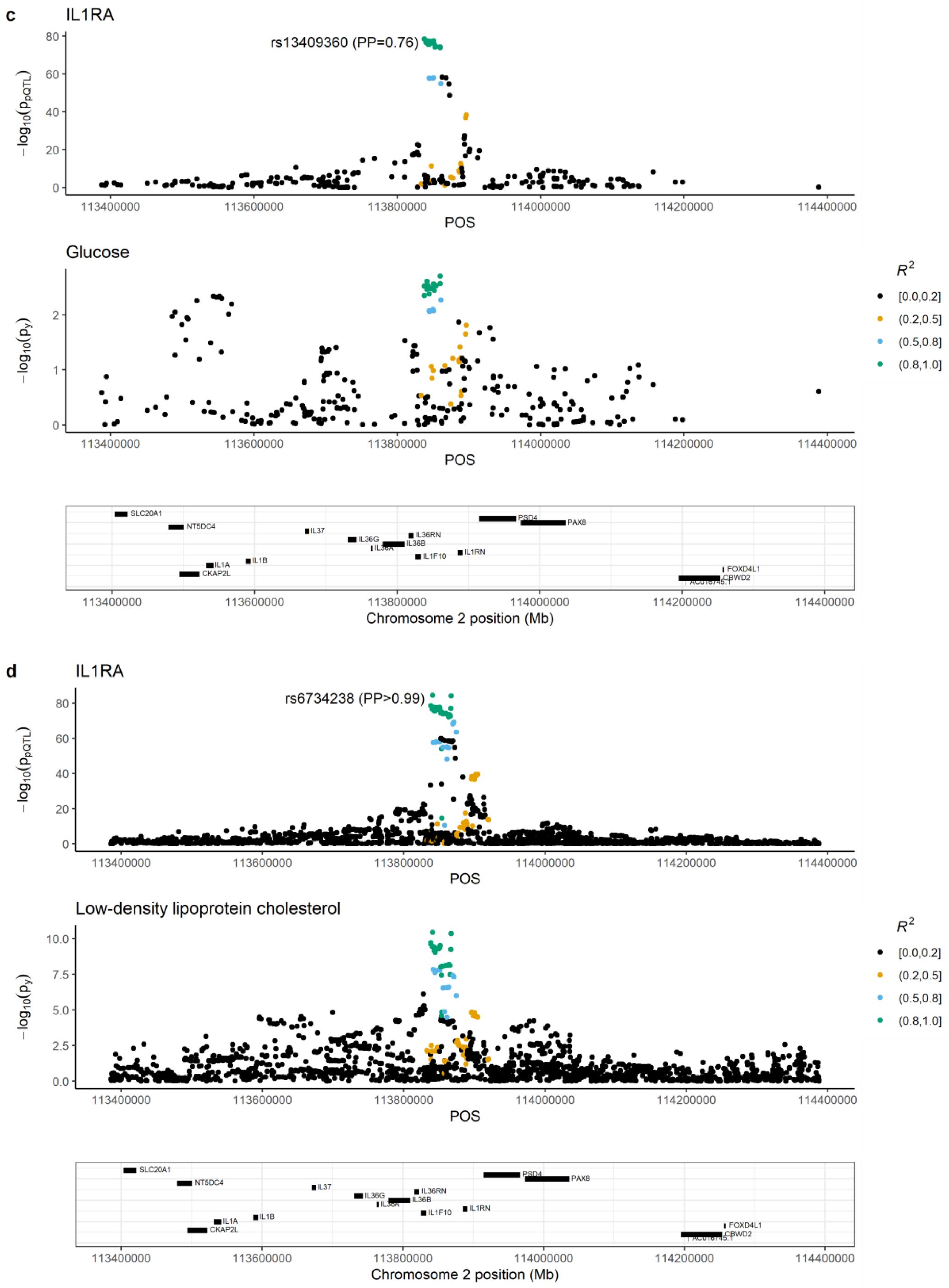
c
IL1RA
rs13409360 (PP=0.76)
$-\log_{10}(p_{pQTL})$
POS
Glucose
$-\log_{10}(p_y)$
R^2
[0.0,0.2]
(0.2,0.5]
(0.5,0.8]
(0.8,1.0]
SLC20A1
NT5DC4
IL37
IL36G
IL36A
IL36RN
IL36B
IL1F10
IL1RN
PSD4
PAX8
IL1A
IL1B
CKAP2L
FOXD4L1
CBWD2
Chromosome 2 position (Mb)
d
IL1RA
rs6734238 (PP>0.99)
Low-density lipoprotein cholesterol
113400000
113600000
113800000
114000000
114200000
114400000

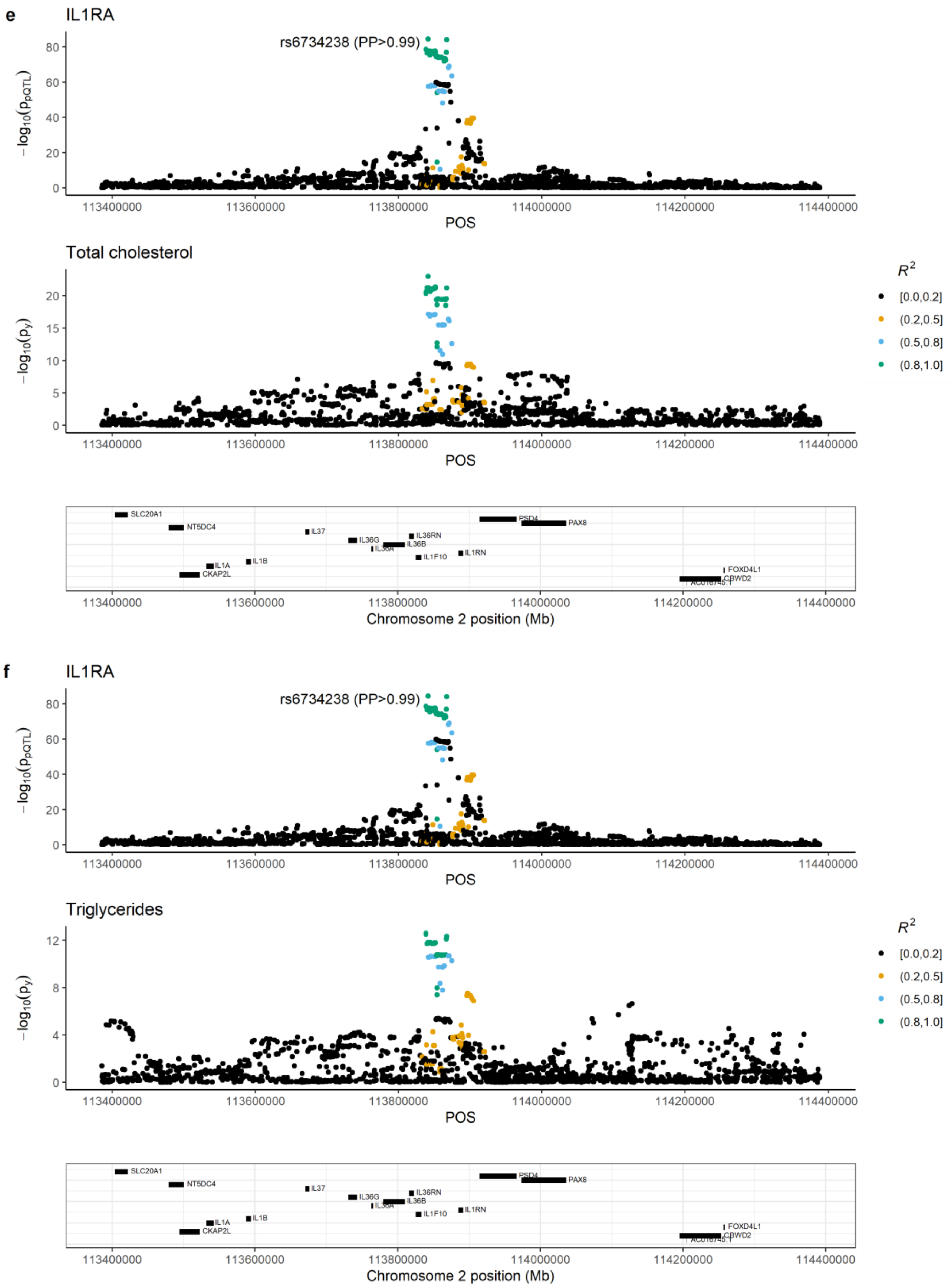
e
IL1RA
rs6734238 (PP>0.99)
−log10(pPQTL)
POS
Total cholesterol
−log10(py)
R2
[0.0,0.2]
(0.2,0.5]
(0.5,0.8]
(0.8,1.0]
SLC20A1
NT5DC4
IL37
IL36G
IL36A
IL36B
IL36RN
IL1F10
IL1RN
PSD4
PAX8
IL1A
IL1B
CKAP2L
FOXD4L1
CBWD2
Chromosome 2 position (Mb)
f
IL1RA
rs6734238 (PP>0.99)
Triglycerides
Chromosome 2 position (Mb)

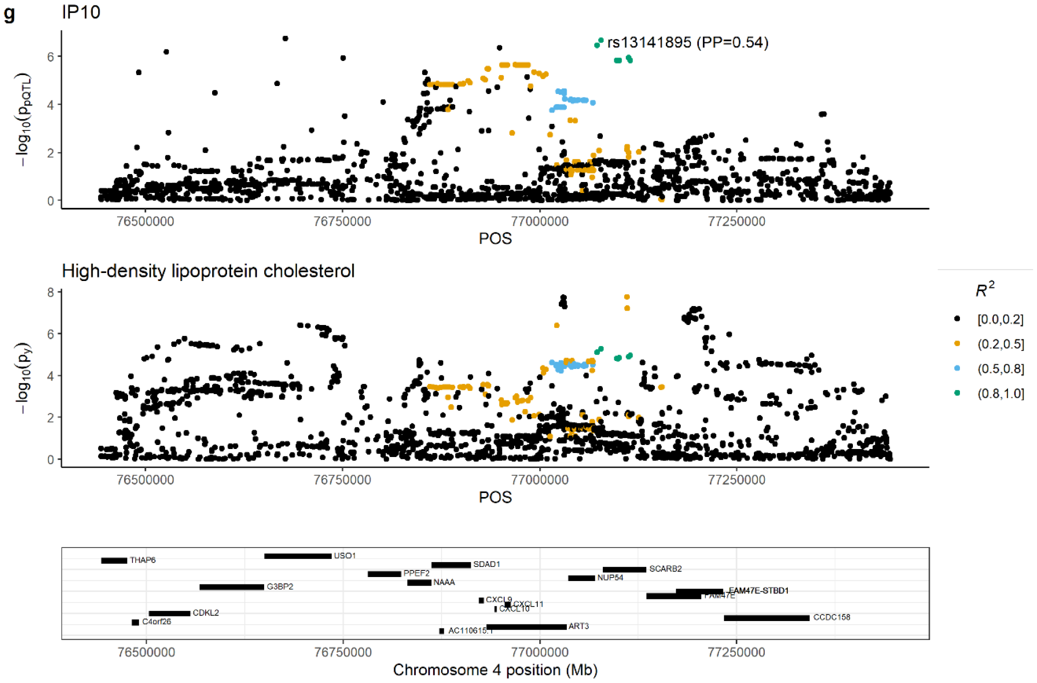
g
IP10
rs13141895 (PP=0.54)
$-\log_{10}(p_{pQTL})$
POS
High-density lipoprotein cholesterol
$-\log_{10}(p_y)$
POS
R^2
[0.0,0.2]
(0.2,0.5]
(0.5,0.8]
(0.8,1.0]
THAP6
USO1
SDAD1
PPEF2
SCARB2
NAAA
NUP54
G3BP2
FAM47E-STBD1
CXCL9
CXCL11
CXCL10
CDKL2
C4orf26
ART3
CCDC158
Chromosome 4 position (Mb)

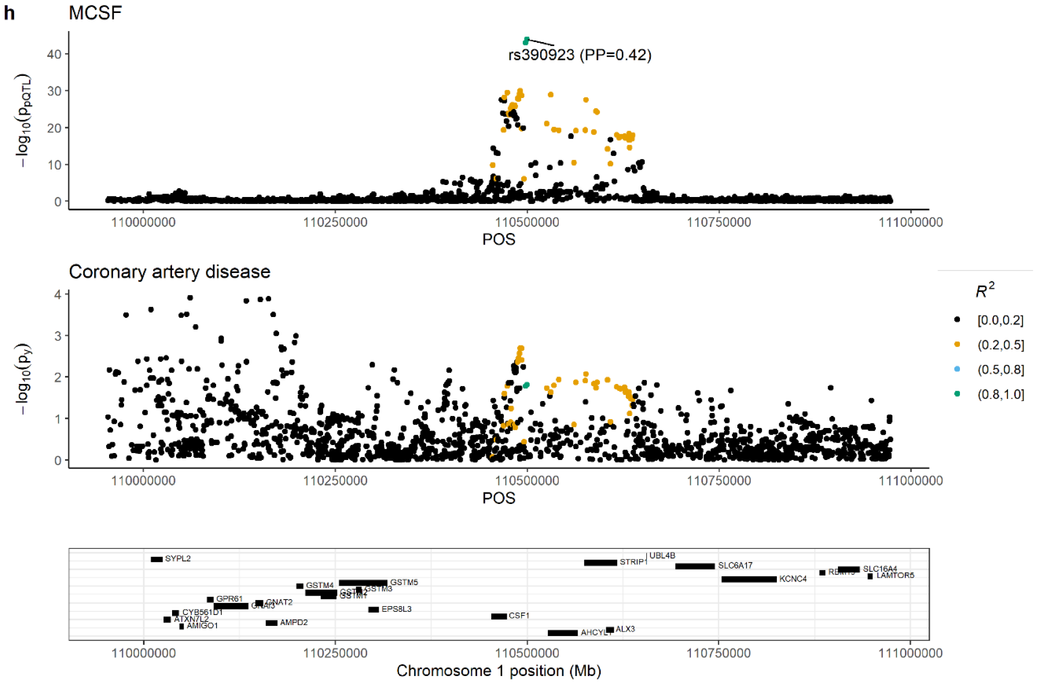
h
MCSF
rs390923 (PP=0.42)
$-\log_{10}(p_{pQTL})$
POS
Coronary artery disease
$-\log_{10}(p_y)$
POS
R^2
[0.0,0.2]
(0.2,0.5]
(0.5,0.8]
(0.8,1.0]
SYPL2
STRIP1
UBL4B
SLC6A17
KCNC4
SLC16A4
LAMTOR5
GSTM5
GSTM4
GSTM3
GPR61
GNAT2
EPS8L3
CSF1
AMPD2
AHCYL1
ALX3
ATXN7L2
AMIGO1
Chromosome 1 position (Mb)

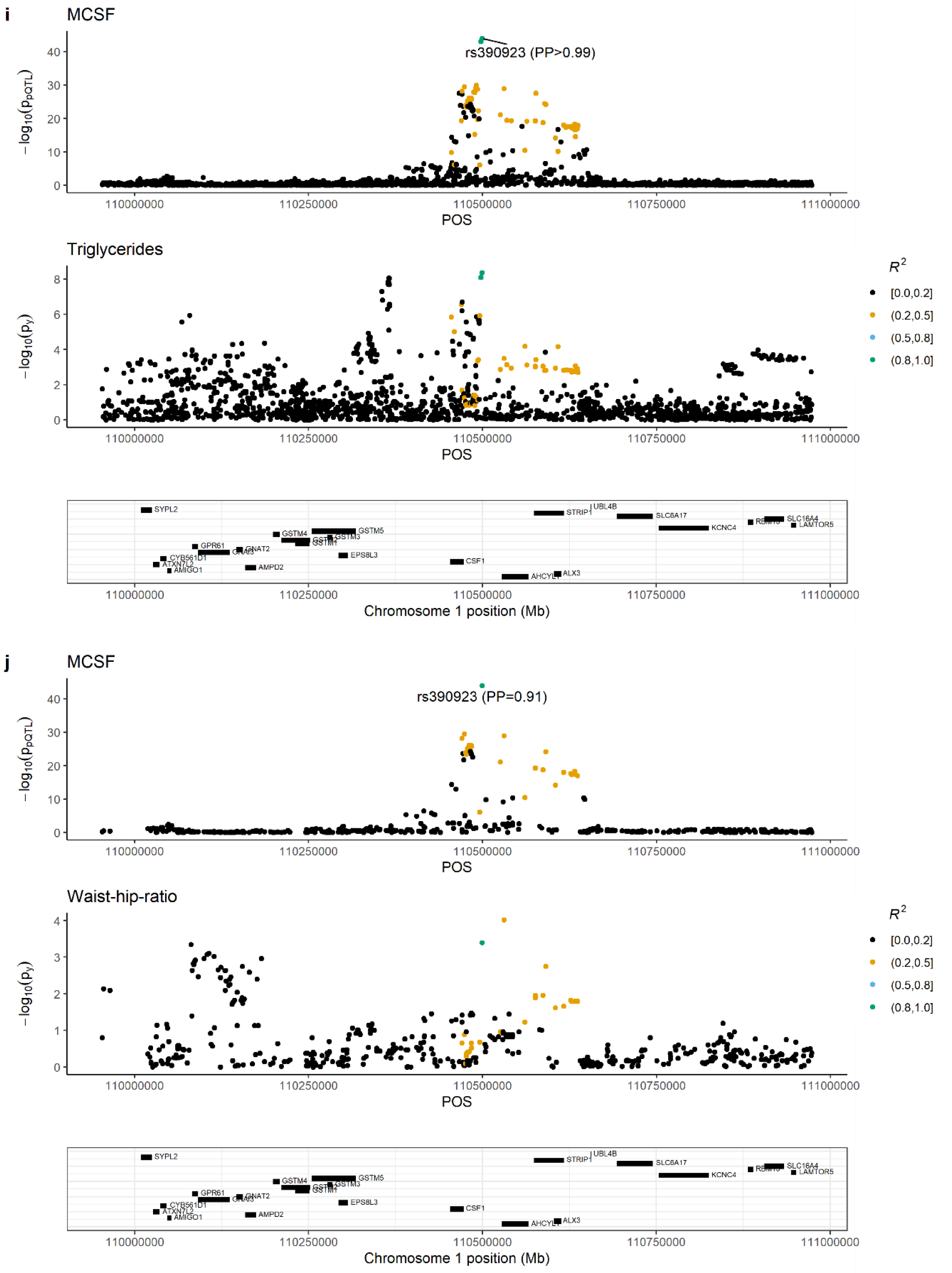
i
MCSF
rs390923 (PP>0.99)
$-\log_{10}(p_{pQTL})$
POS
Triglycerides
$-\log_{10}(p_y)$
POS
R^2
[0.0,0.2]
(0.2,0.5]
(0.5,0.8]
(0.8,1.0]
Chromosome 1 position (Mb)
j
MCSF
rs390923 (PP=0.91)
$-\log_{10}(p_{pQTL})$
POS
Waist-hip-ratio
$-\log_{10}(p_y)$
POS
R^2
[0.0,0.2]
(0.2,0.5]
(0.5,0.8]
(0.8,1.0]
Chromosome 1 position (Mb)

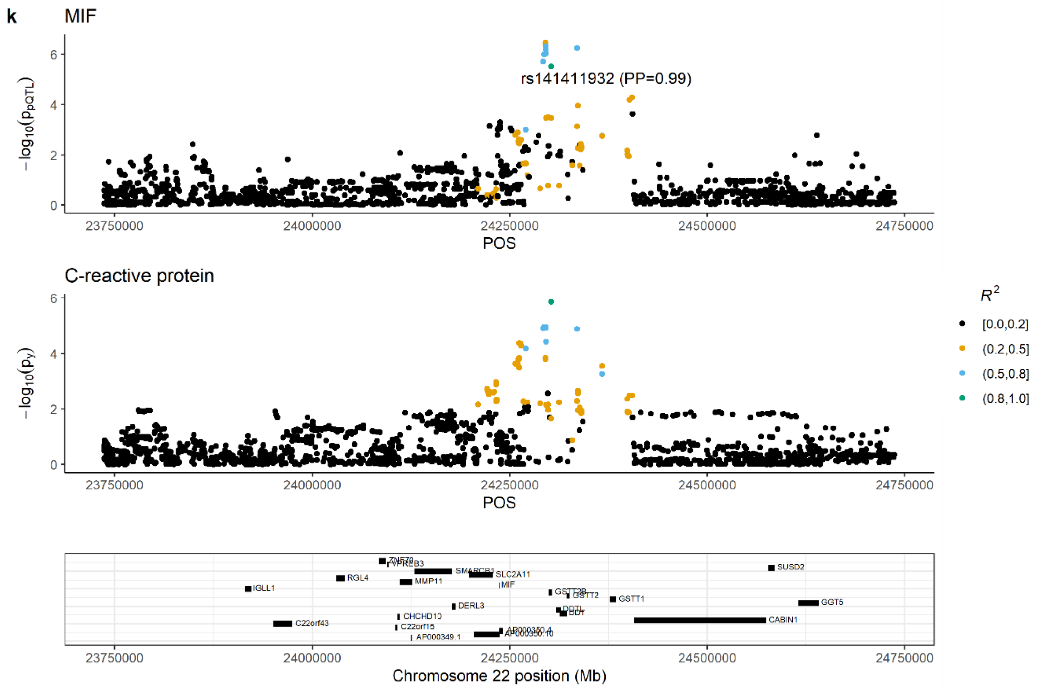
k
MIF
rs141411932 (PP=0.99)
$-\log_{10}(p_{pQTL})$
POS
C-reactive protein
$-\log_{10}(p_y)$
R^2
[0.0,0.2]
(0.2,0.5]
(0.5,0.8]
(0.8,1.0]
Chromosome 22 position (Mb)

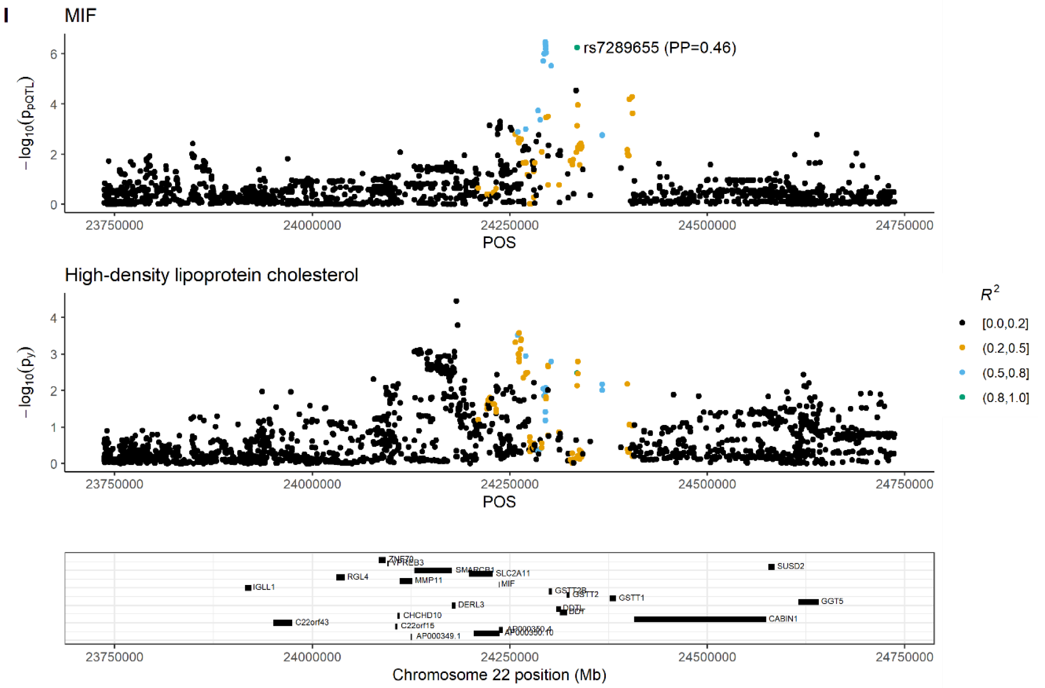
l
MIF
rs7289655 (PP=0.46)
$-\log_{10}(p_{pQTL})$
POS
High-density lipoprotein cholesterol
$-\log_{10}(p_y)$
R^2
[0.0,0.2]
(0.2,0.5]
(0.5,0.8]
(0.8,1.0]
Chromosome 22 position (Mb)

m

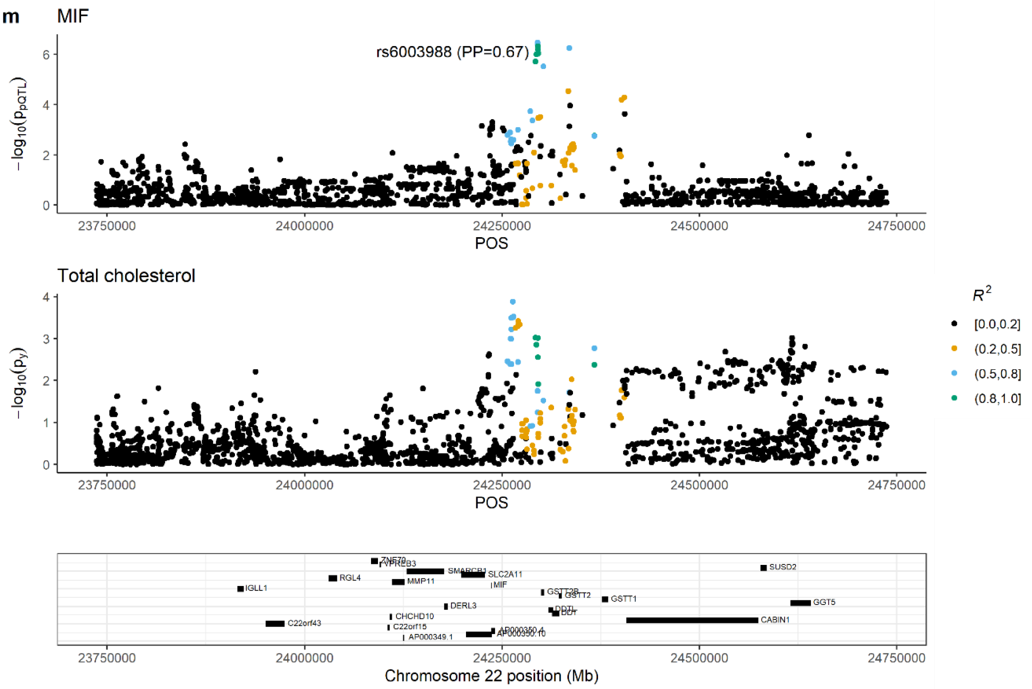
MIF
rs6003988 (PP=0.67)
−log10(ppQTL)
POS
Total cholesterol
−log10(py)
R2
[0.0,0.2]
(0.2,0.5]
(0.5,0.8]
(0.8,1.0]
23750000
24000000
24250000
24500000
24750000
Chromosome 22 position (Mb)
ZNF70
VPREB3
SMARCB1
SLC2A11
MIF
RGL4
MMP11
IGLL1
GSTT2B
GSTT2
GSTT1
SUSD2
GGT5
DERL3
DDTL
DDT
CHCHD10
C22orf15
C22orf43
AP000349.1
AP000350.4
AP000350.10
CABIN1

n

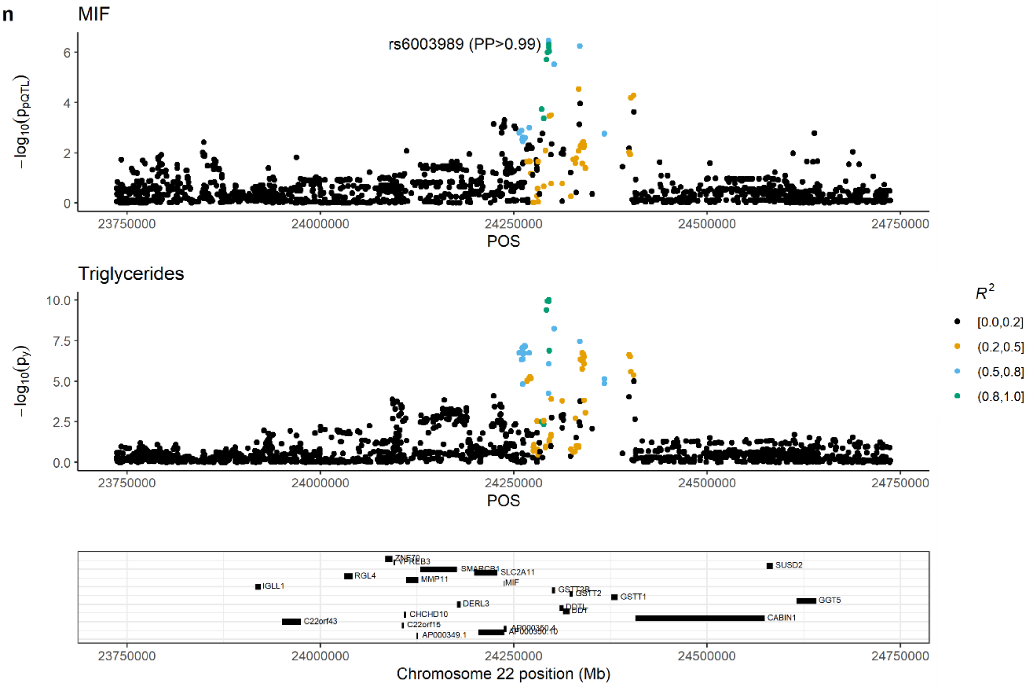
MIF
rs6003989 (PP>0.99)
−log10(ppQTL)
POS
Triglycerides
−log10(py)
R2
[0.0,0.2]
(0.2,0.5]
(0.5,0.8]
(0.8,1.0]
23750000
24000000
24250000
24500000
24750000
Chromosome 22 position (Mb)
ZNF70
VPREB3
SMARCB1
SLC2A11
MIF
RGL4
MMP11
IGLL1
GSTT2B
GSTT2
GSTT1
SUSD2
GGT5
DERL3
DDTL
DDT
CHCHD10
C22orf15
C22orf43
AP000349.1
AP000350.4
AP000350.10
CABIN1

o

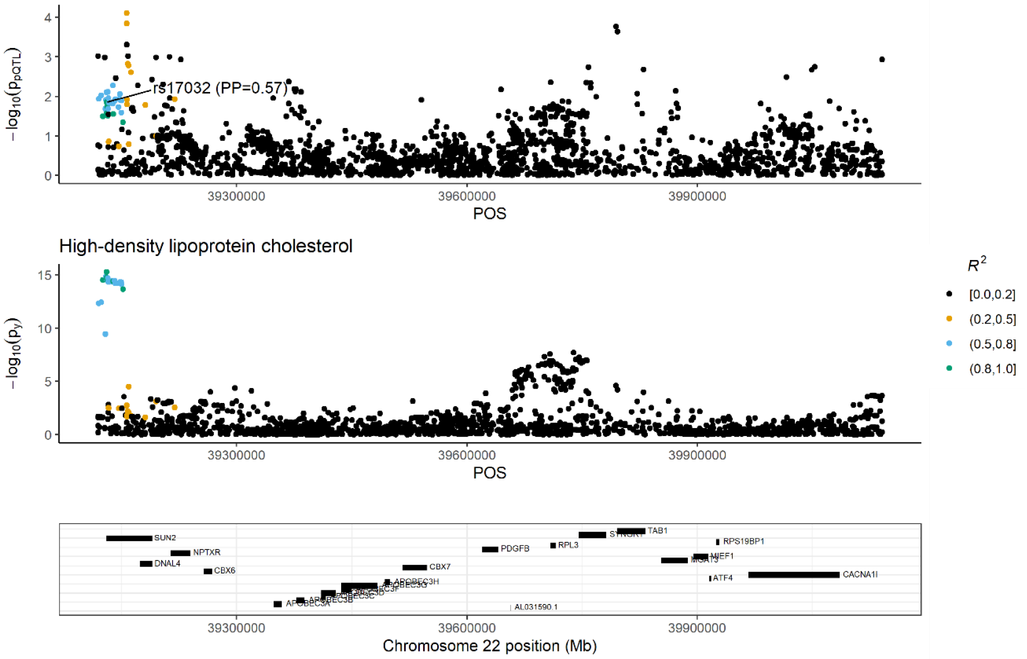
PDGFbb
rs17032 (PP=0.57)
POS
High-density lipoprotein cholesterol
POS
Chromosome 22 position (Mb)

p

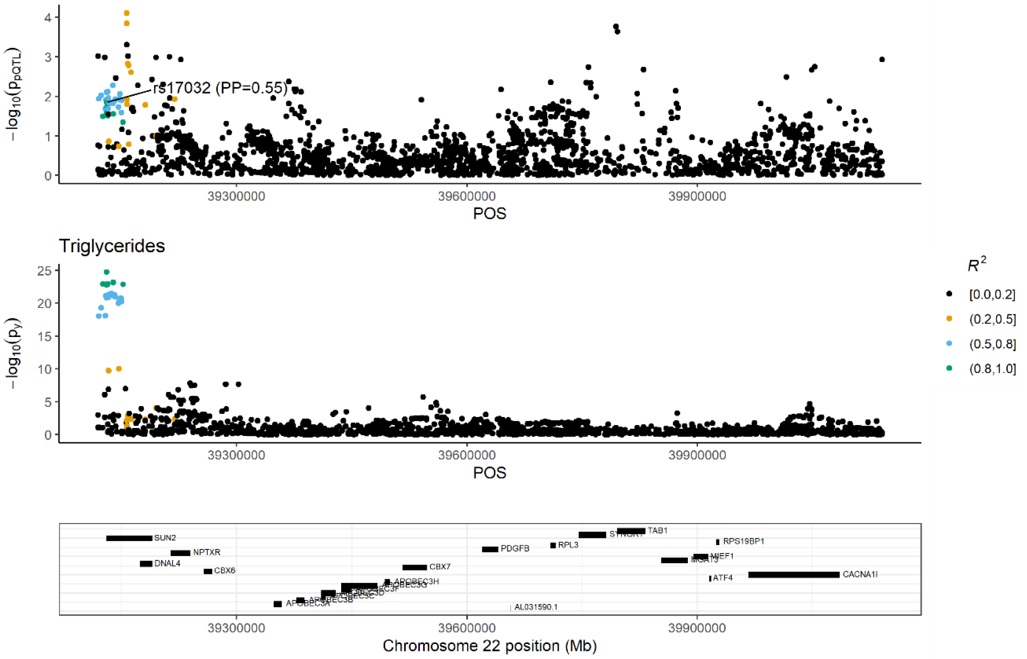
PDGFbb
rs17032 (PP=0.55)
POS
Triglycerides
POS
Chromosome 22 position (Mb)

q

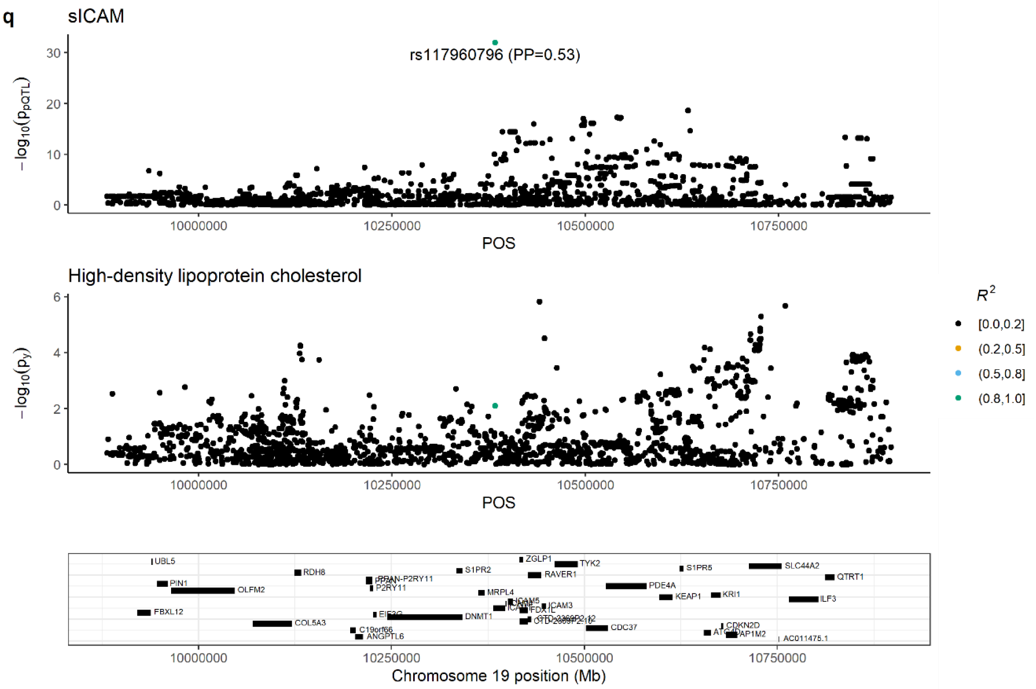
sICAM
rs117960796 (PP=0.53)
$-\log_{10}(p_{pQTL})$
POS
High-density lipoprotein cholesterol
$-\log_{10}(p_y)$
R^2
[0.0,0.2]
(0.2,0.5]
(0.5,0.8]
(0.8,1.0]
Chromosome 19 position (Mb)

r

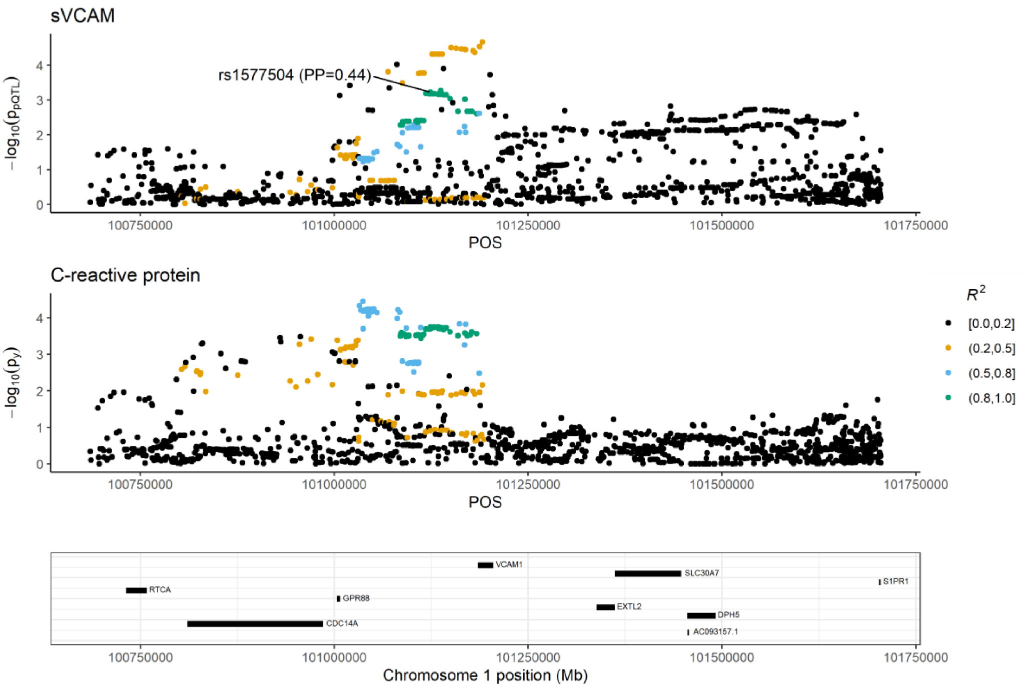
sVCAM
rs1577504 (PP=0.44)
$-\log_{10}(p_{pQTL})$
POS
C-reactive protein
$-\log_{10}(p_y)$
R^2
[0.0,0.2]
(0.2,0.5]
(0.5,0.8]
(0.8,1.0]
Chromosome 1 position (Mb)

s

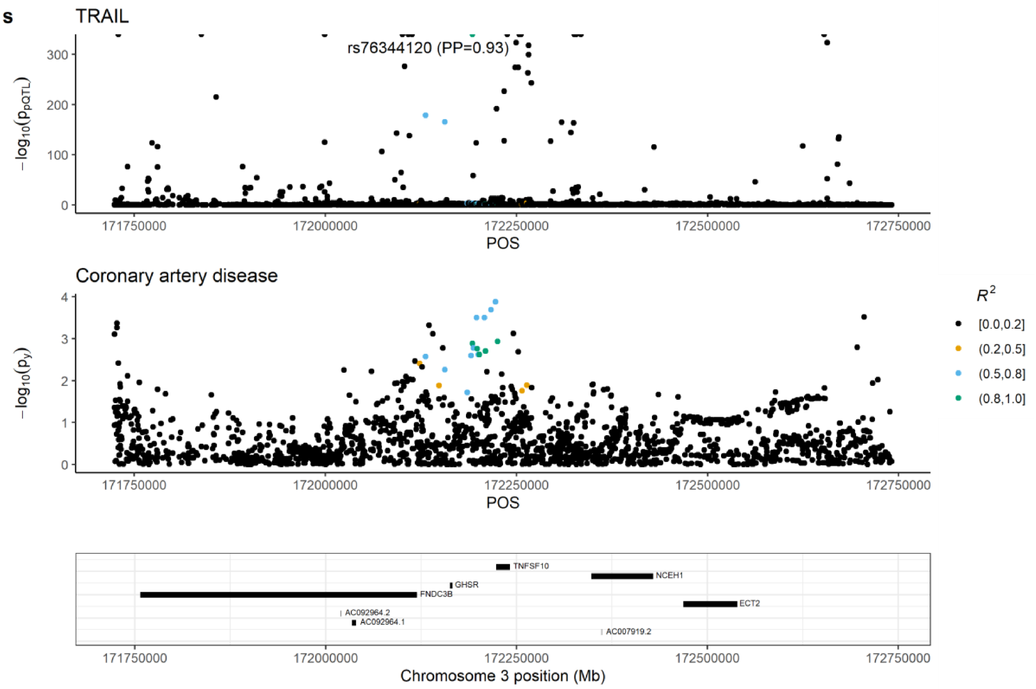
TRAIL
rs76344120 (PP=0.93)
−log10(p_pQTL)
POS
Coronary artery disease
−log10(p_y)
POS
R²
[0.0,0.2]
(0.2,0.5]
(0.5,0.8]
(0.8,1.0]
TNFSF10
NCEH1
GHSR
FNDC3B
ECT2
AC092964.2
AC092964.1
AC007919.2
Chromosome 3 position (Mb)

t

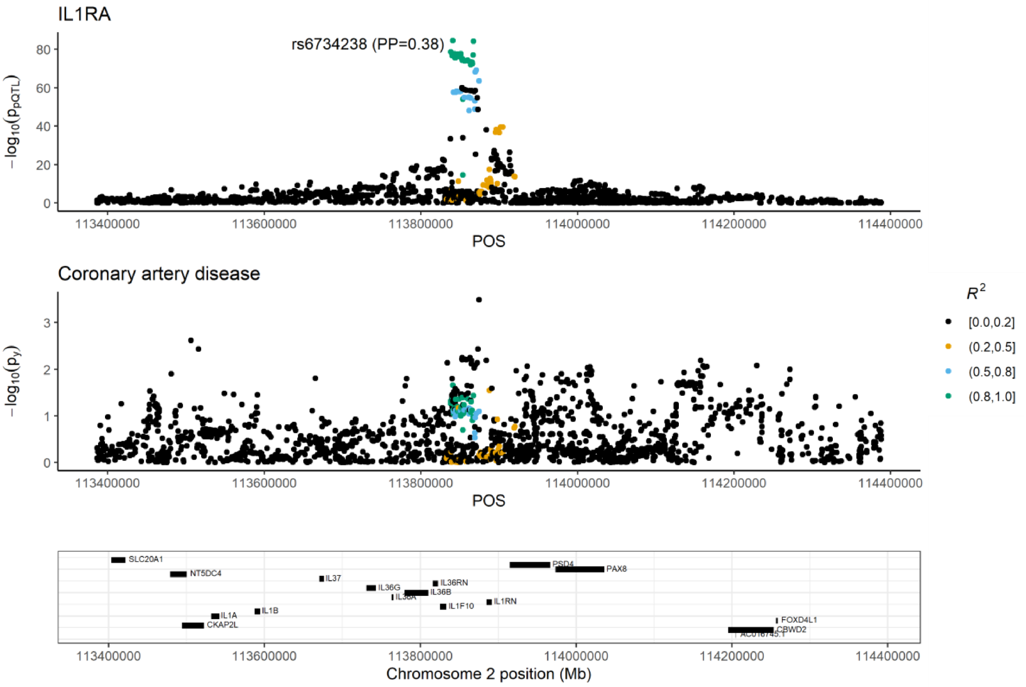
IL1RA
rs6734238 (PP=0.38)
−log10(p_pQTL)
POS
Coronary artery disease
−log10(p_y)
POS
R²
[0.0,0.2]
(0.2,0.5]
(0.5,0.8]
(0.8,1.0]
SLC20A1
NT5DC4
IL37
IL36G
IL36RN
IL36B
IL1F10
IL1RN
PAX8
IL1A
IL1B
CKAP2L
FOXD4L1
CBWD2
Chromosome 2 position (Mb)

Supplementary Figures 10a-t. Colocalization plots for circulating cytokine levels and cardiometabolic traits considered as outcomes.

Cytokine-outcome pairs with both Mendelian randomization evidence ($P<0.0033$) and colocalization evidence ($PP_{shared} + PP_{distinct} > 0.5$ and $PP_{shared} /(PP_{shared} + PP_{distinct}) > 0.5$; panels a-s) for causality, or only Mendelian randomization evidence for coronary artery disease risk (panel t) are plotted within ±500kb of the coding gene of the exposure cytokine. pQTL=protein quantitative trait loci. R^2 = linkage disequilibrium correlation based on the 1000Genomes European reference panel. PP = posterior probability.
